# Shadow enhanced self-charging power system for wave and solar energy harvesting from the ocean

**DOI:** 10.1038/s41467-021-20919-9

**Published:** 2021-01-27

**Authors:** Qian Zhang, Qijie Liang, Dilip Krishna Nandakumar, Hao Qu, Qiongfeng Shi, Fuad Indra Alzakia, Darrell Jun Jie Tay, Lin Yang, Xueping Zhang, Lakshmi Suresh, Chengkuo Lee, Andrew Thye Shen Wee, Swee Ching Tan

**Affiliations:** 1 Department of Materials Science and Engineering, National University of Singapore, 9 Engineering Drive 1, Singapore, 117574 Singapore; 2 Department of Physics, National University of Singapore, 2 Science Drive 3, Singapore, 117551 Singapore; 3 Department of Electrical and Computer Engineering, National University of Singapore, 4 Engineering Drive 3, Singapore, 117576 Singapore

**Keywords:** Devices for energy harvesting, Materials for energy and catalysis, Nanoscale materials

## Abstract

Hybrid energy-harvesting systems that capture both wave and solar energy from the oceans using triboelectric nanogenerators and photovoltaic cells are promising renewable energy solutions. However, ubiquitous shadows cast from moving objects in these systems are undesirable as they degrade the performance of the photovoltaic cells. Here we report a shadow-tribo-effect nanogenerator that hybrids tribo-effect and shadow-effect together to overcome this issue. Several fiber-supercapacitors are integrated with the shadow-tribo-effect nanogenerator to form a self-charging power system. To capture and store wave/solar energy from oceans, an energy ball based on the self-charging power system is demonstrated. By harnessing the shadow-effect, i.e. the shadow of the moving object in the energy ball, the charging time shortens to 253.3 s to charge the fiber-supercapacitors to the same voltage (0.3 V) as using pure tribo-effect. This cost-effective method to harvest and store the wave/solar energy from the oceans in this work is expected to inspire next-generation large-scale blue energy harvesting.

## Abstract (web summary)

Ubiquitous shadows cast from moving objects in hybrid energy-harvesting systems are undesirable as they degrade the performance of the photovoltaic cells. Here the authors report the shadow of the moving object in a hybrid energy-harvesting system shortens charging time to charge a self-charging power system.

## Introduction

The energy crisis and environmental concerns associated with the continuous combustion of fossil fuels have spurred the development of clean energy technologies for a sustainable society^1^. Wave energy^2^, as one form of water energy, is both a clean and renewable source with tremendous potential to generate electricity globally. If fully exploited, about 40% of the world’s power demand could be supplied by this resource, equivalent to about 800 nuclear power plants^3^. One potential method to harness ocean wave energy is to use the triboelectric nanogenerator (TENG), which offers many potential advantages^4,5^. However, the continuous outputs of small magnitude, pulsed signals, and low power output of TENG have necessitated the need for the development of hybrid energy systems that can provide a much-increased sustainable power output^6–8^. The oceans, covering more than 70% of the earth’s surface area, receive a large fraction of the incident solar radiation. Research on effectively utilizing both solar radiation and wave energy together is needed, but currently lacking. Such a technology, if developed to a commercial scale, holds immense potential for solving the world energy crisis, and ensuring energy security for all. So far, hybrid energy systems based on triboelectric-photovoltaic use transparent objects (rain, wind) that harness friction^9,10^ or position the photovoltaic cells at locations without shadows from moving objects^11^. One of the reasons impeding the development of triboelectric-photovoltaic hybrid technology is the presence of shadows that decreases the output of the photovoltaics^12,13^.

When two objects in different positions in the triboelectric series are brought together, triboelectric charges build up on the two surfaces in physical contact^14^. Shadows are cast if the moving object on the top is opaque. Ravi et al. reported that it is possible to tune the work function of a metal/semiconductor junction by illuminating light on the junction, resulting in a directional flow of electrons from the bright zone to the dark zone^15–17^. Scavenging the illumination contrast that arises on the surface underneath and generating power from this effect is an effective solution to harvest solar energy.

Harvesting the energy from oceans is challenging due to the dramatic changes in the environment which leads to a great deal of intermittency^18,19^. Zhong Lin Wang and his co-workers demonstrated a feasible approach to harness wave energy through triboelectric effect at the solid-liquid interface using a single component system capable of generating a continuous DC output^20^. Another approach by the same group to improve the current produced was to integrate nanoparticle surface modifications with triboelectric effect resulting in a 9.8% energy conversion efficiency^21^. However, for any microelectronics to be successful it needs innovation in both architectural design and operation. Zou et al. reported the efficient coupling of supercapacitors with TENGs to make a biomimetic pressure sensor for simultaneous energy conversion, energy storage, pressure sensor and counter^22^. Of which an energy storage component is very critical for power management and to regulate the energy supply^23,24^. Supercapacitors are more rugged and last hundreds to thousands of times longer than the average chemical reaction-based batteries^25^. This cyclic stability of supercapacitors makes them an ideal choice of energy storage for effective power management systems. The performance of supercapacitors is largely dictated by their active electrode materials^26^. Among them, molybdenum disulfide (MoS_2_), with a few-layered structure, is preferred as an electrode material due to its high specific surface, interlayered space for ion intercalation, and a wide range of metal ion oxidation states^27,28^.

In this work, we present a self-charging power system by integrating a shadow-tribo-effect nanogenerator (S-TENG) and fiber-supercapacitors (F-SCs) with few-layered MoS_2_ as the active material. The S-TENG is a hybrid energy harvester which captures energy by applying shadow-effect (SE) and tribo-effect (TE) in one device. For practical applications in the oceans, the self-charging power system is demonstrated as an energy ball. The S-TENG in the energy ball produces electricity by making use of shadow of moving object and the mechanical stimulation of wave. In a typical ocean under the sunshine condition, the S-TENGs generate a peak power output of 0.69 MW km^−2^. With the integration of the F-SCs, the converted energy from S-TENG is stored in the self-charging power system directly. The shadow of the moving object shortens the charging time of the energy ball significantly. The S-TENG with simple structure and high stability will find numerous applications in the future, such as seawater electrolysis to produce hydrogen.

## Results

### Structure design of the energy ball and band gap diagram of shadow-effect

**Fig. 1:**
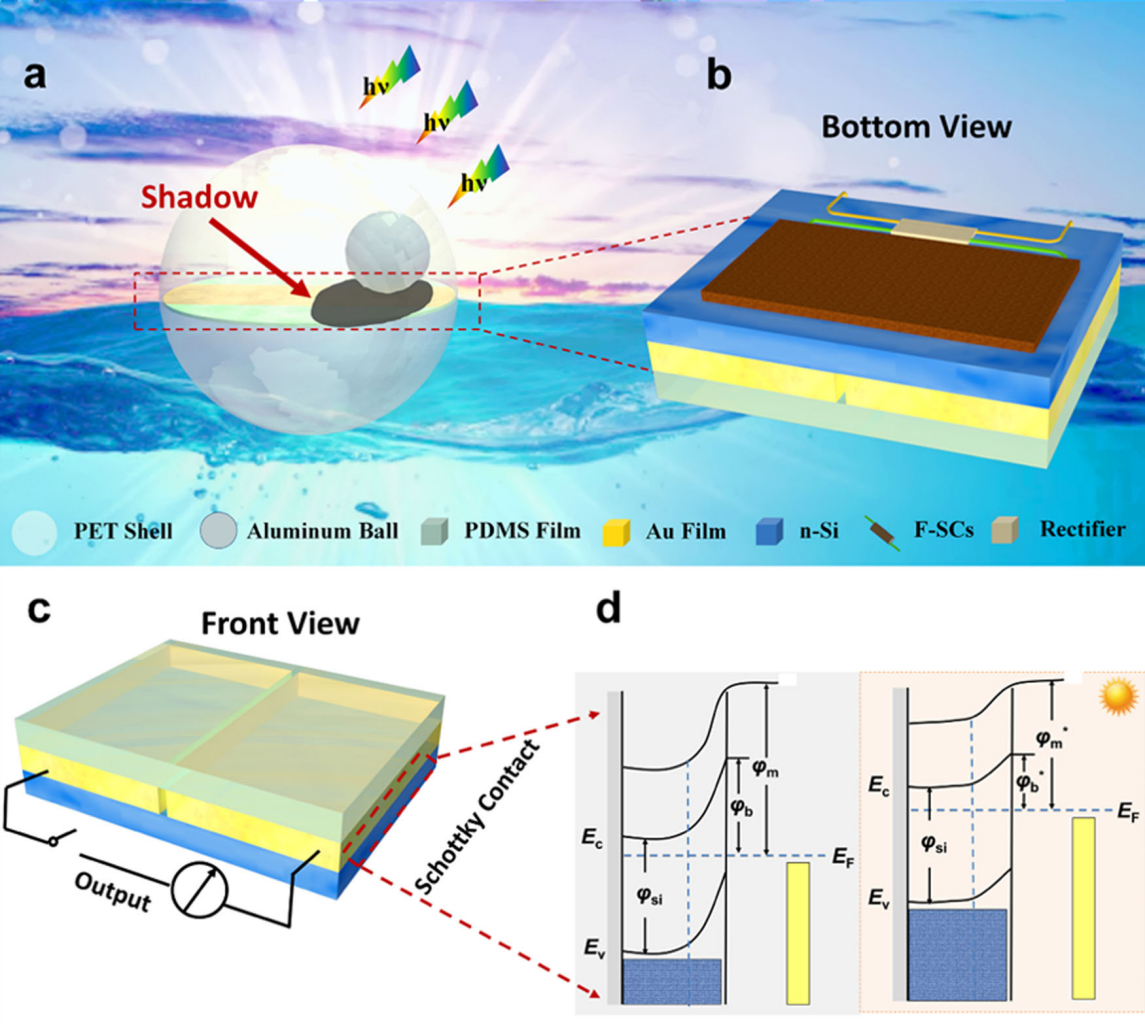
Structure design of energy ball and energy band diagram of the shadow-effect. **a** Schematic of the energy ball (diameter is 8 cm, F-SCs is short for fiber-supercapacitor) harvesting both solar energy and wave energy from ocean. Schematic illustration showing the configuration of the shadow-tribo-effect nanogenerator based self-charging power system: **b** bottom view and **c** front view. **d** Representative energy band diagram of Au/n-Si system.

A schematic illustration of the energy ball (diameter is 8 cm) floating in the ocean and illuminated by the sun is shown in Fig. 1a. The energy ball consists of three main parts: (i) A S-TENG based self-charging power system is fabricated for energy harvesting and storage. (ii) A small aluminum (Al) ball which is used as the opaque moving object on the S-TENG. (iii) A transparent, round PET shell to cover parts (i) and (ii) inside. The detailed structure of the S-TENG based self-charging power system which integrates the S-TENG and F-SCs is shown in Fig. 1b, c. The photograph of the as fabricated energy ball is shown in Supplementary Fig. 1 and detailed description is presented in Supplementary Note 1. The S-TENG is a hybrid energy harvester which is used to convert the solar energy and mechanical energy continuously. Another important component of the self-charging power system is F-SCs with a few-layered MoS_2_ as active material. The S-TENG utilizes an Au/n-Si system (with a 1 cm gap in middle of Au film) to collect light energy by the SE arising due to illumination contrasts while a polydimethylsiloxane (PDMS) film deposited on the Au/n-Si system acts as the friction layer. PDMS is chosen here not only because it is positioned in the extreme negative end in the triboelectric series, but it is also transparent^29^. Figure 1d shows the energy band diagrams of the Au/n-Si system. As the Au layer (work function *φ*_m_) is continuous, a Schottky barrier, which is verified and tested in Supplementary Fig. 2 and detailed discussion is presented in Supplementary Note 2, is created on the whole surface of the electrode: a depletion layer is created inside the n-Si (work function *φ*_Si_) and the bands are bent after equilibration as shown. The energy barrier at the Au/n-Si interface *φ*_b_ is simply the difference between the *φ*_Si_ and *φ*_m_^30^. Under illumination, the photo-generated electrons and holes are separated efficiently by the strong electric field generated in the depletion region. During continuous illumination of the entire electrode, the accumulated electrons overcome the Schottky barrier and are injected into the metal. These injected electrons lower the work function of the metal (*φ*_m_^*^) in the illuminated condition when compared to the dark condition *φ*_m_.

### Characteristics of the shadow-effect

**Fig. 2:**
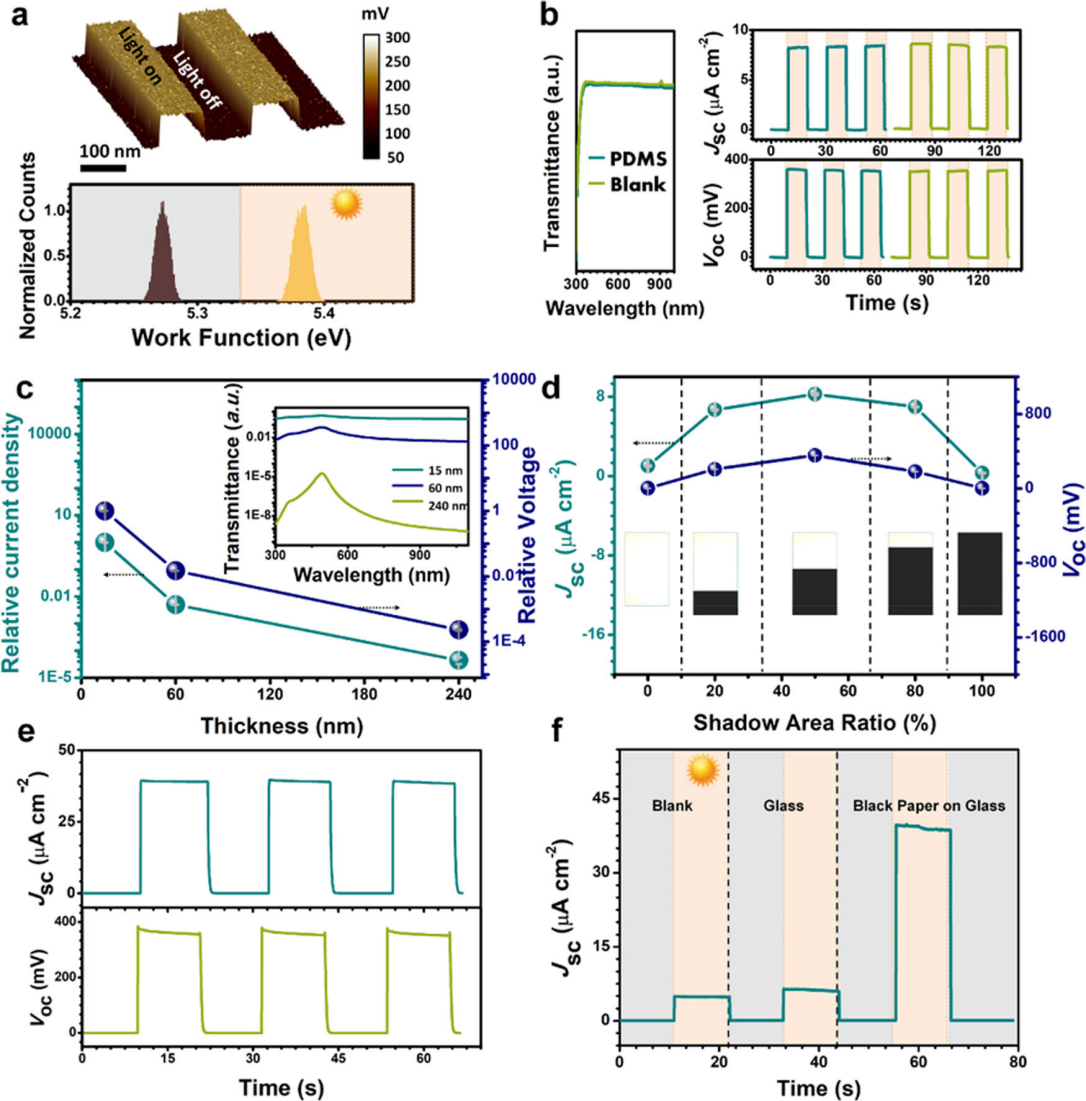
Characteristics of the shadow-effect. **a** Surface potential map with two light on/light off cycles and work function shift of the dark and illumination (20 mW cm^−2^). **b** UV-vis transmittance spectra, short-circuit current density (*J*_sc_) and open-circuit voltage (*V*_oc_) of the Au/n-Si system without (blank) and with PDMS film. **c** The Relative current density (*J*_15_*/J*_15_*, J*_60_*/J*_15_*, J*_240_*/J*_15_) and relative voltage (*V*_15_*/V*_15_*, V*_60_*/V*_15_*, V*_240_*/V*_15_) versus the thickness of Au film of the Au/n-Si system. Inset is transmittance of the Au film with different thicknesses. **d** Effect of the ratio of shadow area on performance of Au/n-Si. **e** *J*_sc_ and *V*_oc_ of the Au/n-Si system after removing 1 cm Au film in middle. **f** Performance of Au/n-Si system after removing middle Au film half-shadowed by different shadow-casting objects (blank, glass, black paper on glass).

Atomic force microscopy (AFM) and kelvin probe force microscopy (KPFM) were adopted to characterize the surface property of Au/n-Si surface and confirm the work function shift under dark and illuminated conditions. The roughness of Au/n-Si surface was obtained by a Pt/Ir tip in the contact mode of AFM, the results are shown in Supplementary Fig. 3 and related discussion is presented in Supplementary Note 3. Then the KPFM scan was performed under two distinct conditions-light on and light off which signifies the solar illuminated and dark conditions. As shown in Fig. 2a, a higher surface potential was observed under illumination. The work function of surface (*φ*_surface_) before and after illumination can be calculated as:

1 $$\varphi _{{\mathrm{surface}}} = \varphi _{{\mathrm{tip}}} - {\mathrm{CPD}} \times e^ -$$

CPD is contact potential difference, *φ*_tip_ is the work function of the Pt/Ir tip which was used for the KPFM scan. The work function of Au/n-Si surface shifted from 5.26 eV to 5.39 eV under light illumination (20 mW cm^−2^). The detailed surface potentials of Au/n-Si with 15 nm Au film under dark and illumination conditions are also shown in Supplementary Fig. 3.

The SE is that the difference in surface potential which is generated between the illuminated and dark regions when it is partially illuminated with light. In other words, when shadows fall partially on the S-TENG, the ensuing illumination contrasts will be converted into electrical signals.

To characterize the influence of PDMS on the transparency of Au/n-Si system, UV-vis spectroscopy was used to study the transmittance of the PDMS on a glass. As shown in Fig. 2b, the pane without and with PDMS film showed an unchanged transmittance in the visible and near-infrared region. The short-circuit current density (*J*_sc_) and open-circuit voltage (*V*_oc_) of the half-shadowed Au/n-Si system in dark and under illumination (100 mW cm^−2^) without and with PDMS were also characterized. The *J*_sc_ and *V*_oc_ of about 8.5 μA cm^−2^ and 359.8 mV were observed, respectively, when one-half of the Au/n-Si surface was shadowed by a black paper. All of the area calculated in this work is total area (both shadowed and illuminated parts). With the thickness of Au film on n-Si changing from 15, 60, to 240 nm, the relative current density *J*_15_/*J*_15_, *J*_60_/*J*_15_, *J*_240_/*J*_15_ and relative voltage *V*_15_/*V*_15_, *V*_60_/*V*_15_, *V*_240_/*V*_15_ are shown in Fig. 2c. *J*_15_, *J*_60_, *J*_240_ are short-circuit current densities and *V*_15_, *V*_60_, *V*_240_ are open-circuit voltages of Au/n-Si system with different thickness of Au film. All of these currents and voltages of Au/n-Si systems are measured under 1 sun illumination (100 mW cm^−2^) and half-in-shadow by black paper. The current and voltage generated by Au/n-Si with 15 nm Au film are much larger than that generated by Au/n-Si system with 60 nm and 240 nm. The transmittance of the Au films with 15 nm, 60 nm, and 240 nm are shown in the inset of Fig. 2c. The transmittance of Au film decreases with the increasing thickness, which limits the light reaching n-Si and thus the number of photocarriers generated. Therefore, the 15 nm Au film was adopted to fabricate the S-TENG. The electrical resistance of Au film with 15 nm, 60 nm, 240 nm are also tested and shown in Supplementary Fig. 4. The related discussion is presented in Supplementary Note 4. In addition, the shadowed area also influences performance of the Au/n-Si system. In Fig. 2d, the *J*_sc_ and *V*_oc_ of the Au/n-Si under a shadow area ratio (i.e., the shadowed area divided by total area) of 50% are all larger than that of 0%, 20%, 80%, and 100%. The inset in Fig. 2d is the picture of Au/n-Si system under black paper with different area ratio. The half-shadowed condition of the Au/n-Si system provides an optimum surface area for electron generation and electron collection. As shown in Supplementary Fig. 5, a PDMS film on the Au/n-Si system could be applied using a traditional single-electrode mode. A stable output of PDMS/Au/n-Si system with droplet contact-separation on it continuously proves that the PDMS cover on the Au/n-Si system could generate power by a TE. The detailed discussion is presented in Supplementary Note 5.

To fabricate the S-TENG, the Au film of about 1 cm width in the middle of n-Si was removed. The measurements of *J*_sc_ and *V*_oc_ after removing Au film were made to determine the SE (Fig. 2e). Comparing *J*_sc_ and *V*_oc_ before removing Au film in Fig. 2b, there is no obvious change in *V*_oc_ while *J*_sc_ greatly increases from 8.5 μA cm^−2^ to 39 μA cm^−2^ after Au film in middle was removed and the device was placed half-in-shadow by the same black paper. From the SE working mechanism^16^, it is the light illumination that causes the excitation of electrons and the work function shift. As there is no change in excitation of electrons and work function shift of Au film with or without a gap, the *V*_oc_ keeps unchanged. However, the overall device *J*_sc_ of the device depends on two factors that are electron generation and electron transport to the dark side from where it can be collected. In original situation (Au film unremoved), parts of the excited electrons by SE flow to dark side directly on Au film, thereby leading to a decline in *J*_sc_ of the device. In case of the middle Au film removed, the electrons can only flow from external circuit which makes the electron transfer more efficiently. To further verify that the SE still works after middle Au film was removed, a transparent glass with and without black paper on it was used to cast shadow on the Au/n-Si with a gap. The transmittances of the transparent glass without and with a black paper on it are 80% and 0%, respectively. (Supplementary Fig. 6 and Supplementary Note 6) As there is less light passing through glass with a black paper on it, the *J*_sc_ of the Au/n-Si with a gap in the half-in-shadow condition is larger than that without black paper on the transparent glass. (Fig. 2f) This is because that illumination contrast between shadow region and illuminated region of Au/n-Si with a gap under glass with a black paper is much larger than under transparent glass without a black paper. In another words, darker shadow improves output of SE, which works after middle Au film being removed.

### Working principle of the S-TENG and output performance of the S-TENG

**Fig. 3:**
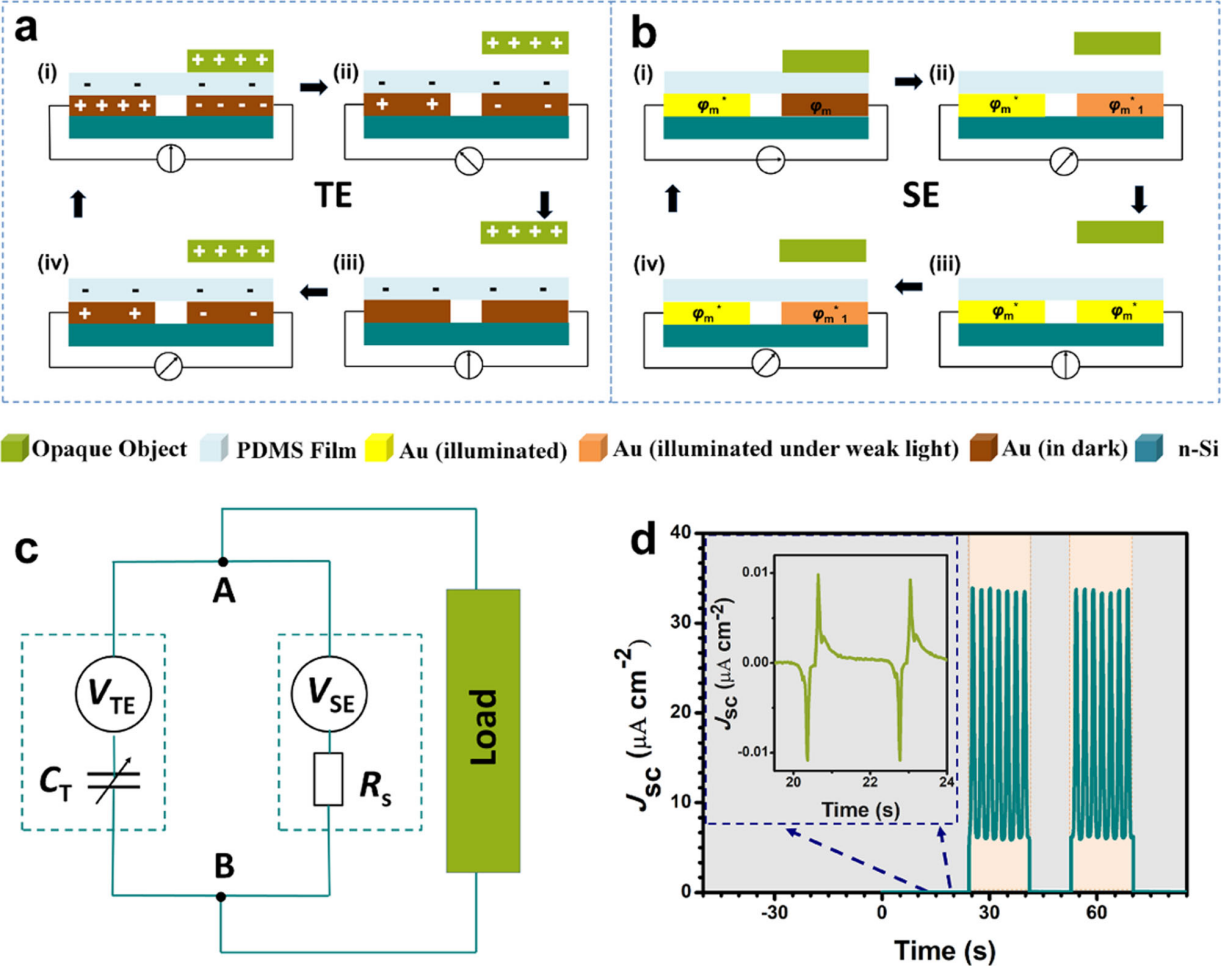
Working principle and typical output of the shadow-tribo-effect nanogenerator (S-TENG). Step-by-step illustration showing the **a** tribo-effect (TE) and **b** shadow-effect (SE) working principle of the S-TENG under dark and illumination conditions. **c** Equivalent circuit model of S-TENG hybrid TE and SE. **d** short-circuit current density (*J*_sc_) of the S-TENG in dark and illumination by moving stage of force gauge up and down. Inset is the details of the output performance from 19.5 s to 24 s.

The working mechanism of S-TENG which is a hybrid TE and SE nanogenerator is schematically depicted in Fig. 3a and b. In a simplified model, the equivalent circuit of the S-TENG with an ammeter is illustrated. Under dark conditions, the working mechanism of S-TENG is mainly based on TE. After an opaque object (such as the moving stage of a force gauge) is brought into transient physical contact with the S-TENG, the PDMS retains a layer of negative surface charges that do not dissipate over an extended period of time^31^. When the opaque object is reaching the surface of S-TENG, the positive charges screen the negative triboelectric charges on the PDMS surface. Opposite polarity charges are induced on Au electrodes (Fig. 3a (i)). As shown in Fig. 3a (ii), when the opaque object leaves S-TENG, unbalanced electric potential between the two Au electrodes drives free electrons to flow from right electrode to left electrode. As the opaque object keeps moving up from the surface of the PDMS, the electrons between electrodes achieve equilibrium (Fig. 3a (iii)). As the opaque object moves down again, free electrons flowing from left electrode to right electrode. (Fig. 3a (iv)). If the opaque object moves up and down periodically, continuous electric output could be generated. Thus, the S-TENG produces an alternative current (AC) pulse output during a periodic contact with an opaque object under dark condition.

With light illumination, the working mechanism of SE is shown in Fig. 3b. The opaque object (such as moving stage of a force gauge) is reaching the surface of S-TENG and casts a shadow on half of the surface area of the S-TENG, the work function of Au electrode under opaque object’s shadow is *φ*_m_, which is higher than that of the illuminated left electrode (*φ*_m_^*^). The electrons in n-type silicon below the Au film is continuously excited into the conduction band because of the continuous illumination. These electrons will then jump into the gold film and the work function contrast will drive the electrons to flow from the bright side to the dark side forming a continuous current through external circuit (Fig. 3b (i)). In stage (ii), when the opaque object moves away from S-TENG, light illuminates on all S-TENG, the illumination contrast between left and right part of S-TENG decreases. Work function of right electrode shifts to *φ*_m_^*^_1_, which is lower than *φ*_m_ while higher than *φ*_m_^*^. There are less free electrons flowing from the right electrode to left electrode than (i). As the opaque object keeps moving away, the work functions of electrodes all reach *φ*_m_^*^ and there are no more electrons transferred (Fig. 3b (iii)). As the opaque object moves down again, free electrons flow from right electrode to left electrode. (Fig. 3b (iv)). The S-TENG engenders a direct current (DC) output during a periodic up and down movement of an opaque object under illumination.

The equivalent circuit model of S-TENG which hybrids TE and SE is shown in Fig. 3c. As these two effects share the same electrodes, they are connected in parallel. In TE case, the TENG can be viewed as a capacitor (*C*_T_) using the capacitor model^14^. The equivalent circuit of SE is an open-circuit voltage source connected with a resistance (*R*_s_). The whole circuit also satisfies Thevenin’s Theorem, whose output voltage (*V*_AB_) is:

2 $$V_{{\mathrm{AB}}} = - \frac{Q}{{C_{\mathrm{T}}}} + V_{{\mathrm{TE}}} = V_{{\mathrm{SE}}} - IR_{\mathrm{s}}$$

Where *Q* is transferred charges, *V*_TE_ is open-circuit voltage of S-TENG with TE, *V*_SE_ is open-circuit voltage of S-TENG with SE, *I* is current in circuit without load.

As shown in Fig. 3d and Supplementary Movie 1, the *J*_sc_ measurement was carried out by moving stage of a force gauge towards and away from the S-TENG to control the experiment condition. A lamp was used as the light source. Under dark condition, a typical tribo-induced AC signal with peak *J*_sc_ about 0.01 μA cm^−2^ was generated. Once illuminated, the S-TENG generated a DC signal with peak *J*_sc_ about 34 μA cm^−2^. A light intensity of about 90 mW cm^−2^ was used for illumination. The magnified detailed wave forms of the output are provided in Supplementary Fig. 7 and related discussion is presented in Supplementary Note 7. An opposite current direction of TE and SE could be generated when they work simultaneously under the light condition. However, the current generated from SE is much larger than TE, so the S-TENG which coupled TE and SE is in the same current direction of SE.

**Fig. 4:**
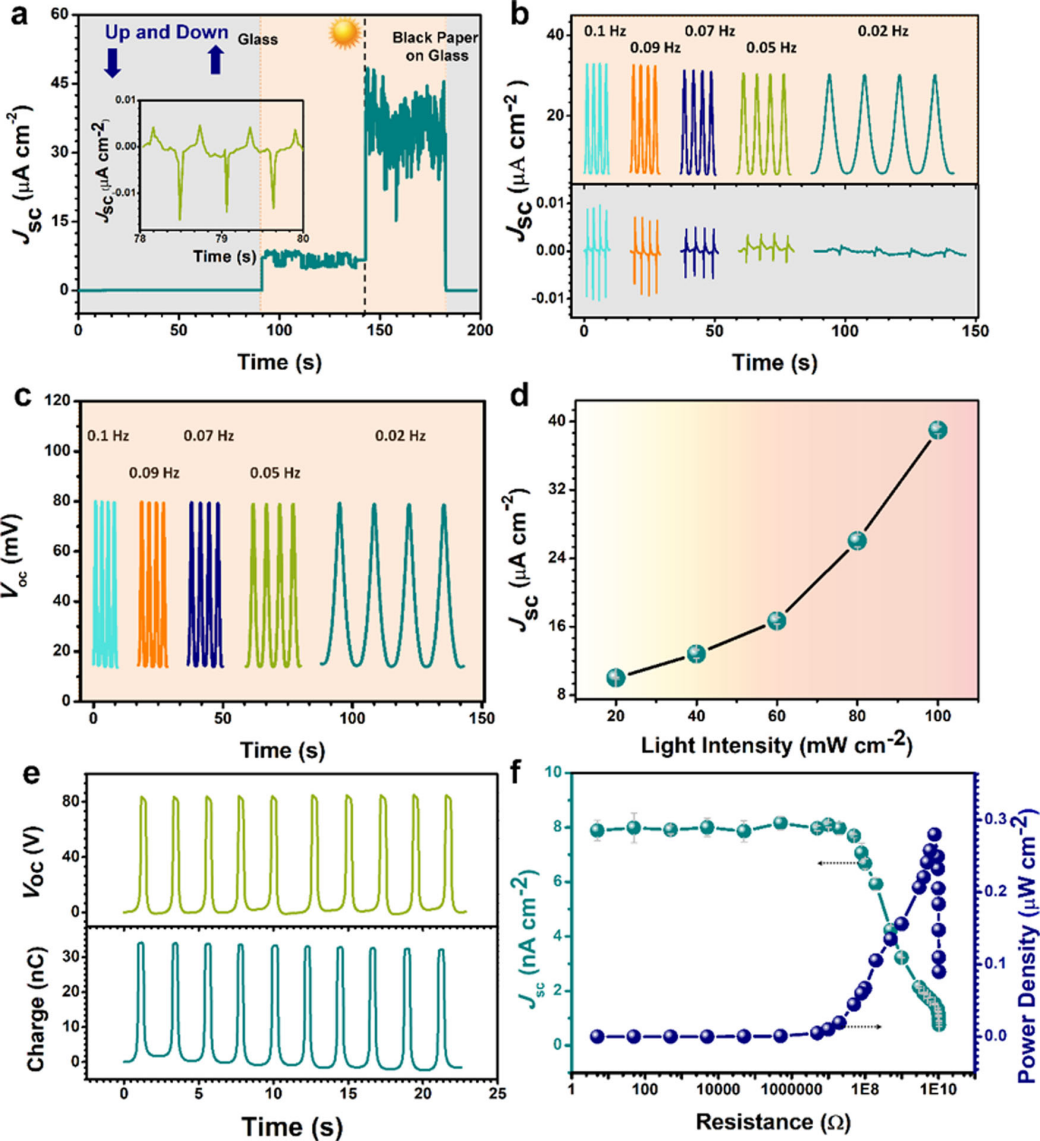
Output performance of the shadow-tribo-effect nanogenerator (S-TENG). **a** Glass with or without black paper moved on it up and down above half of S-TENG. Inset is the details of the output performance from 78 s to 80 s. **b** Influence of the frequency of the moving stage up and down on the short-circuit current density (*J*_sc_) under dark and illumination conditions. **c** Influence of the moving stage up and down frequency on open-circuit voltage (*V*_oc_) under illumination conditions. **d** Dependence of light intensity on output of S-TENG. **e** The *V*_oc_ and charge output of the S-TENG in a single-electrode TENG connection. **f** Output power density of the S-TENG in a single-electrode TENG connection with different load resistances.

A transparent glass with or without black paper on it was moved up and down over the S-TENG. The contact area was 7.5 cm^2^. Under dark condition, a peak *J*_sc_ about 0.005 μA cm^−2^ was generated. (insert in Fig. 4a). As shown in Fig. 4a, under illumination condition, the peak *J*_sc_ increased to about 8.2 μA cm^−2^ when the S-TENG was partially covered by transparent glass. When the S-TENG was partially covered by glass with black paper on it, the peak *J*_sc_ increased to about 43 μA cm^−2^. The only difference between these two friction objects is transmittance. The moving object casting darker shadow improves output of S-TENG to be larger, which is consistent with Fig. 2f. The outputs of S-TENG in various contact frequencies ranging from 0.02 Hz to 0.1 Hz under dark and illumination conditions are shown in Fig. 4b, respectively. The frequencies were determined by programming the force gauge. The *J*_sc_ of the S-TENG increases obviously with the frequency increases under dark condition. The current density of the S-TENG under dark condition based on TE can be written as:

3 $$J_{{\mathrm{sc}}} = d\Delta \sigma _{{\mathrm{sc}}}/dt = (d\Delta \sigma _{{\mathrm{sc}}}/dx) \times \nu$$

Where *x* is the displacement, *ν* is the variation velocity which is a measure of frequency, Δ*σ*_sc_ is the short-circuit transferred charges. Under illumination condition, there is no obvious change on *J*_sc_ and *V*_oc_ as the frequency changes (Fig. 4c). Figure 4d shows the output performance of the S-TENG under diverse light intensity. Clearly, when the light intensity is increased, the *J*_sc_ is enhanced. This is because n-Si is the main source of photocarrier generation. With a higher light intensity, more photocarriers in n-Si were generated.

As the TE and SE of S-TENG are connected in parallel, the *J*_sc_ of TE can be tested in a dark condition while the *V*_oc_ cannot be directly measured. This is because the parallel connection of the SE has a much lower internal resistance than TE. However, the SE part cannot be removed to test TE. So, a connection change method is used to test the output of a single-electrode TENG under the same condition. The result is expected to roughly estimate the output of TE which is based on a single-electrode mode. The positive electrode of Keithley 6514 was connected with one electrode of S-TENG and the negative electrode Keithley 6514 was grounded. The measurements were all carried out by moving stage of a force gauge towards and away from the S-TENG in a single-electrode TENG connection for accurate and repeatable motion control. As shown in Fig. 4e, the *V*_oc_ and charge is 80 V and 34 nC, respectively. Figure 4f demonstrates the dependence of output peak current density and peak power density on load resistance ranging from 5 Ω to 10.5 GΩ. The maximum power is about 0.28 μW cm^−2^ when the resistance is 8 GΩ.

### Performance of the F-SCs

**Fig. 5:**
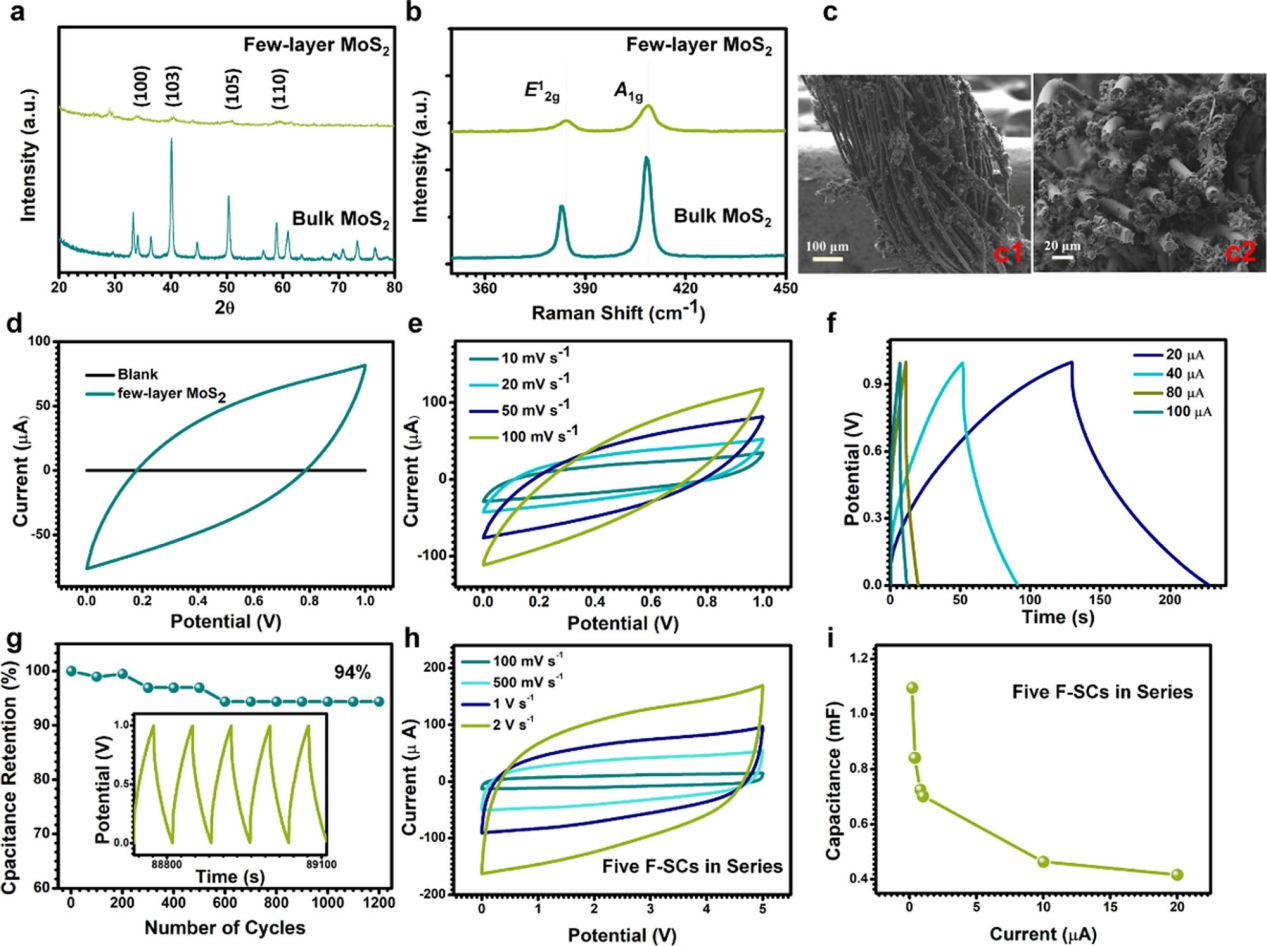
The performance of fiber-supercapacitors (F-SCs). **a** X-ray diffractometry and **b** Raman patterns of a few-layered MoS_2_ and bulk MoS_2_, respectively. **c** Scanning electron microscopy images of the few-layered MoS_2_ dipped carbon fiber (**c1** is top view and **c2** is cross view). **d** Cyclic voltammetry (CV) curves at a scan rate of 50 mV s^−1^ of F-SC with and without few-layered MoS_2_. **e** CV curves of the F-SC at the different scan rates. **f** Galvanostatic charge-discharge (GCD) curves of one F-SC at different current. **g** Cycle stability for 1200 cycles for F-SC (the insert is the detail of GCD curves after long time charge-discharge). **h** CV curves of the 5 F-SCs connected in series at the different scan rates. **i** The summary plot of capacitances of 5 F-SCs connected in series versus different discharging current.

To store the converted energy efficiently, a F-SC with few-layered MoS_2_ as active material was fabricated. The X-ray diffractometry (XRD) pattern of the few-layered MoS_2_ is shown in Fig. 5a which is compared with the bulk sample. The characteristic peaks of few-layered MoS_2_ were observed at 33.69° and 59.51°, which corresponds to the (100) and (110) planes, respectively^32^. The (103) and (105) peaks were also observed, which are most interesting features in the x-ray pattern of MoS_2_^33^. Figure 5b shows the Raman spectra of few-layered MoS_2_ and bulk MoS_2_ excited by 532 nm laser lines in air ambient environment. *E*^1^_2g_ (~383 cm^−1^ for bulk MoS_2_) and *A*_1g_ (~408 cm^−1^ for bulk MoS_2_) modes are observed in both few-layered and bulk MoS_2_. The frequency of *E*^1^_2g_ peak increased obviously for few-layered MoS_2_ with decreasing layer number^34^. This is because the interlayer Van der Waals force in MoS_2_ decreases with the decrease in the number of layers^35^. Fig. 5c1 shows a typical scanning electron microscopy (SEM) image of the carbon fiber electrode coated with few-layered MoS_2_ with a uniform diameter of 400 μm. Fig. 5c2 further shows the cross-section SEM which indicates that the entire surface of the carbon fiber was covered with a layer of the few-layered MoS_2_.

Figure 5d compares the cyclic voltammetry (CV) curves of F-SC with or without few-layered MoS_2_ on carbon fiber at a scan rate of 50 mV s^−1^. In a two-electrode system, the potential window of the few-layered MoS_2_ F-SC is 1 V. Obviously, the F-SC with few-layered MoS_2_ on carbon fiber achieves a much higher capacitive current than F-SC with blank carbon fiber, indicating the few-layered MoS_2_ optimizes the capacitive performance significantly. The electrochemical characterization of the F-SC was analyzed using CV with scan rates ranging from 10 to 100 mV s^−1^ is also measured (Fig. 5e). By increasing the scan rate, the rectangular CV shape remains very well even at a high scan rate of 100 mV s^−1^, suggesting the possesses excellent rate capability. Figure 5f shows the galvanostatic charge-discharge (GCD) curves of the F-SC with linear shapes and nearly symmetric charge and discharge curves under small current (between 20 μA to 100 μA). As shown in Supplementary Fig. 8 and Supplementary Note 8, the F-SC delivered a specific capacitance (*C*_m_) of 196.4 F g^−1^ at a discharge current density of 2 A g^−1^, which is much higher than previous reports of MoS_2_ based supercapacitors at the same discharge current density^36–38^. The energy density (*E*) and average power density (*P*) was calculated to be 27.3 W h kg^−1^ and 1 kW kg^−1^, respectively (at a discharge current of 20 μA). The cyclic stability of the fabricated F-SC was examined using GCD analysis (with a constant current of 40 μA) over 1200 cycles (as shown in Fig. 5g). By comparing the *C*_m_, the capacitance retention of nearly 94% has been achieved for the F-SC which indicated the good cyclability of the device. Further, the inset of Fig. 5g shows the F-SC exbibits good long-term electrochemical stability, which is evident from the very stable charge/discharge curves. The charge curves are still quite symmetric relative to their corresponding discharge counterparts, showing no significant structural change of the electrode during the charge/discharge processes^39^.

Five F-SCs are connected in series to provides a tunable operating voltage for storing converted energy from S-TENG. Figure 5h shows the CV curves of 5 F-SCs connected in series under different scan rate ranging from 100 mV s^−1^ to 2 V s^−1^. The voltage could reach 5 V for the 5 F-SCs. The rectangular CV shape remains very well even at a high scan rate of 2 V s^−1^, indicating its excellent rate capability. The GCDs of the 5 F-SCs connected in series are also shown in Supplementary Fig. 9 and detailed discussion is in Supplementary Note 9. Figure 5i represents the plots of obtained capacitances (*C*) of the 5 F-SCs against the discharge currents of the charge-discharge profiles. The *C* of the 5 F-SC connected in series under different current can be calculated from the GCD curves according to the following equation:

4 $$C = \frac{{I\Delta t}}{{\Delta V}}$$

Where *I* is the discharge current, *∆t* is the discharge time, *∆V* is the potential window during the discharge process. The 5 F-SCs delivered the *C* of 0.416 mF at a discharge current of 20 μA while 1.096 mF at a discharge current of 200 nA.

### Application of the energy ball

**Fig. 6:**
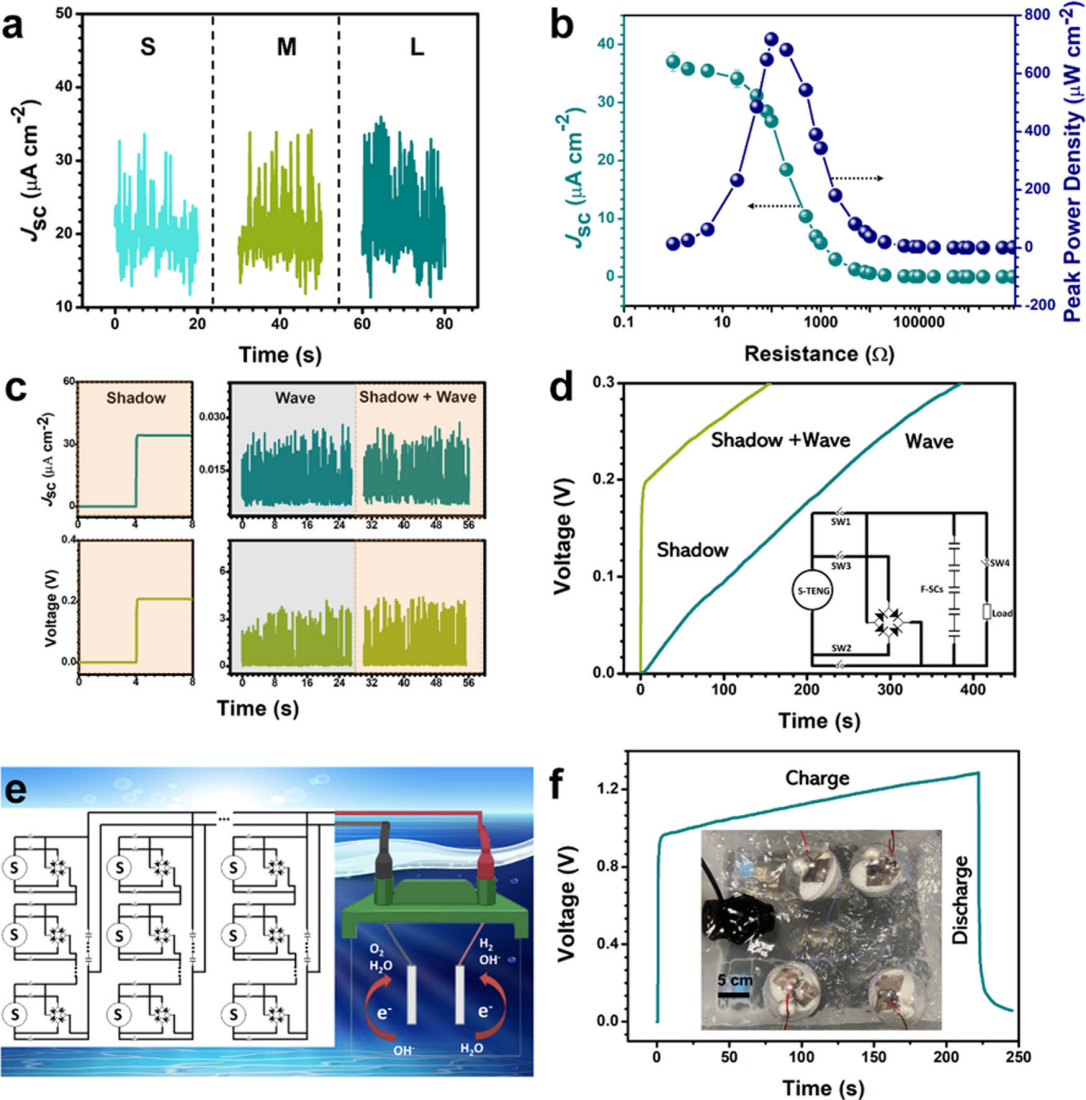
Application properties of the energy ball. **a** Intensity of wave influence on output of shadow-tribo-effect nanogenerator (S-TENG) in energy ball. **b** Short-circuit current density (*J*_sc_) and power density of S-TENG in energy ball with various loads. **c** The measured *J*_sc_ and voltage of the S-TENG in the energy ball before and after being rectified. **d** Energy ball charging curve under single illumination (Shadow), illumination mixed wave (Shadow + Wave) and single wave (Wave). Inset is electric circuit of the S-TENG based self-charging power system and load. **e** Circuit management of S-TENG based self-charging power system applies in electrolysis of seawater. **f** Four energy balls are connected in series to charge 5 fiber-supercapacitors (F-SCs) in series and then discharge the F-SCs by seawater splitting. Inset is the four energy balls working together.

To test the energy ball for energy harvesting from both wave and light, the environment of the ocean surface was simulated. We used a wave machine to create waves in the water and use a lamp to illuminate the devices. The working mechanism of S-TENG in the energy ball is mainly based on TE under a dark condition (Supplementary Fig. 10). The detailed discussion is presented in Supplementary Note 10. The image of the fabricated energy ball floating on water is also shown in Supplementary Fig. 11. An aluminum ball was chosen to be the opaque moving object that casts a shadow on the surface of the S-TENG (Supplementary Fig. 11 and Supplementary Note 11). As shown in Supplementary Movie 2, the energy ball can scavenge mechanical energy from the waves and light energy from the lamp simultaneously. The dependence of the *J*_sc_ on the wave intensity was investigated, as shown in Fig. 6a. The *J*_sc_ increases with the intensity of wave increasing from small (S), middle (M), to large (L). The stability test was carried out by continuously testing *J*_sc_ of S-TENG at a L intensity of wave and illumination by a lamp (Supplementary Fig. 12 and Supplementary Note 12). The *J*_sc_ of the S-TENG was measured for over 700 s and no observable degradation was noticed, which indicates the high stability and durability of the S-TENG. The output of energy ball under illumination at various incidence angles is also measured (Supplementary Fig. 13 and Supplementary Note 13). Figure 6b displays the resistance dependence of both *J*_sc_ and the corresponding peak power density of the S-TENG in energy ball under the illumination condition. *J*_sc_ decreases with increasing the loading resistance, while the peak power density increases in the initial stage and then decreases under the larger loading resistances. The largest peak power density of S-TENG is about 718 μW cm^−2^ at a loading resistance of 100 Ω, which mainly depends on the SE. The output current drops slowly when the S-TENG connected to a resistance of over 100 kΩ. Comparing the results shown in Fig. 4f, the impedance of SE is smaller than TE. When connecting SE and TE with quite different internal impedances in parallel, the powering efficiency cannot be improved directly. To this end, five F-SCs in series are connected with S-TENG to form a self-charging power system.

The output of the S-TENG connected with F-SCs in the self-charging power system is shown in Fig. 6c while the circuit diagram of the whole system is shown inset in Fig. 6d. The *J*_sc_ and voltage shown in left panels of Fig. 6c were generated by only SE of S-TNEG under the light illumination condition, which related to the step of closing Switch (SW) 1 and SW2. In this stage, the voltage of F-SCs increased sharply from 0 to 0.2 V in 4 s by only casting the shadow of Al ball. SW1 and SW2 were opened and SW3 was closed simultaneously, and a rectifier was connected with S-TENG to transfer current from AC to DC. The S-TENG can provide a much higher output voltage to compensate the limitation of SE after rectified when it is stimulated by waves. The rectified output before and after illuminating the energy ball were also tested. As shown in the right panel of Fig. 6c, the rectified voltage of S-TENG is about 4.1 V, which is the same under both dark and illumination conditions. This is because there is a voltage drop (0.7 V for Si diode) in the rectifier, only allowing higher voltage to pass. The output of SE will be blocked by the rectifier due to its lower voltage (0.2 V) than this barrier. In this case, the opposite currents of the SE and TE will not influence the self-charging power system that can be charged to a higher voltage and obtain wave energy. Thereby, the limitation of the impedance mismatching of S-TENG is avoided in the self-charging power system. After the F-SCs was charged, SW1, 2, 3 were opened and SW4 was closed to power the load. Under light illumination and without rectification, the F-SCs can only be charged to 0.2 V by the SE. When the energy ball was stimulated by both wave and shadow, it could be charged to a larger voltage than single SE. When the energy ball was only stimulated by wave, it took 409.4 s to charge the F-SCs to 0.3 V and the corresponding curve is shown in Fig. 6d. When both wave and shadow was used to stimulate the energy ball, the charging time was significantly shortened to 156.1 s. This result implies that the S-TENG can harvest both light energy and mechanical energy and charge the F-SCs with a shorter time by SE enhancement.

Electrolysis of seawater an environment friendly approach for production of hydrogen (H_2_) fuel. An external power supply for driving the oxidation or reduction reactions of H_2_O molecules is mandatory for electrolysis. As shown in Fig. 6e, by paralleling groups of energy balls in series, the generated and stored electricity is used for water splitting to produce H_2_. During the day or night, the energy balls could supply the energy required for water splitting continuously. A hydrogen fuel cell system collects and store generated H_2_ gas. (Supplementary Fig. 14 and Supplementary note 14). To demonstrate, a group of 4 energy balls in series was connected to split the seawater (insert in Fig. 6f). A carbon fabric and a platinum sheet with the same size of 4 cm^2^ were used as electrodes to perform the electrolysis reaction. The F-SCs in series are charged to 1.3 V using both shadow and waves, and then discharged to split the seawater (Fig. 6f). This shows the potential application of energy balls in circuit management for large area energy harvesting from ocean. By circuit management, the stored energy will be used up for seawater splitting and then the F-SCs could be re-charged. Therefore, an all-day energy conversion and storage will come true. At the same time, the circuit management could also improve the reliability of energy supply. The energy efficiency of energy ball is calculated as 0.7% under the shadow-enhanced condition (Supplementary Note 15). The peak power in ideal case is also calculated to be 0.69 MW km^−2^ (Supplementary Fig. 15). The characteristics of S-TENG based energy ball compared with other pure TENG based technologies for energy harvesting from ocean in Supplementary Table 1 and detailed discussion is presented in Supplementary Note 16. The outstanding performance of this energy ball enables capture of both wave and solar energy from the ocean surface continuously, which also makes it an attractive technology for commercialization.

## Discussion

In summary, a hybrid energy system which converts wave/solar energy to electric energy from oceans is fabricated in an energy ball. By utilizing the shadow-effect and tribo-effect, the hybrid energy system overcomes the performance degradation caused by shadows cast from moving objects in the system. A peak power density of 718 μW cm^−2^ is achieved by wave stimulation and light illuminating on the hybrid energy system. By contrast, using only tribo-effect outputs a power density of 0.28 μW cm^−2^ which verifies the shadow-effect enhanced working principle of the entire system. The self-charging power system, which is developed by integrating the few-layered MoS_2_ F-SCs with the hybrid energy system, exbibits good stability and can store the converted energy reliability. Comparing with only harvesting wave energy, both wave and shadow simulating the energy ball shortens 253.3 s to charge the F-SCs to the same voltage (0.3 V). This work provides new insights on high-performance self-charging power system for wave and solar energy harvesting and storage. In addition, the energy ball based on the self-charging power system has potential applications in circuit management such as electrolysis of seawater for large area energy harvesting from ocean. The shadow-enhanced self-charging power system also offers new avenues for design/optimization of next-generation hybrid energy systems towards blue energy harvesting.

## Method

### Fabrication of the S-TENG and energy ball

A cleaned n-type Si wafer (15 cm^2^) with thickness of 530 μm was used to be a substrate of S-TENG. 15 nm Au was coated on the n-type Si by thermal evaporator. A gap in the Au film was added by removing its middle part by 1 cm. After the Au film in the middle being removed, PDMS film was fabricated on the Au film by a doctor-blade technique^40^, followed by thermal annealling at 60 °C for 2 h. Then the S-TENG was fabricated. For fabrication of the energy ball, the as-fabricated S-TENG was fixed in a large transparent plastic sphere with an 8 cm diameter. A little Al ball (with a diameter of 2.5 cm) was put on the S-TENG to be a moving object for casting shadow on S-TENG. A counterweight is arranged in the bottom of energy ball, which provides balance and stability of the energy ball in water. The F-SCs were embedded under the S-TENG. The S-TENG and the F-SCs were connected by a small full-wave rectifier.

### Fabrication of F-SCs

A carbon fiber was pretreated in acetone, ethanol and deionized water, respectively, for 15 min under ultrasonication and then was cut into a bundle with 1 cm for later use. A solution was prepared by add 0.04 g few-layered MoS_2_ (Xian Fen LTD.) into 5 mL ethanol. The coated carbon fiber was prepared by soaking carbon fiber in the solution for 24 h, removed, and then baked at 150°C. Repeat the soak-bake process for several times. H_2_SO_4_ (Sulfuric acid, 6 g) and PVA (poly (vinyl alcohol), 6 g) were added into 60 mL of deionized water, which were heated to 85 °C under stirring until the mixture became clear. Then, the H_2_SO_4_/PVA gel electrolyte was prepared. An F-SC was assembled by separating two as-fabricated electrodes with the H_2_SO_4_/PVA gel electrolyte: Soak two electrodes into H_2_SO_4_/PVA gel electrolyte and then assemble them. Then, five F-SCs were assembled following the same process.

### Characterizations and measurements

The short-circuit current density of the S-TENG were measured by a Stanford low-noise current preamplifier (Model SR570). A digital oscilloscope (DS4052, RIGOL) was used to test the electric output voltage of the S-TENG. The resistance of the oscilloscope is 100 MΩ. The transferred charge density and the open-circuit voltage of S-TENG were measured by an electrometer (Keithley 6514). The AFM and KPFM measurements were performed using Dimension Icon (Bruker Nano Surfaces). Amplitude modulation KPFM was used to obtain a high signal-to-noise ratio as opposed to that of frequency modulation. All KPFM measurements were performed in dual pass mode. Work function measurements were performed with a Pt/Ir tip (5.5 eV) on the Au/n-Si surface. The current-voltage curves were measured by Keithley 2450. In order to accurately study the dependence of output performance of S-TENG, a force gauge testing system (Mecmesin, MultiTest 2.5-i) is used for the test. The force gauge testing system is mainly composed of a fixed platform and a moving stage. Polyurethane foam is pasted on the surface of moving stage to contact with the surface of S-TENG. The contact area is about 7.5 cm^2^. Distance between moving stage and fixed platform, force magnitude and moving speed of the moving stage can be pre-set. The moving stage moves towards the S-TENG in the pre-set speed until force reaches 0.1 N. Then the moving stage moves apart from the S-TENG with the pre-set speed to the original position and begins next cycle. CV, GCD and open-circuit voltage of F-SCs were investigated on electrochemical analyzer system (CHI660D) at room temperature. The few-layered MoS_2_ and bulk MoS_2_ were characterized by XRD (X’Pert Pro diffractometer with a Cu Kα radiation, λ = 0.154 nm). The Raman measurements with the excitation laser line of 532 nm was performed using a WITEC alpha300 R Confocal Raman system in air ambient environment. The structure and morphology of the electrode of the F-SC was measured by the scanning electron microscopy (SEM, Zeiss Spura 55). The wave machine used in this work is Jebao (OW-10). The chemicals (27.21 g Sodium chloride, 3.81 g Magnesium chloride, 1.66 g Magnesium sulfate, 1.404 g Calcium sulfate, 0.577 g Potassium sulfate) were dissolved in 1 L distilled water to simulate electrolysis of seawater.

## Supplementary information

Supplementary Information

Peer Review File

Description of Additional Supplementary Files

Supplementary Movie 1

Supplementary Movie 2

## Data Availability

The data that support the findings of this study will be made available from the corresponding authors upon reasonable request.

## Code availability

The codes that support the findings of this study are available from the corresponding author upon reasonable request.
