# Supplementary material for: Shadow enhanced self-charging power system for wave and solar energy harvesting from the ocean
Source: Nat Commun. 2021 Jan 27;12:616. doi: 10.1038/s41467-021-20919-9 (PMC7841174; doi:10.1038/s41467-021-20919-9)
Supplement: Supplementary file 1 — Supplementary Information [file 41467_2021_20919_MOESM1_ESM.pdf]

Supplementary Information

Shadow Enhanced Self-Charging Power System for Wave and Solar Energy Harvesting from the Ocean

*Qian Zhang[1, a], Qijie Liang[2, a], Dilip Krishna Nandakumar[1], Hao Qu[1], Qiongfeng Shi[3], Fuad Indra Alzakia[1], Darrell Jun Jie Tay[1], Lin Yang[1], Xueping Zhang[1], Lakshmi Suresh[1], Chengkuo Lee[3], Andrew Thye Shen Wee[2], and Swee Ching Tan [1,] **

[1] Department of Materials Science and Engineering, National University of Singapore, 9 Engineering Drive 1, Singapore 117574, Singapore.

[2] Department of Physics, National University of Singapore, 2 Science Drive 3, Singapore 117551, Singapore.

[3] Department of Electrical and Computer Engineering, National University of Singapore, 4 Engineering Drive 3, Singapore 117576, Singapore.

* Corresponding author. e-mail: msetansc@nus.edu.sg

[a] These authors contributed equally: Qian Zhang, Qijie Liang

Supplementary Note 1. Photograph of the fabricated device

Supplementary Figure 1 is the photograph of the as fabricated energy ball with a diameter of 8 cm. A counterweight is arranged in the bottom of energy ball, which provides balance and stability of the energy ball in water.

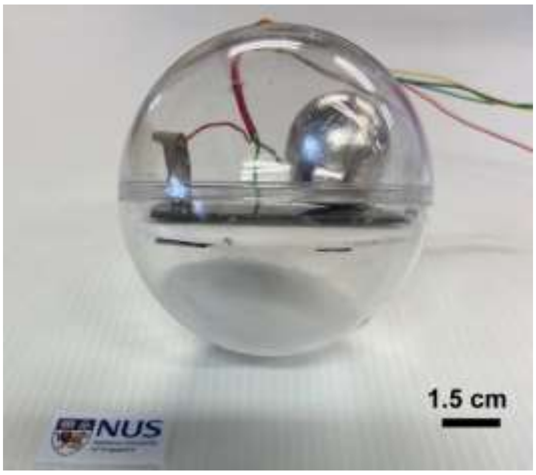

Supplementary Figure 1 | Photograph of the as-fabricated energy ball.

Supplementary Note 2. The current-voltage characteristics of Au film half on n-Si and pure n-Si

To measure and verify the Schottky contact between Au and n-Si, 15 nm Au was sputtering on half of the n-Si (Supplementary Figure 2a). The current-voltage characteristic was used to detect this half-Au on n-Si system in light or dark conditions, respectively. For comparison, n-Si also be characterized current-voltage curve. As shown in Supplementary Figure 2b, under forward bias and light condition, the current of half-Au on n-Si rises very rapidly while under reverse bias, there is a small leakage current. This typical Schottky barrier property also exists even the half-Au on n-Si system in a dark condition, which verified there is a Schottky contact between 15 nm Au film and n-Si. In addition, The resistance of n-Si is much larger than Au film on n-Si.

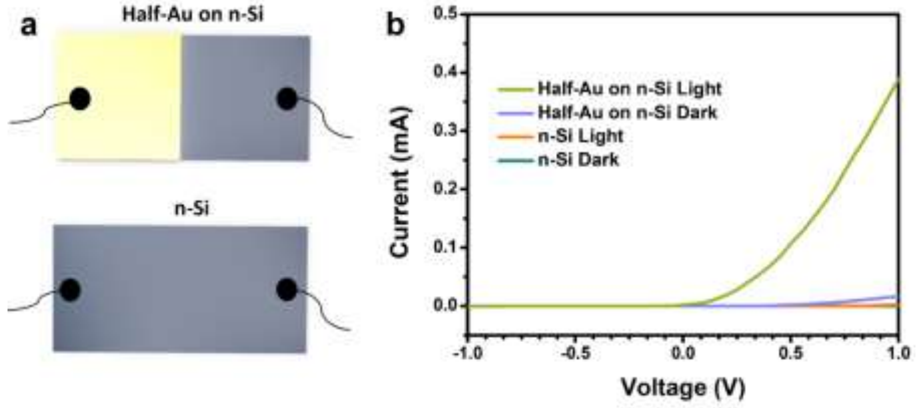

Supplementary Figure 2 | a Schematics of fabricated half-Au on n-Si and n-Si. **b** The measured current-voltage curve of half-Au on n-Si and n-Si.

Supplementary Note 3. The atomic force microscopy (AFM) and kelvin probe force microscopy (KPFM) image analysis of Au/n-Si system

Surface potential of Au/n-Si with 15 nm Au film in dark is shown in Supplementary Figure 3a while under illumination is shown in Supplementary Figure 3b. The average surface potential is about 5.26 eV and 5.39 eV, respectively. A fine-grained structure in the Au film deposition on n-Si is shown in Supplementary Figure 3c and Supplementary Figure 3d.

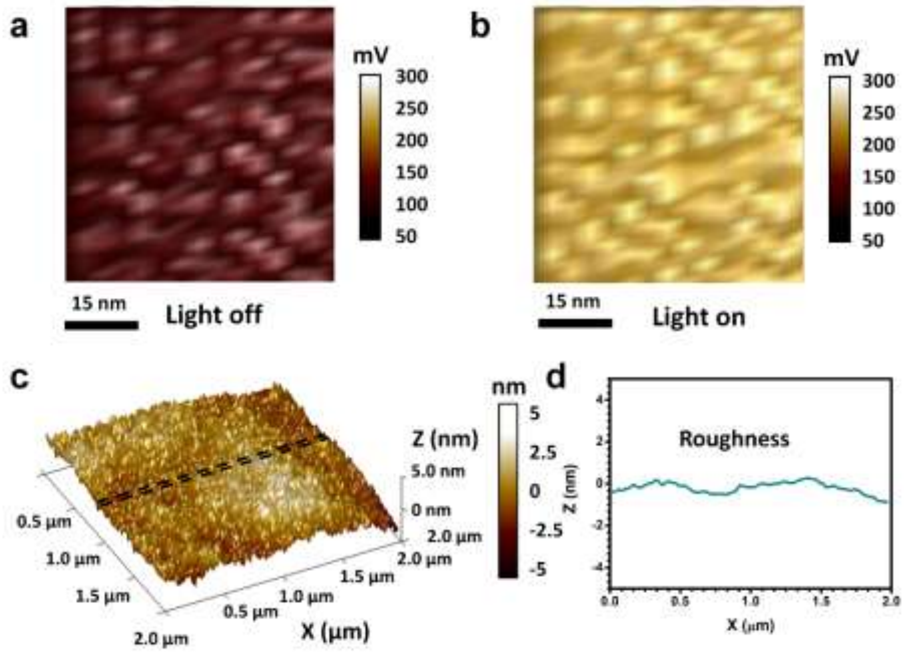

Supplementary Figure 3 | Surface potential maps of Au/n-Si with 15 nm Au film under **a** light on and **b** light off. **c** Atomic force microscopy image of the Au film on n-Si. **d** The surface roughness data obtained from cross section of black dashed line in **c**.

Supplementary Note 4. Resistance of Au film on Au/ n-Si system

A Extech EX503 (Industrial Digital Multimeter, 1000V, 10A, 4000 Count) was used to measure the resistance of Au film on n-Si. The width of the film is 3 cm and the space between the two wiring points is about 4 cm. The electrical resistance of Au film with 15 nm, 60 nm, 240 nm on n-Si are shown in Supplementary Figure 4, which decreased from 39.1 Ω, 18.2 Ω to 3.4 Ω.

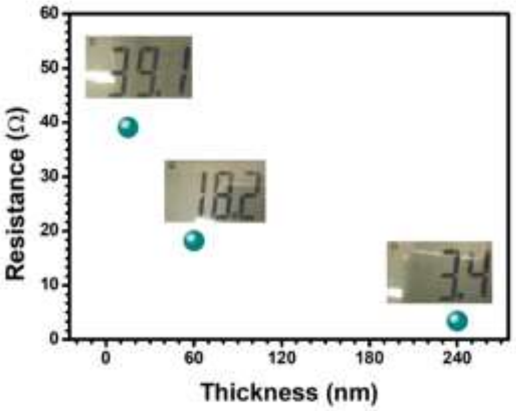

Supplementary Figure 4 | Electrical resistance of Au film versus thickness of Au film.

Supplementary Note 5. The output of transparent water droplets touched on PDMS/Au/n-Si

The working mechanism of the PDMS/Au/n-Si (without a gap) can be illustrated as a single-electrode TENG [1]. When a water-drop carrying positive charges comes into contact with the PDMS layer (Supplementary Figure 5a(ii)), a positive electric potential difference will be formed between the charged water-drop and the ground. This causes the electrons to flow from the ground to the Au electrode and generate an instantaneously opposite potential to balance the electric field generated due to this electron flow, until an equilibrium is attained (Supplementary Figure 5a (iii)). Once the charged water-drop leaves the PDMS layer, a negative electric potential difference will be formed, forcing the electrons to flow from the Au electrode to the ground (iv), until another equilibrium is attained (Supplementary Figure 5a (i)). The influence of the tilt angle (θ) of water-drop on output voltage (V_{op}) was measured, as plotted in Supplementary Figure 5b. It is clearly shown that V_{op} increased from 2 V to 7 V when the θ increased from 30° to 60°. With a further increase in θ, V_{op} decreased to 1.5 V.

When θ is large, the effective contact surface area between water drops and PDMS/Au/n-Si is small. The water drops cannot flow away quickly as θ is small. Thus, the output is maximum when the θ is around 60° owing to the trade-off between surface contact area and the rate of sliding of the water drops [2]. Supplementary Figure 5c shows short-circuit current density (J_{sc}) of the PDMS/Au/n-Si at different dripping heights (h) from 5, 10 to 15 cm. With increasing water dripping height, J_{sc} increases from 9.2, 11.8, 13.4 to 19.1 nA cm^{-2}. The power density (P_s) of the PDMS/Au/n-Si can be calculated as:

$$P_s = J_{sc} V_{op} \qquad \text{(Supplementary Equation 1)}$$

The P_s from PDMS/Au/n-Si (θ=60°, h=15 cm) is 0.14 μW cm^{-2}, proves that the PDMS covers on Au/n-Si system could generate power by a tribo-effect. There was no significant change in J_{sc} for harvesting the water-drop energy under illumination and dark (Supplementary Figure 5d). The open-circuit voltage (V_{oc}) and charge density ($\Delta\sigma_{sc}$) were also measured (Supplementary Figure 5e), which didn't have significant changes too. This shows that the effect of light illumination on the output performance of PDMS/Au/n-Si is negligible. In another words, the illumination and transparency of the water drop does not induce an output increasing of PDMS/Au/n-Si.

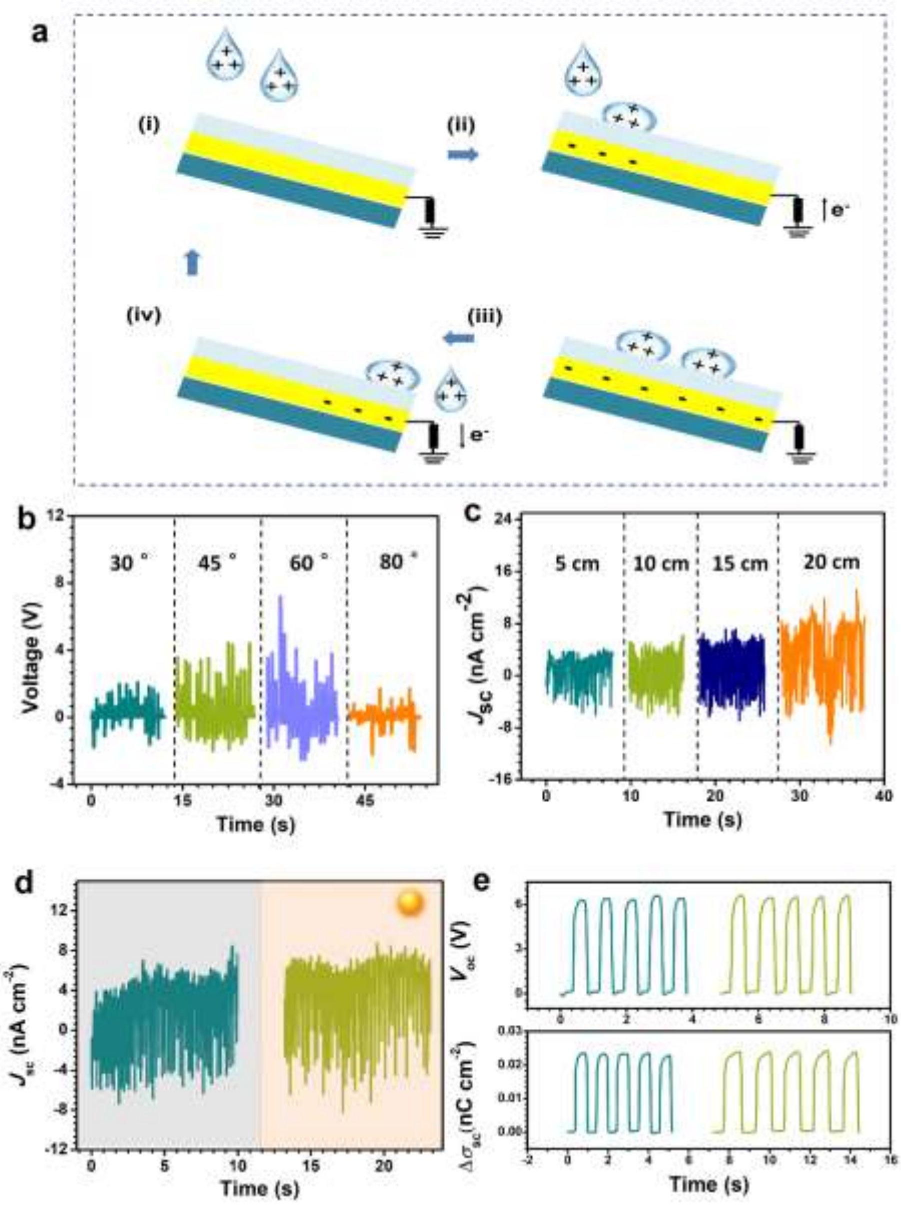

Supplementary Figure 5 | Working principle and output of the PDMS/Au/n-Si harvests water-drop energy. **a** Step-by-step illustration showing the working principle of the PDMS/Au/n-Si by harvesting energy from water-drop. **b** Influence of the tilt angle of water-drop on the output voltage. **c** Influence of the falling height of water-drop on the short-circuit current density (J_{sc}). **d** J_{sc} of the PDMS/Au/n-Si in dark and illumination by water-drop falling on it. **e** The open-circuit voltage (V_{oc}) and charge density ($\Delta\sigma_{sc}$) of PDMS/Au/n-Si for harvesting the water drop energy under dark and illumination conditions.

Supplementary Note 6. Transmittance of glass and black paper on glass

As shown in Supplementary Figure 6, the average transmittance of glass and black paper on glass is 80% and 0%, respectively. The black paper was attached on the transparent glass to make the glass opaque.

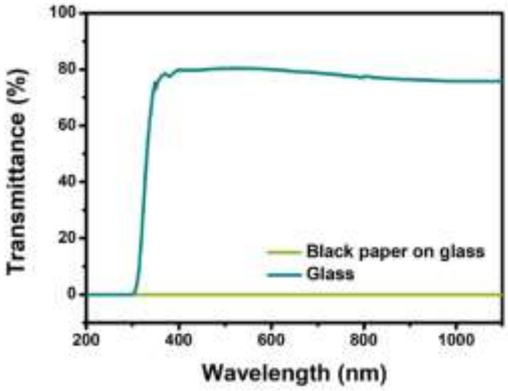

Supplementary Figure 6 | Transmittance of glass with or without black paper on it.

Supplementary Note 7. Magnified detailed waveforms of shadow-tribo-effect nanogenerator (S-TENG) in dark and illumination by moving stage up and down

As shown in Supplementary Figure 7, when the shadow of stage casting on S-TENG as stage moving down under illumination, the J_{sc} increased. The Supplementary Figure 7a shows the magnified detailed waveforms of S-TENG under dark condition, which is a typical tribo-effect output with AC signal. When moving stage downwards above half of S-TENG under illumination, the J_{sc} increased to about 34 μA cm^{-2} (Supplementary Figure 7b). As moving stage upwards, the J_{sc} decreased. The J_{sc} doesn't decrease to 0 μA cm^{-2} because there's an illumination contrast already existing which was caused by light source with an incidence angle on S-TENG.

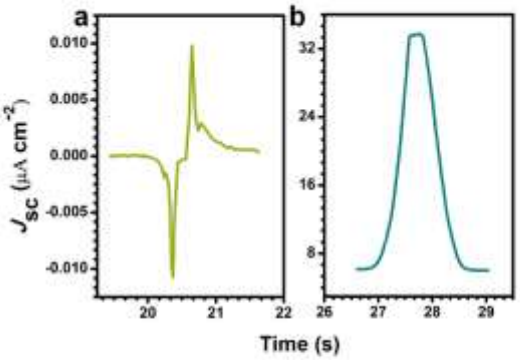

Supplementary Figure 7 | The magnified detailed waveforms for the outputs of half shadow-tribo-effect nanogenerator **a** under dark and **b** illumination conditions.

Supplementary Note 8. Specific capacitance, energy density and power density of F-SC

Supplementary Figure 8a represents the plot of obtained specific capacitance of the fiber-supercapacitor (F-SC) against the discharge currents of the charge-discharge profiles. The specific capacitance (C_m) of the F-SC can be calculated from the curves according to the following equation:

$$C_m = \frac{I\Delta t}{m\Delta V} \qquad \text{(Supplementary Equation 2)}$$

Where I is the discharge current, Δt is the discharge time, ΔV is the potential window during the discharge process, and m is the load mass of the few-layered MoS_2. The F-SC delivered a C_m of 196.4 F g^{-1} at a discharge current density of 2 A g^{-1}. The C_m of F-SC was maintained at 50 F g^{-1} even when the current density was increased to 10 A g^{-1}. The energy density (E) and average power density (P) of the F-SC can be calculated using the following equations:

$$E = \frac{0.5 C_m \Delta V^2}{3600} \qquad \text{(Supplementary Equation 3)}$$

$$P = \frac{3600E}{\Delta t} \qquad \text{(Supplementary Equation 4)}$$

Supplementary Figure 8b shows the Ragone plots (E vs. P) of the F-SC. It shows that the fabricated F-SC possesses an E of about 27.3 W h kg^{-1} (at a discharge current of 20 μA) with a corresponding P of 1 kW kg^{-1}. The E decreases from 27.3 to 6.9 W h g^{-1} and the P increases from 1 to 5 kW kg^{-1} as a result of increase in discharge current from 20 μA to 100 μA.

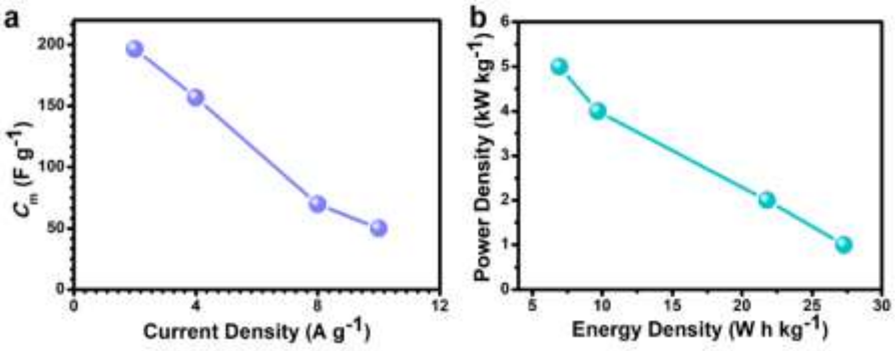

Supplementary Figure 8 | a The summary plot of specific capacitance (C_m) versus different discharging current density. **b** Ragone plot of the fiber-supercapacitor.

Supplementary Note 9. galvanostatic charge-discharge (GCD) cureves of 5 F-SCs connected in series under different charging-discharging current

Supplementary Figure 9a and 9b shows the GCD curves of the 5 F-SCs connected in series under current ranging from 1 μA to 20 μA and 200 nA to 800 nA. The voltages of these GCD curves vary from 0 to 5 V. It can be seen that the 5 F-SCs connected in series could be used to efficiently store electric energy even with small charging current (200 nA). Supplementary Figure 9c shows the details of the galvanostatic charging curve of 5 F-SCs connected in series at 200 nA, 400 nA, and 800 nA from 0 V to 0.3 V.

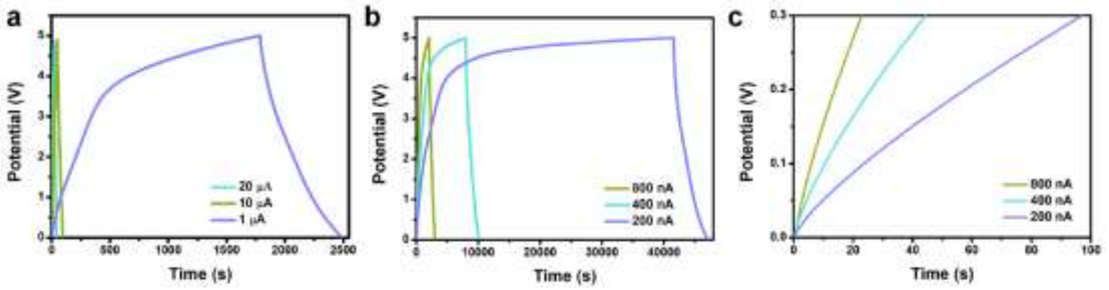

Supplementary Figure 9 | Galvanostatic charge-discharge curves of 5 fiber-supercapacitors (F-SCs) connected in series at current of **a** 1 μA, 10 μA, and 20 μA, **b** 200 nA, 400 nA, and 800 nA. **c** Details of the galvanostatic charging curve of 5 F-SCs connected in series at 200 nA, 400 nA, and 800 nA from 0 V to 0.3 V.

Supplementary Note 10. Working mechanism of S-TENG in the energy ball

The equivalent circuit of the S-TENG in energy ball with an ammeter is illustrated in Supplementary Figure 10a. As being depicted in Fig. 3a, the working mechanism of S-TENG in the energy ball with an Al ball on it is mainly based on TE under a dark condition. The only difference between using moving stage and Al ball as the opaque object is that the moving direction of the opaque object changes from vertical to horizontal direction (Supplementary Figure 10b). The optical image of the energy ball under four related stages in Supplementary Figure 10a and b is also shown in Supplementary Figure 10c.

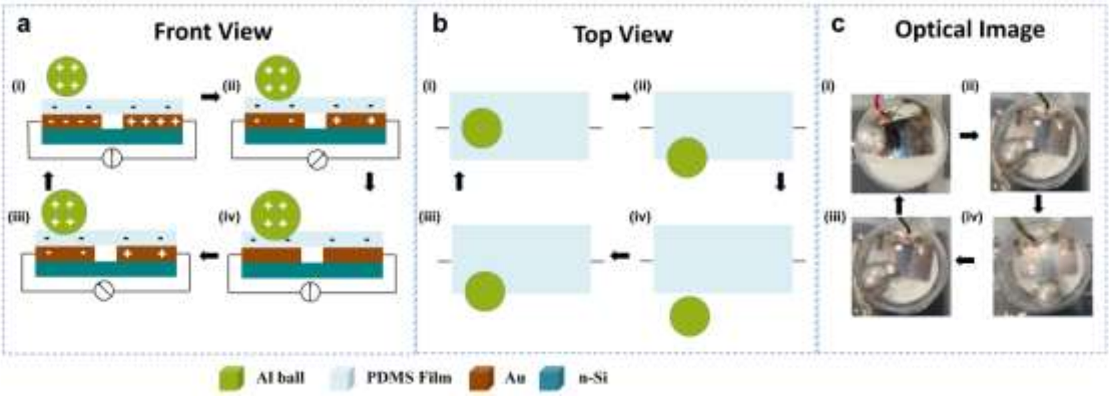

Supplementary Figure 10 | a Front view and **b** Top view of the working principle of the shadow-tribo-effect nanogenerator in the energy ball. **c** Optical images of the energy ball under four related stages shown in a and b.

Supplementary Note 11. Comparison of different materials balls on S-TENG

The triboelectric output of S-TENG with an aluminum (Al) ball and a steel ball on S-TENG respectively were tested in dark. As shown in Supplementary Figure 11, there is an obvious output change of S-TENG with these two different material balls on it. That is because the Al is a better tribo-positive material than steel, which is further away from PDMS in triboelectric series. [3] Thus the Al ball was chosen to be moving object on the S-TENG.

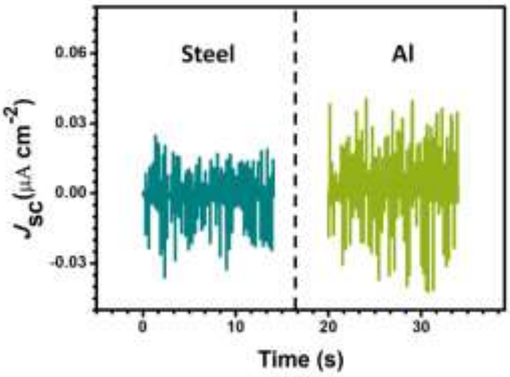

Supplementary Figure 11 | Triboelectric output of shadow-tribo-effect nanogenerator with a steel ball or Al ball on it.

Supplementary Note 12. Stability and Duribility of S-TENG

The output of S-TENG under the influence of continuous waves and light illumination was tested. As shown in Supplementary Figure 12, there is slightly changes of S-TENG output causing by changes in the intensity of the waves during more than 700 s. The

output of S-TENG under continuously waving and illuminating stimulation slightly changed because of ups and downs of waves. The S-TENG exhibited good stability and durability, which makes it reliable for potential application.

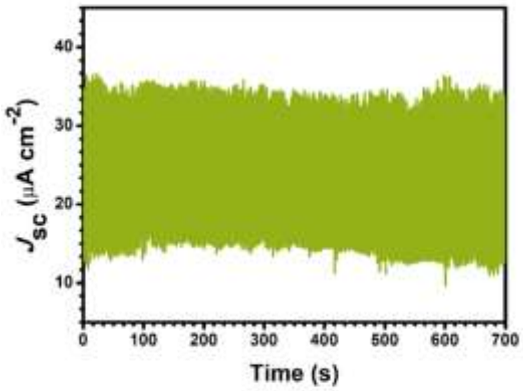

Supplementary Figure 12 | The stability test of the shadow-tribo-effect nanogenerator (S-TENG), the measured results of J_{sc} of the S-TENG continuously operating for more than 700 s.

Supplementary Note 13. Output of energy ball under illumination at various incidence angles

To simulate a nature environment, in which the shadows of the sunlight moves as time advances, a moving light source to different relative positions was used to change the incidence angle (α). As shown in Supplementary Figure 13, the α was changed from 0°, 45°, 90°, 135°, to 180°. When α=90°, the output reaches highest. The reason is when the α is 0°, 45°, 135°, or 180°, the shadows area is larger than that of α=90°. In addition, work-function non-uniformity on the Au/n-Si system when α is not 90° also influences the output of energy ball.

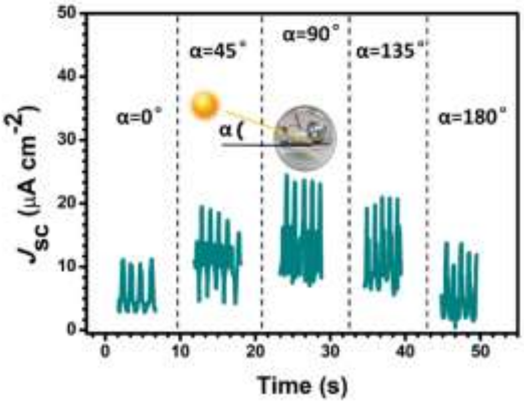

Supplementary Figure 13 | Output performance of energy ball at various incidence angles.

Supplementary Note 14. Process scheme of fuel cell production based on seawater electrolysis

One of the possible ways to deploy the energy generated by the S-TENG is to utilize the electrical energy output of the S-TENG to electrolyse sea water to produce Hydrogen gas. A fuel cell as shown in Supplementary Figure 14 could be used to capture and store the hydrogen gas and supply it based on demand. During the day, the energy balls convert both solar and wave energy in to electricity and store in F-SCs. The stored electricity drives the direct seawater electrolyzer and produces hydrogen, which meets the power demand of city. At night, in the absence of solar and SE, the electrical power of seawater electrolysis can be provided by the waves.

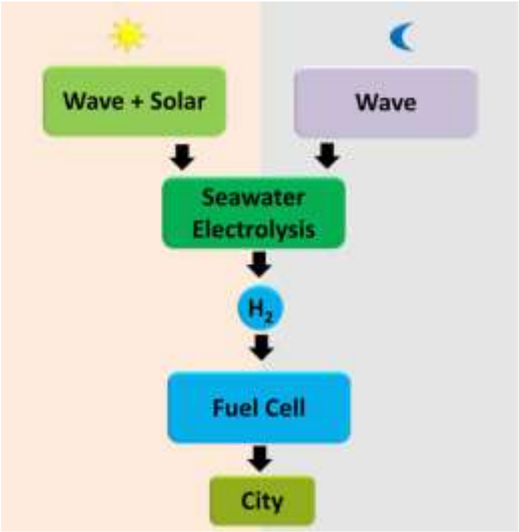

Supplementary Figure 14 | Process scheme of fuel cell production based on seawater electrolysis.

Supplementary Note 15. The calculation of energy efficiency and peak power of S-TENG based energy ball

The energy conversion efficiency (η) of S-TENG in one energy ball can be calculated by:

$$\eta = \frac{P_{\text{peak}}}{P_{\text{light}} + P_{\text{wave}}} \times 100\% \qquad \text{(Supplementary Equation 5)}$$

Where P_{peak} is maximum peak power of S-TENG in one energy ball, P_{light} is calculated to be 1500 mW. P_{wave} is 21.4 mW, which calculated by dividing the power of wave machine (12 W) by amount of water (15 L) and multiply volume of energy ball. As shown in Fig. 6b, the P_{peak} of an energy ball is 10.8 mW. The η of an energy ball is calculated to be 0.7%. As shown in insert of Fig. 6f, four energy balls ocupy an area of

about 625 cm^2 in a simulated ocean environment and the increase of unit quantity could increase output. An energy ball could be calculated to occupy 156.25 cm^2 in Supplementary Figure 15. In an ideal case, the peak power density of S-TENG based energy balls is 0.69 MW km^{-2}, which is calculated by multiplying the P_{peak} of one energy ball by amount of energy balls per square kilometer. [4]

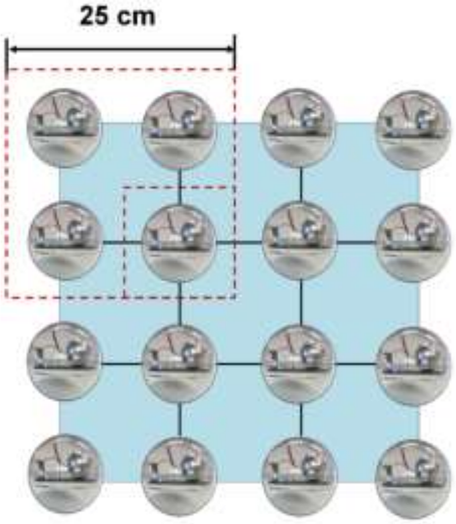

Supplementary Figure 15 | Shadow-tribo-effect nanogenerator based energy balls arrays for calculating the occupied area of an energy ball.

Supplementary Note 16. Comparison between different technologies for energy harvesting from ocean

Supplementary Table 1 is the comparison between different technologies for energy harvesting from ocean which all based on the TENG. Comparing with the other technologies, the S-TENG in this work have the largest current output or peak power per one unit due to the shadow-effect, respectively. In addition, as other blue energy technologies, the S-TENG doesn't need complex design intricate engineering or expensive raw materials. The fabrication of the S-TENG can be done at extremely low cost and does not involve any harmful chemicals nor intricate synthesis protocols. These advantages make the S-TENG an attractive device for commercialization.

Supplementary Table 1 | Key characteristics of TENG based technologies for energy harvesting from ocean

	Structure<br>Size	Output current per unit (μA)	Peak power per unit /power density
S-TENG this work	PDMS doped on Metal/n-Si system Steel ball/	555	10.8 mW/<br>7.18 W m^{-2}/<br>0.69 MW km^{-2}

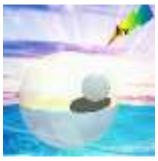	Diameter of energy ball is 8 cm		
RF-TENG [5]<br>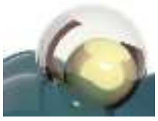	Nylon ball<br>Kapton-Al/<br>Diameter of whole device is 6 cm	1	10 mW
WS-TENG [6]<br>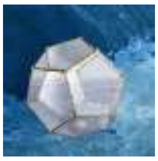	Cu-Kapton-Cu<br>FEP-Cu/<br>Circumradius of the dodecahedron r calculated to be 11.9 cm	150	0.64 MW km^{-2}
OS-TENG [7]<br>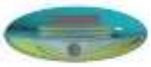	FEP-PET-Cu<br>PLA<br>Steel ball/<br>Major diameter of whole device is 12.2 cm	76	475 μW
T-TENG [8]<br>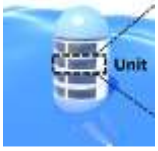 	PTFE ball-Nylon film/<br>Metal electrode<br>Diameter of 1 unit of tower is 7 cm	1.3	1.03 W m^{-3}
TS-TENG [4]<br>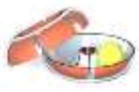	Nylon ball<br>Photopolymer-Cu/<br>Major and minor diameter of whole device is 7 cm and 5 cm	0.29	0.21 W m^{-2}
SS-TENG [9]<br>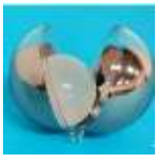	Cu<br>Silicone Rubber-liquid/<br>Diameter of the whole device is 7 cm	0.8	1.8 mW
Spherical TENG [10]<br>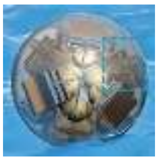	FEP-Cu-foam<br>Copper ball<br>Spring/<br>Diameter of the whole device is 15 cm	80	8.5 mW

Supplementary References

1. Xu, W. et al. A droplet-based electricity generator with high instantaneous power density, *Nature*, **578**, 392-396 (2020).

2. Zhang, Q.et al. An amphiphobic hydraulic triboelectric nanogenerator for a self-cleaning and self-charging power system. *Adv. Funct. Mater.*, 1803117 (2018).

3 Wang, Z. L. Triboelectric nanogenerators as new energy technology for self-powered systems and as active mechanical and chemical sensors, *ACS Nano*, **7**, 9533–9557 (2013).

4. Liu, W. et al. Torus structured triboelectric nanogenerator array for water wave energy harvesting, *Nano Energy*, **58**, 499-507, (2019).

5. Wang, X. et al. Triboelectric nanogenerator based on fully enclosed rolling spherical structure for harvesting low-frequency water wave energy, *Adv. Energy Mater*. **5**, 1501467, (2015).

6. Zhang, L. M. et al. Multilayer wavy-structured robust triboelectric nanogenerator for harvesting water wave energy, *Nano Energy* **22**, 87-94, (2016).

7. Liu, G., Guo, H., Xu, S., Hu, C., Wang, Z. L. Oblate spheroidal triboelectric nanogenerator for all-weather blue energy harvesting, *Adv. Energy Mater.* **9**, 1900801, (2019).

8. Xu, M. et al. High power density tower-like triboelectric nanogenerator for harvesting arbitrary directional water wave energy, *ACS Nano*, **13**, 1932-1939, (2019).

9. Cheng, P. et al. Largely enhanced triboelectric nanogenerator for efficient harvesting of water wave energy by soft contacted structure, *Nano Energy* **57**, 432-439, (2019).

10. Liang, X. et al. Spherical triboelectric nanogenerator integrated with power management module for harvesting multidirectional water wave energy, *Energy Environ. Sci*, **13**, 277, (2020).
