# Supplementary material for: Shadow enhanced self-charging power system for wave and solar energy harvesting from the ocean
Source: Nat Commun. 2021 Jan 27;12:616. doi: 10.1038/s41467-021-20919-9 (PMC7841174; doi:10.1038/s41467-021-20919-9)
Supplement: Supplementary file 3 — Description of Additional Supplementary Files [file 41467_2021_20919_MOESM3_ESM.pdf]

Description of Additional Supplementary Files

Supplementary Movie 1: Depicts the J_{sc} measurement carried out by moving the stage of a force gauge towards and away from the S-TENG under a lamp as a light source.

Supplementary Movie 2: Shows the energy ball scavenging mechanical energy from the waves and light energy from the lamp simultaneously.
