# Supplementary material for: Shadow enhanced self-charging power system for wave and solar energy harvesting from the ocean
Source: Nat Commun. 2021 Jan 27;12:616. doi: 10.1038/s41467-021-20919-9 (PMC7841174; doi:10.1038/s41467-021-20919-9)
Supplement: Supplementary file 2 — Peer Review File [file 41467_2021_20919_MOESM2_ESM.pdf]

Reviewer #1 (Remarks to the Author):

The manuscript demonstrates a device that can simultaneously harvest wave energy and optical energy in ocean, based on a triboelectric nanogenerator and a shadow-effect optical energy harvesting device. A self-charging power system was fabricated by integrating the energy harvester with a fiber-supercapacitor. The novelty is not quite enough considering that the shadow-effect device has been reported by the authors in their previous papers (DOI: 10.1039/d0ee00825g). Meanwhile, the coupling of the TENG and the shadow-effect device seems not quite obvious, and the performance is not improved enough compared with previously reported high-performance TENG devices. Detailed comments are as follows:
(1) Why a 15 nm Au layer is adopted should be clearly discussed. Is there any thickness effect? The transmittance and the resistivity should also be characterized.
(2) The mechanism shown in Figure 2c is not correct. The figure shows that two sides of a single layer of Au film is connected to a load. In such a circuit, there should be no current flowing from the load, and charges will balance the potential by moving directly in the Au layer. Two exceptions are: the Au layer also has a high resistivity due to the thin thickness, or the external circuit is short circuited. In such cases, there will be partial current flowing from the external circuit. Please check the figure and experiment setup.
(3) Similar situation as the above exists for Figure 2d. Please clearly explain why the current does not flow directly in the Au layer, but from the external load. The mechanism of the shade effect should be clearly provided. If the phenomenon is right, it seems that the shadow effect is due to the diffusion of excessive electrons from the light side to the dark side, just like a piece of metal with different temperatures.
(4) The TENG and the shadow effect seem not quite coupled. So clear investigation on each effect is necessary. For the shadow effect, more characterization without the PDMS is necessary.
(5) The experiment condition should be precisely controlled for characterization. Please don't use hand as the major method, which cannot be well controlled.
(6) The quality of data should be improved. The peaks of signals are not quite stable. Meanwhile, magnified detailed waveforms for the output should be provided.
(7) The results in Figure 2a is quite unclear. How are such data measured by the SKPM? The dimensions are also missing.
(8) The coupling of the TENG and the shadow effect seems not quit strong here. They seem working relatively independently. Meanwhile, the electron flow directions seem opposite under the same operation (contact or separation) for the two mechanisms according to Figure 2.
(9) Is the output current of the shadow effect a continuous one or a pulse one? From Figure 2b, it is continuous, but from other data, it seems a pulse one. If it is a continuous one, why it is related with frequency as in Figure 3c? Meanwhile, for a continuous current, the charging rate under illumination should be much higher than that shown in Figure 6f.
(10) The droplet experiments seems not quite relevant to other parts of the manuscript.
(11) The tidal energy and the wave energy are not the same thing. Please do not confuse.

(12) The references cited are a bit arbitrary. Please try to cite some representative references.
(13) Please provide the photo of the fabricated device.
(14) In the figures for illustrating the mechanism, the charge conservation should be satisfied.
(15) The calculation of power density 25.3 MW km-2 is unacceptable. The estimation should be based on the experimental output of the device and the occupied area of the device.
(16) Some sentences are not correct, such as "When two objects with different charges are brought together".
(17) "With light illumination, owing to the synergistic coupling between triboelectric-effect and shadow-effect, more electrons are generated and injected into the surface of the metal thereby enhancing the current density". The sentence seems not supported by the data and discussions.
(18) In nature environment, the shadow of the sunlight moves as time advances, how can the device precisely positioned to enhance the ouput in a practical situation?

Reviewer #2 (Remarks to the Author):

In this paper, a self-charging power system based on triboelectric-photovoltaics by integrating a shadow-tribo-effect nanogenerator (S-TENG) and a fiber-supercapacitor is presented, which is a method to harvest and store the tidal and solar energy. And the S-TENG is fabricated as an energy ball, generating a 25.3 MW km-2 typical output. However, the paper should make some modifications and further improvement, and its acceptance could be considered after major revision.

1. Please describe the substantive difference between the shadow-effect energy generator (SEG) and a shadow-effect enhanced triboelectric nanogenerator (S-TENG). It seems that a self-charging power system is only perfection and improvement of previous work and there is no new mechanism found in this work.

2. The named "a shadow-effect enhanced triboelectric nanogenerator (S-TENG)" is not appropriate, which is a combination of shadow-effect energy generator (SEG) and single-electrode mode triboelectric nanogenerator. In addition, the SEG based on the ubiquitous optical effect that is a photovoltaic device is the main output source of S-TENG. In terms of the comparison between different technologies for energy harvesting from ocean which all based on the TENG, I have some confusion about its rationality and please give a reasonable explanation.

3. "A small steel ball which is used as the opaque friction object on the S-TENG." is shown in the paper. Please explain why a steel ball is applied instead of using other materials, such as copper, which is a better tribo-positive material.

4. Authors depict "The working mechanism of the output 2 mode of S-TENG can be illustrated as a water-drop TENG" in this paper. The water-drop TENG is seen in characterizations of the charge generation and transfer that occur at solid-liquid or liquid–liquid interfaces. However, the S-TENG uses the characteristics of the charge generation and transfer that occur at solid-solid interface. I don't think the author's analogy is accurate, please make corresponding amendments.

5. Figure 3c show "Influence of the fingers up and down frequency on the Jsc under dark and illumination conditions". Please explain how authors ensure that the force exerted by fingers on the S-TENG is stable, which has an impact on the output performance of TENG. I recommend that authors conduct a series of experiments utilizing rotors.

6. The author studied the power density at different frequencies. I would like to ask whether the light intensity will affect the output performance of S-TENG.

7. Diodes (ST4148) are the small high-speed switching diodes, not zener diodes, which are widely used for unidirectional conduction and isolation in circuits with high signal frequency. However, TENGs are suitable for low frequency environment and generate low frequency signals. Please explain why diodes (ST4148) are used.

8. The self-charging power system for tidal and solar energy harvesting has a high output performance and show potential applications in circuit management for large area energy harvesting from ocean. I suppose that it would be better that the authors add some application experiments in the manuscript.

9. The S-TENG has a 25.3 MW km-2 typical output under a typical ocean under sunshine condition. I recommend that authors conduct the stability and durability measurement of the S-TENG to prove whether the output performance of S-TENG will degrade as the working time increases.

10. Authors calculated the typical energy output of the energy ball (Ptoutput) by the input power under typical ocean conditions (Ptinput) and the output performance of energy ball in this work. It would be better that energy conversion efficiency is calculating, which could fully embody the advantages of the S-TENG.

11. There are several format and spelling errors that need to be corrected.
1) Page 7, 130th line: "…surface (φsurface)…" should be modified as "…surface (φsurface)…".
2) Variables in the article should be italicized.
3) Page 2 in the Supporting Information: …caculated (calculated) in this work…And so on.

Reviewer #3 (Remarks to the Author):

The authors skillfully combined the shadow-tribo-effect,conventional TENG, and a fiber-supercapacitor into a hybrid energy harvesting system. Such a hybrid system which demonstrated a large power output indeed shows great pontial in the efficient collection and storage of the tidal and solar energy simultaneously. Such a good idea should play a positive role in promoting the development of the intergreted energy conversion system.
In addition, the experiment was carried out in a very detailed manner and the manuscript was well organized. So, i recommend that the manuscript can be accepted without any modification.

Comments from Reviewer 1:

The manuscript demonstrates a device that can simultaneously harvest wave energy and optical energy in ocean, based on a triboelectric nanogenerator and a shadow-effect optical energy harvesting device. A self-charging power system was fabricated by integrating the energy harvester with a fiber-supercapacitor. The novelty is not quite enough considering that the shadow-effect device has been reported by the authors in their previous papers (DOI: 10.1039/d0ee00825g). Meanwhile, the coupling of the TENG and the shadow-effect device seems not quite obvious, and the performance is not improved enough compared with previously reported high-performance TENG devices.

Response: Thanks for your comments. Since invented in 2012, TENG has gained much attention for converting ambient mechanical energy into electricity efficiently and has been demonstrated as a potential solution to sustainable and reliable blue energy harvester. In order to realize the blue energy dream, there are still many works need to be done. A typical TENG alternately generates power by two materials with different charge affinities periodic contacting. Many reported blue energy harvesters are composed of a TENG and a moving friction object. The shadows of friction objects will be cast on the surface of the materials that is underneath when they are illuminated. Shadow casting are ubiquitous but not much use has been found in TENG. When the TENG integrating with solar cell to form a hybrid nanogenerator, shadow from friction object is even deleterious to output performance of the photovoltaic unit. To this end, we creatively utilized the shadow-effect to realize a hybrid S-TENG, which can overcome this issue. Although the shadow-effect has been demonstrated to harvest light energy in our previous paper (Energy Environ. Sci., 2020, 13, 2404), the integration with TENG shows great potential in blue energy harvesting, suggesting that this work is an important progress in blue energy harvesting and application of SEG. Similarly, even TENG has been reported for a long time, its application in blue energy harvesting demonstrated by Prof. Zhonglin Wang is still a great progress towards energy harvesting and application of TENG. In addition, a key challenge of TENG is its relatively low output current. In this

manuscript, we introduce a simple, low-cost S-TENG which can harvest energy from both shadows casting and period contact with friction object. There are 3 substantive differences between shadow-effect device (SEG) reported in our previous paper and this S-TENG. Firstly, the S-TENG is a hybrid device which can harvest not only light energy but also mechanical energy while the SEG is a photovoltaic device can only harvest light energy. Secondly, the working mechanism of S-TENG is different from SEG. The S-TENG hybrids tribo-effect and shadow-effect in one device. As these two effects share the same electrodes, and they are connected in parallel. Thirdly, when charging the F-SCs, the higher voltage from tribo-effect of S-TENG compensate the limitation of shadow-effect by harvesting wave energy. The rectified V_{oc} of tribo-effect from S-TENG is about 4.1 V, which is much higher than the V_{oc} of shadow-effect (0.2 V). This makes the V_{oc} of F-SCs can be charged much larger than 0.2 V. In addition, comparing with only harvesting wave energy, it can be shortened by 636 s to charge the F-SCs to the same voltage (0.3 V) by the S-TENG. This result implies that the S-TENG can harvest both light energy and mechanical energy and charge the F-SCs with a shorter time by shadow-effect enhancement. Last but not least, the cost of SEG is only 10% of that of commercial solar cell, which will be a great benefit for realization of blue energy harvesting.

1. Why a 15 nm Au layer is adopted should be clearly discussed. Is there any thickness effect? The transmittance and the resistivity should also be characterized.

Response: The reason we adopted 15 nm Au layer is that with increasing of the thickness of Au film, its transparency will greatly decrease. The decreased transparency of Au film limits light reaching n-Si and thus decreased output of Au/n-Si system under 1 sun illumination (100 mW cm^{-2}). We systematically investigated the influence of thickness of Au film on its transmittance, resistivity, and output of the Au/n-Si system. We added these results in the revision as Figure 2c and Figure S3. The relevant analysis and discussion were added in the revision as follows: "With the thickness of Au film on n-Si changing from 15, 60, to 240 nm, the relative current densities J_{15}/J_{15}, J_{60}/J_{15}, J_{240}/J_{15} and relative voltages V_{15}/V_{15}, V_{60}/V_{15}, V_{240}/V_{15} are shown in Figure 2c. J_{15}, J_{60}, J_{240} are short-circuit current densities and V_{15}, V_{60},

V_{240} are open-circuit voltages of Au/n-Si system with different thickness of Au film. All of these currents and voltages of Au/n-Si systems are measured under 1 sun illumination (100 mW cm^{-2}) and half-in-shadow condition. The current and voltage generated by Au/n-Si with 15 nm Au film are much larger than that generated by Au /n-Si system with 60 nm and 240 nm. The transmittance of the Au films with 15 nm, 60 nm, and 240 nm are shown in the inset of Figure 2c. The transmittance of Au film decreases with the increasing thickness, which limits the light reaching n-Si and thus the number of photocarriers generated. Therefore, the 15 nm Au film was adopted to fabricate the S-TENG. The electrical resistance of Au film with thickness of 15 nm, 60 nm, 240 nm are shown in Figure S3, which decreases from 39.1 Ω, 18.2 Ω to 3.4 Ω."

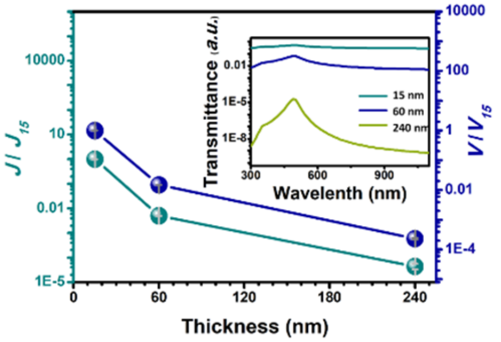

Figure 2 (c) Relative current density j/j_{15} and relative voltage V/V_{15} versus the thickness of Au film of the Au/n-Si system. Inset is transmittance of the Au film with different thicknesses.

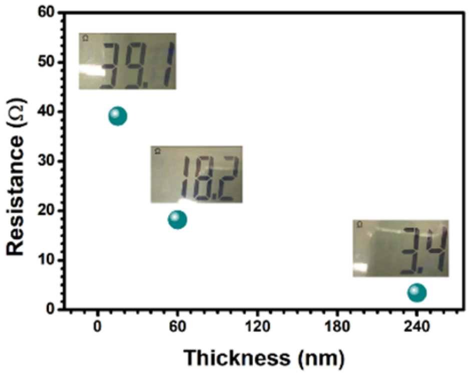

Figure S3 Electrical resistance of Au film versus thickness of Au film.

2. The mechanism shown in Figure 2c is not correct. The figure shows that two sides of a single layer of Au film is connected to a load. In such a circuit, there should be no current flowing from the load, and charges will balance the potential by moving directly

in the Au layer. Two exceptions are: the Au layer also has a high resistivity due to the thin thickness, or the external circuit is short circuited. In such cases, there will be partial current flowing from the external circuit. Please check the figure and experiment setup.

Response: Thank you for your comments. To clearly elaborate the working mechanism of the entire system, we removed part of Au film in the middle to make sure the electrodes separated by a gap. Under dark conditions, the working mechanism of S-TENG is mainly based on tribo-effect (TE). The TE mechanism is revised as Figure 3a and relevant analysis were added in the revision as follows:

"As displayed in Figure 3a, after an opaque object is brought into transient physical contact with S-TENG, the PDMS retains a layer of negative surface charges that do not dissipate over an extended period of time. When the opaque object is reaching the surface of S-TENG, the positive charges screen the negative triboelectric charges on the PDMS surface. Opposite polarity charges are induced on Au electrodes (Figure 3a (i)). As shown in Figure 3a (ii), when the opaque object leaves S-TENG, unbalanced electric potential between the two Au electrodes drives free electrons to flow from right electrode to left electrode. As the opaque object keeps moving up from the surface of the PDMS, the electrons between electrodes achieve equilibrium (Figure 3a (iii)). As the opaque object moves down again, free electrons flowing from left electrode to right electrode which induces the charges on both electrodes reversing. (Figure 3a (iv)). If the opaque object moves up and down periodically, continuous electric output could be generated. Thus, the S-TENG produces an alternative current (AC) pulse output during a periodic contact with an opaque object under dark condition."

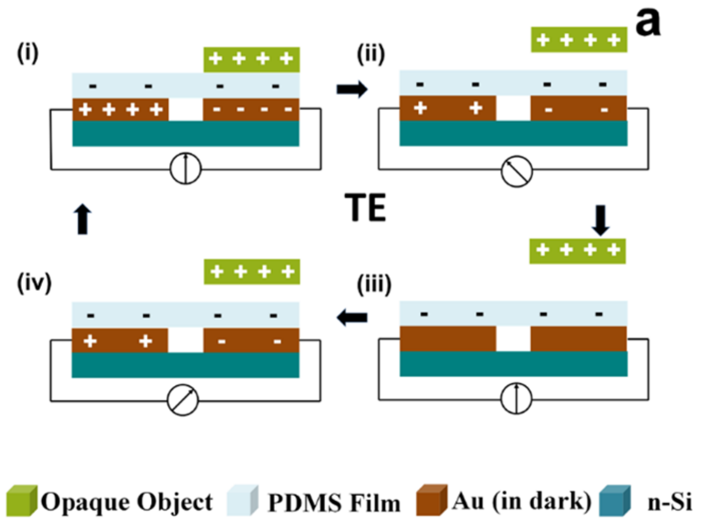

Figure 3 (a) Step-by-step illustration showing the tribo-effect (TE) working principle of the S-TENG under dark condition.

3. Similar situation as the above exists for Figure 2d. Please clearly explain why the current does not flow directly in the Au layer, but from the external load. The mechanism of the shade effect should be clearly provided. If the phenomenon is right, it seems that the shadow effect is due to the diffusion of excessive electrons from the light side to the dark side, just like a piece of metal with different temperatures.

Response: Thank you for the suggestion. To confirm the working mechanism of the system, we made a gap in the Au film by removing its middle part by 1 cm. We observed the output from the system associated with shadow effect and the J_{sc} greatly increases from 8.5 μA cm^{-2} to 39 μA cm^{-2} while no obvious change in V_{oc} was observed when the Au/n-Si was half-shadowed (Added in revision as Figure 2e). Therefore, the working mechanism of shadow-effect is based on the work function shift of Au film by excitation of electrons under light illumination. The overall J_{sc} of the device depends on two factors that are electron generation and electron transport to the dark side from where it can be collected. In original situation (Au film unremoved), parts of the excited electrons by shadow-effect flow to dark side directly on Au film, thereby leading to a decline in J_{sc} of the device. In case of the middle Au film removed, the electrons can only flow from external circuit which makes the electron transfer more efficiently. To further verify that the shadow-effect still works

after middle Au film being removed, a transparent glass with and without black paper on it was used to cast shadow on the Au/n-Si system. The transmittance of the transparent glass without and with a black paper on it are 80% and 0%, respectively. (Added as Figure S5 in the revision) As there is less light passing through glass with a black paper on it, the J_{sc} of the Au/n-Si system with half-in-shadow is larger than that without black paper on the transparent glass. (Added as Figure 2f in the revision) This is because that illumination contrast between shadow region and illuminated region of Au/n-Si system under glass with a black paper is much larger than under transparent glass without a black paper. In other words, darker shadow improves output of shadow-effect, which works after middle Au film being removed. We revised the mechanism of the S-TENG under light illumination and added the detailed explanation as follows:

"With light illumination, the working mechanism of SE is shown in Figure 3b. The opaque object is reaching the surface of S-TENG and casts a shadow on half of the surface area of the S-TENG, the work function of Au electrode under opaque object's shadow is φ_m, which is higher than that of the illuminated left electrode (φ_m^*), driving free electrons to flow from the right electrode to left electrode through external circuit (Figure 3b (i)). In stage (ii), when the opaque object moves away from S-TENG, light illuminates on all S-TENG, the illumination contrast between left and right part of S-TENG decreases. Work function of right electrode shift to $\varphi_m{}^*{}_1$, which is lower than φ_m while higher than φ_m^*. There are less free electrons flowing from the right electrode to left electrode than (i). As the opaque object keeps moving up, the work functions of electrodes all reach φ_m^* and there are no electrons transferred (Figure 3b (iii)). As the opaque object moves down again, free electrons flow from right electrode to left electrode. (Figure 3b (iv)). The S-TENG engenders a direct current (DC) output during a periodic up and down movement of an opaque object under illumination. Besides, when the opaque object moves from right side to left side, the direction of current in the external circuit is also reversed. Thus, a periodic opaque object moving from left to right on S-TENG generates AC output under illumination."

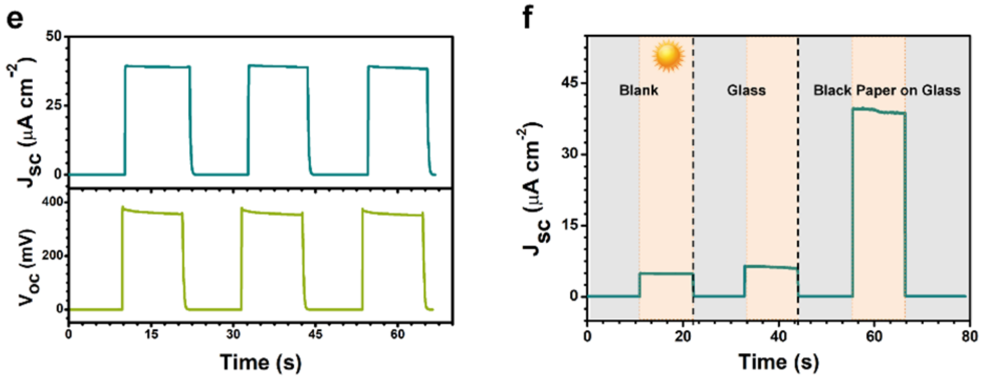

Figure 2 (e) J_{sc} and V_{oc} of the Au/n-Si system after removing 1 cm Au film in middle. (f) Performance of Au/n-Si system after removing middle Au film half-shadowed by different shadow-throwing objects (blank, glass, black paper on glass).

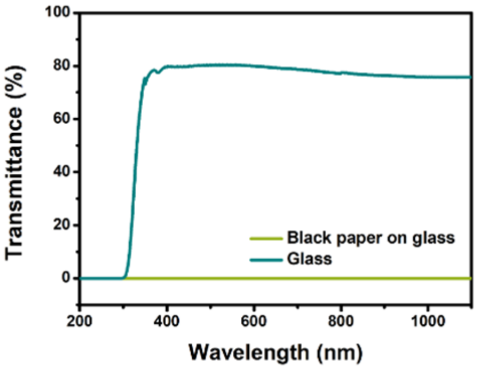

Figure S5 Transmittance of glass with or without black paper on it.

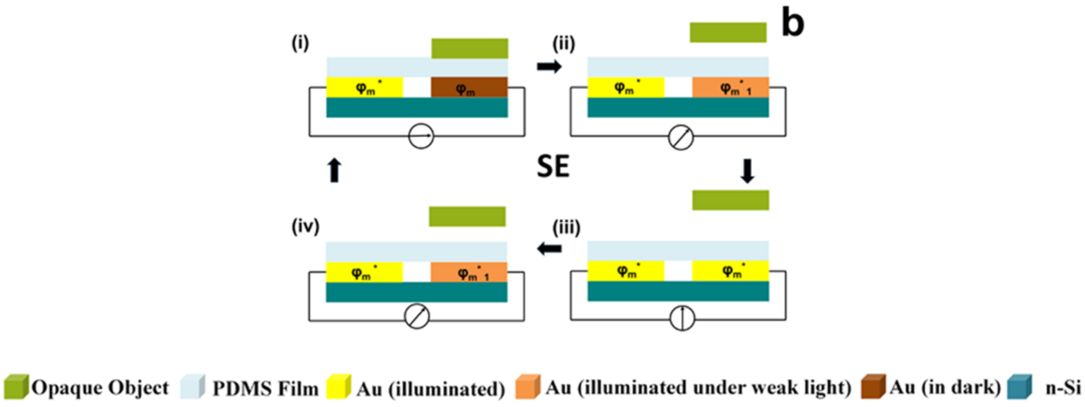

Figure 3 (b) Step-by-step illustration showing the shadow-effect (SE) working principle of the S-TENG under illumination condition.

4. The TENG and the shadow effect seem not quite coupled. So clear investigation on each effect is necessary. For the shadow effect, more characterization without the

PDMS is necessary.

Response: Thank you for your suggestion. We have supplemented more characterization on shadow effect without PDMS. The thickness effect was investigated by changing Au film on n-Si (15, 60, and 240 nm). As discussed in the response to Question 1, the thickness of Au film obviously determined the performance of the device. We find that the Au/n-Si provides best performance under half-shadowed condition. (Added as Figure 2d). We also performed additional experiments by adding a gap between Au film to clearly elaborate the working mechanism of shadow effect without PDMS, as discussed in the response to Question 3. Besides, we also studied the dependence of performance of shadow effect on light intensity of shadow by using transparent glass with or without black paper to be the shadow-casting objects. We find a shadow-casting object with less light passing through is better for improving output of shadow-effect. The relevant analysis was added in the revision (descriptions of Figure 2c, d, e, f, and Figure S5).

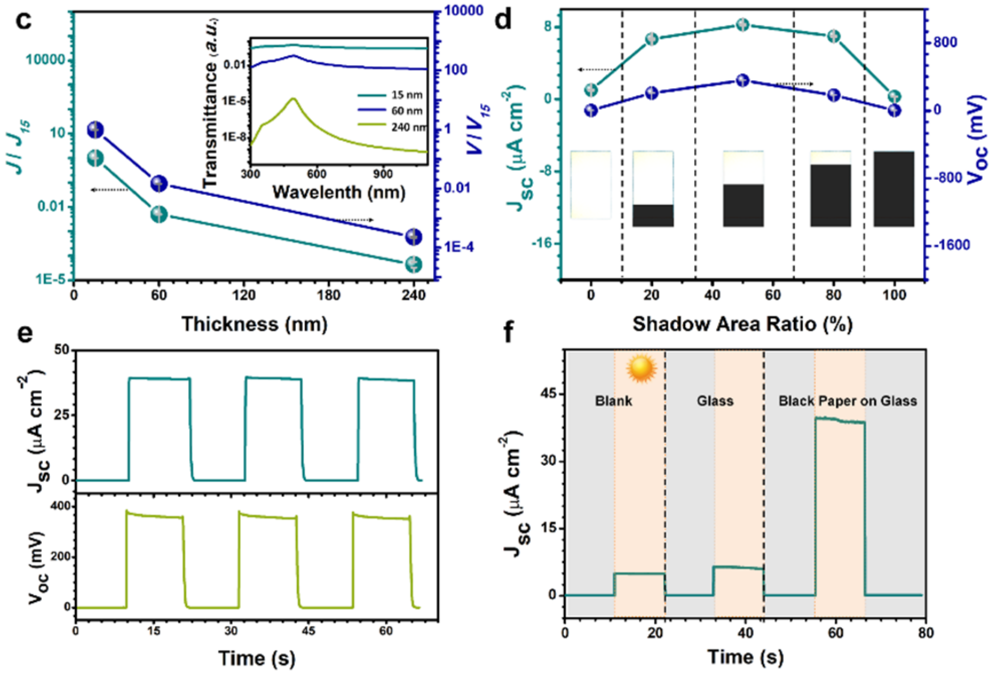

Figure 2 (c) Relative current density j/j_{15} and relative voltage V/V_{15} versus the various thickness of Au film for the Au/n-Si system. Insert is transmittance of the Au film with different thicknesses. (d) Effect of the ratio of shadow area on performance of Au/n-Si. (e) J_{sc} and V_{oc} of the Au/n-Si system after removing 1 cm Au film in middle. (f) Performance of Au/n-Si system after removing middle Au film half-shadowed by different shadow-throwing objects (blank, glass, black paper on glass).

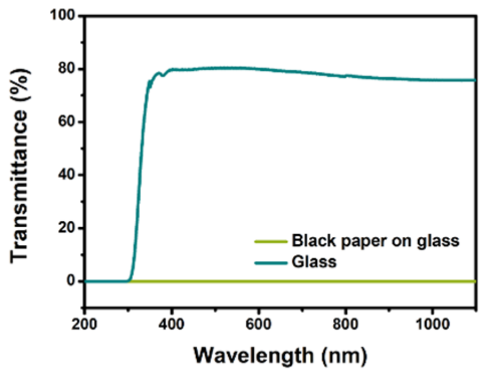

Figure S5 Transmittance of glass with or without black paper on it.

5. The experiment condition should be precisely controlled for characterization. Please don't use hand as the major method, which cannot be well controlled.

Response: In order to accurately study the output performance of S-TENG, a force gauge testing system (Mecmesin, MultiTest 2.5-i) is used to replace hand for output characterization. The force gauge testing system is mainly composed of a fixed platform and a moving stage. Distance between moving stage and fixed platform, force magnitude and moving speed of the moving stage can be well controlled. The moving stage moves towards the S-TENG with pre-set speed until force reaches 0.1 N. Then the moving stage moves apart from the S-TENG to the original position and begins next cycle. The details of experiment have been added in the revision. The relevant analysis was added in the revision as follows:

"As shown in Figure 3d and Supplementary Movie S1, the J_{sc} measurement was carried out by the moving stage of a force gauge towards and away from the S-TENG to control the experiment condition. A lamp was used as the light source. Under dark condition, a typical tribo-induced AC signal with peak J_{sc} about 0.01 μA cm^{-2} was generated. Once illuminated, the S-TENG generated a DC signal with peak J_{sc} about 34 μA cm^{-2}. A light intensity of about 90 mW cm^{-2} was used for illumination."

"The outputs of S-TENG in various contact frequencies ranging from 0.02 Hz to 0.1 Hz under dark and illumination conditions are shown in Fig. 4b, respectively. The frequencies were determined by programming the force gauge. The J_{sc} of the S-TENG increases obviously with the increase of frequency under dark condition. The current density of the S-TENG can be written as:

$$J_{sc} = d\Delta\sigma_{sc} / dt = (d\Delta\sigma_{sc} / dx) \times v \quad (3)$$

Where x is the displacement, v is the variation velocity which is a measure of frequency. Under dark condition, $\Delta\sigma_{sc}/dx$ in equation (3) can be considered as a constant, thus the J_{sc} will increase with the increasing velocity. Under illumination condition, there is no obvious change on J_{sc} as the frequency changes. As shown in Figure S7, the V_{oc} keeps constant when the S-TENG was half-shadowed by moving stage up and down with virous frequency."

The figures (Figure 3d, Figure 4b, Figure S7) are listed below.

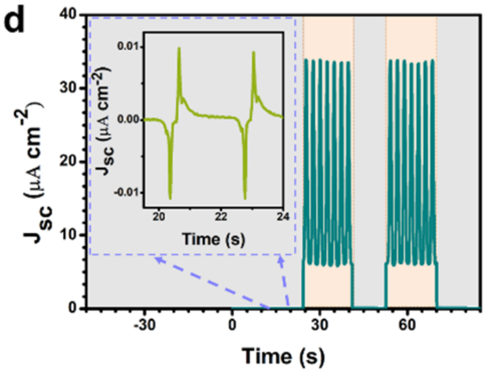

Figure 3 (d) J_{sc} of the S-TENG in dark and illumination by moving stage of force gauge up and down. Insert is the details of the output performance from 19.5 s to 24 s.

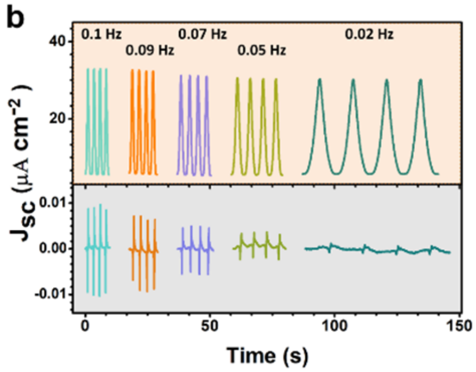

Figure 4 (b) Influence of the moving stage up and down frequency on the output under dark and illumination conditions.

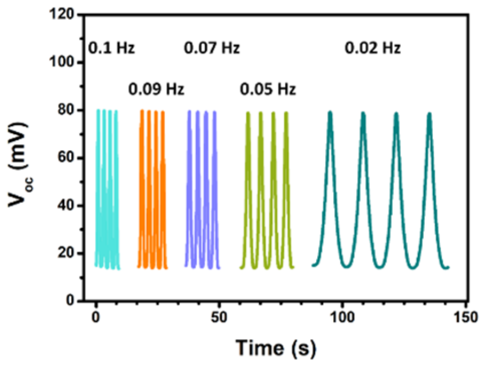

Figure S7 Influence of the moving stage up and down frequency on V_{oc} under illumination conditions.

6. The quality of data should be improved. The peaks of signals are not quite stable. Meanwhile, magnified detailed waveforms for the output should be provided.

Response: Thank you for your suggestions. We have used a force gauge to replace finger to improve the quality of data. The magnified detailed waveforms for the output of S-TENG under dark and illumination conditions have been added as Figure S6 in the revision. The discussion is added as follows:

"As shown in Figure S6, when the shadow of stage casting on S-TENG as stage moving down under illumination, the J_{sc} increased. The Figure S6a shows the magnified detailed waveforms of S-TENG under dark condition, which is a typical tribo-effect output with AC signal. When moving stage downwards above half of S-TENG under illumination, the J_{sc} increased to about 34 μA cm^{-2} (Figure S6b). As moving stage upwards, the J_{sc} decreased. The J_{sc} doesn't decrease to 0 μA cm^{-2} because there's an illumination contrast already existing which was caused by light source having an incidence angle on S-TENG."

The Figure S6 is listed below.

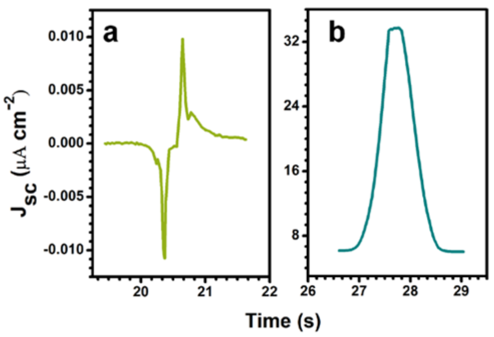

Figure S6 The magnified detailed waveforms for the outputs of half S-TENG a) under dark and b) illumination conditions.

7. The results in Figure 2a is quite unclear. How are such data measured by the SKPM? The dimensions are also missing.

Response: We have added the dimensions and analysis of KPFM in Figure 2a and Figure S2 in the revision. The relevant analysis was also added as follows:

"AFM and KPFM were adopted to characterize the surface property of Au/n-Si surface and confirm the work function shift under dark and illumination conditions. The roughness of Au/n-Si surface was obtained by a Pt/Ir tip under contact mode of AFM. The fine-grained structure of the Au film on n-Si are shown in Figure S2c and Figure S2d. Then the KPFM scan was performed under two distinct conditions- light on and light off which signifies the solar illumination and dark conditions. As shown in Figure 2a, a higher surface potential was observed under illumination. The work function of surface ($\varphi_{surface}$) before and after illumination can be calculated as:

$$\varphi_{surface} = \varphi_{tip} - CPD \times e^{-} \tag{1}$$

CPD is contact potential difference, φ_{tip} is the work function of the Pt/Ir tip which was used for the KPFM scan. The work function of Au/n-Si surface shifted from 5.26 eV to 5.39 eV under light illumination (20 mW cm^{-2}). The detailed surface potential of Au/n-Si with 15 nm Au film in dark is shown in Figure S2a while under illumination is shown in Figure S2b.".

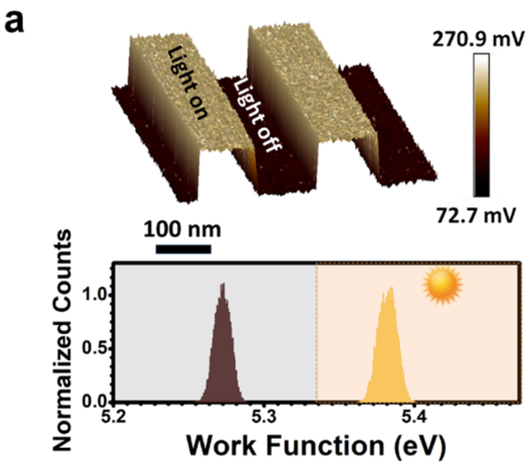

Figure 2 (a) Surface potential map with two light on/light off cycles and work function shift of the dark and illumination (20 mW cm^{-2}).

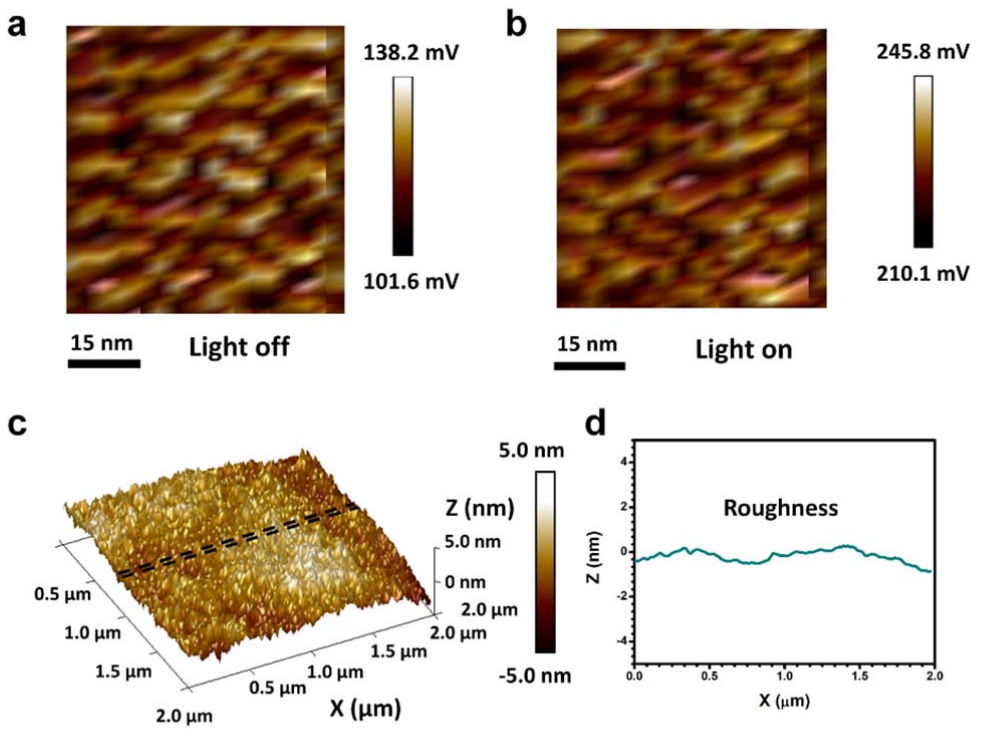

Figure S2 Surface potential maps of Au/n-Si with 15 nm Au film under a) light on and b) light off. c) AFM image of the Au film on n-Si. d) The surface roughness data obtained from cross section of black dashed line in c).

8. The coupling of the TENG and the shadow effect seems not quite strong here. They seem working relatively independently. Meanwhile, the electron flow directions seem opposite under the same operation (contact or separation) for the two mechanisms according to Figure 2.

Response: The S-TENG hybrid tribo-effect and shadow effect in one device. Under dark condition, the working mechanism of S-TENG is mainly based on tribo-effect (TE). With light illumination and opaque object not contacting on surface of S-TENG, the working mechanism of S-TENG is mainly based on shadow-effect (SE). The relevant analysis was added as follows:

"The equivalent circuit model of S-TENG which hybrids TE and SE is shown in Fig. 3c. As these two effects share the same electrodes, they are connected in parallel. In TE case, oppositely charged surfaces with a changing gap distance can be viewed as a capacitor with varying capacitance (C_T), this perspective gives a more intuitive representation of the TENG using the capacitor model. The equivalent circuit of SE is

an open-circuit voltage source connected with a resistance (R_s). The whole circuit also satisfies Thevenin's Theorem, whose output voltage (V_{AB}) is:

$$V_{AB} = -\frac{Q}{C_T} + V_{TE} = V_{SE} - IR_s \tag{2}$$

Where Q is transferred charges, V_{TE} is open-circuit voltage of S-TENG with TE, V_{SE} is open-circuit voltage of S-TENG with SE, I is current in circuit without load."

As shown in Figure 3a and b, the electron flow directions of the TE and SE in stage (ii) is opposite while in stage (iv) is the same.

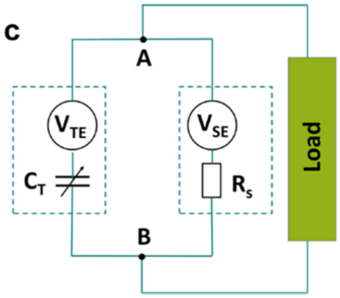

Figure 3 (c) Equivalent circuit model of S-TENG hybrid tribo-effect and shadow-effect.

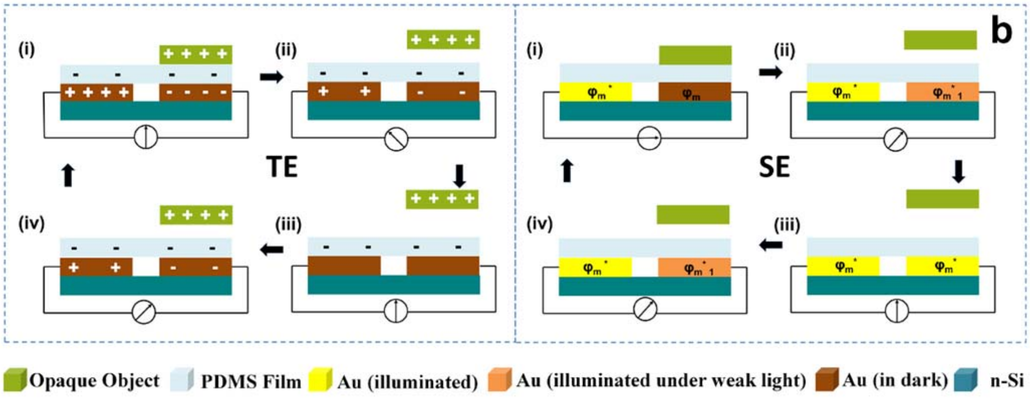

Figure 3 Step-by-step illustration showing the (a) tribo-effect (TE) and (b) shadow-effect (SE) working principle of the S-TENG under dark and illumination conditions.

9. Is the output current of the shadow effect a continuous one or a pulse one? From Figure 2b, it is continuous, but from other data, it seems a pulse one. If it is a continuous one, why it is related with frequency as in Figure 3c? Meanwhile, for a continuous current, the charging rate under illumination should be much higher than that shown in Figure 6f.

Response: The output current of the shadow effect is a continuous one when half of

the device is in stationary shadow. The mechanism and result have been added in reversion as Figure 3b and Figure 3d. However, when the shadow is moving from one half to another half of the device continuously, the output current of shadow effect changes to be a pulse one. The phenomenon is recorded and added in reversion as Supplementary Movie S2. The related results with moving shadow have been added in revision as Figure 4d. This is because direction of the current could be changed when work functions of two electrodes are reversed. The J_{sc} of the S-TENG increases obviously with the increase of frequency under dark condition while there is no obvious change on J_{sc} and V_{oc} as the frequency changing under illumination condition (Figure 4b and Figure S7). This is because the working mechanism of the S-TENG is mainly based on tribo-effect in dark condition while the mechanism is mainly based on shadow-effect under illumination.

The revised circuit diagram and charging curve of the self-charging power system is shown in Figure 6d. The S-TENG can provide a much higher output voltage after rectified when it is stimulated by waves. The switch 1 (SW1) was closed to charge 5 F-SCs in series by only shadow of Al ball. In this stage, the voltage of F-SCs increased sharply from 0 to 0.2 V in 4 s. Then the SW1 was open and switch 2 (SW2) was closed, a higher voltage from TE of S-TENG compensates the limitation of SE by harvesting wave energy simultaneously. The generated current of the S-TENG is transferred from AC to DC by a rectifier. After the F-SCs was charged, SW2 was open and SW3 was closed to power the load. Under light illumination and without rectification, the F-SC can only be charged to 0.2 V by the SE. When the energy ball was stimulated by both wave and shadow, it could be charged to a larger voltage than single SE. When the energy ball was only stimulated by wave, it took 950 s to charge the F-SCs to 0.3 V. When both wave and shadow was used to stimulate the energy ball, the charging time was significantly shortened to 314 s. This result implies that the S-TENG can harvest both light energy and mechanical energy and charge the F-SCs with a shorter time by both SE and TE. The relevant analysis was added in the revision. The figures are listed below.

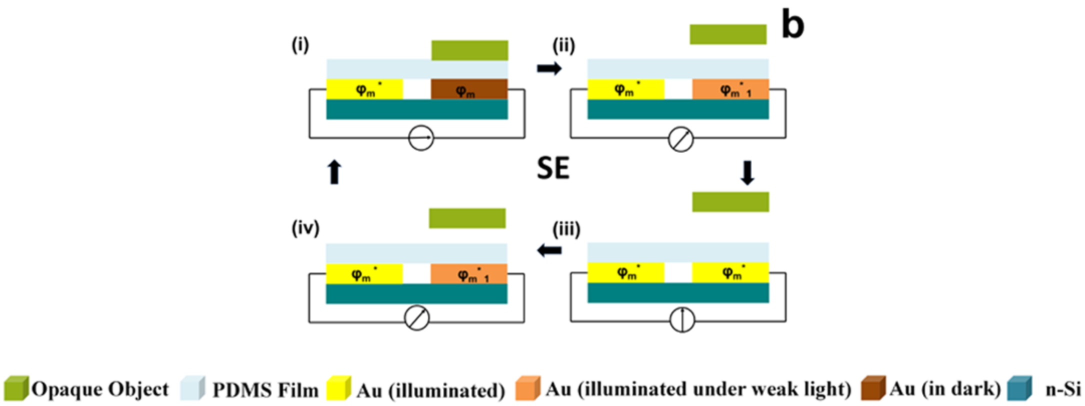

Figure 3 (b) Step-by-step illustration showing the shadow-effect (SE) working principle of the S-TENG under illumination condition.

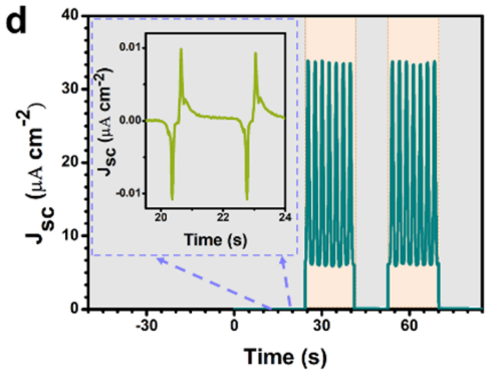

Figure 3 (d) J_{sc} of the S-TENG in dark and illumination by moving stage of force gauge up and down. Insert is the details of the output performance from 19.5 s to 24 s.

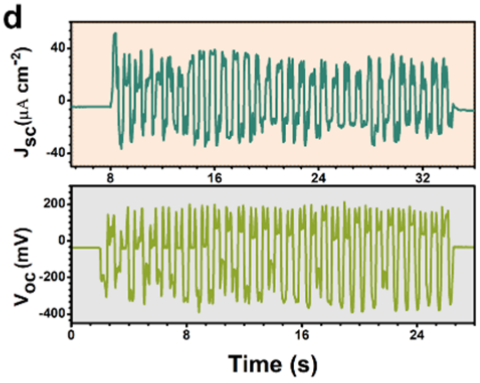

Figure 4 (d) J_{sc} and V_{oc} of S-TENG under moving shadow from one half to another half.

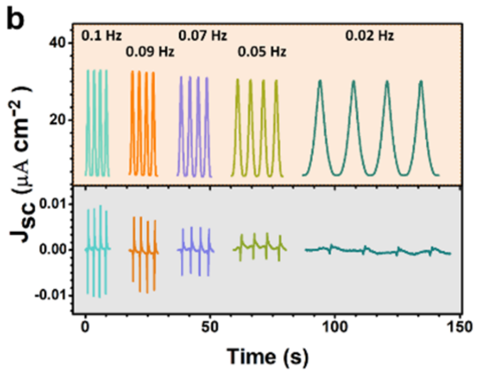

Figure 4 (b) Influence of the moving stage up and down frequency on the output under dark and illumination conditions.

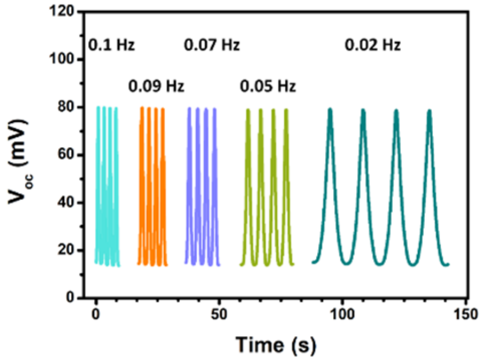

Figure S7 Influence of the moving stage up and down frequency on V_{oc} under illumination conditions.

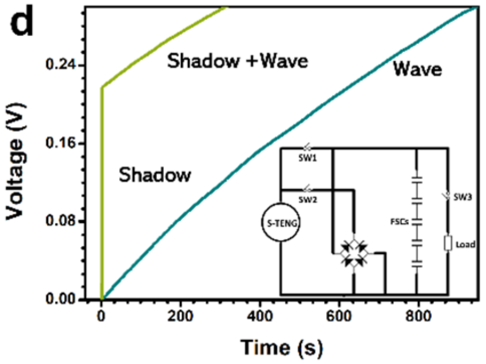

Figure 6 (d) Energy ball charging curve under single illumination (Shadow), illumination mixed wave (Shadow + Wave) and single wave (Wave). Insert is electric circuit of the S-TENG based self-charging power system and load.

10. The droplet experiments seems not quite relevant to other parts of the manuscript.

Response: Thank you for your comment. We have moved the droplet part into Supporting information as Fig. S4. The droplet experiments were conducted to prove

that the PDMS covers on Au/n-Si system could generate power based on triboelectric effect. In addition, the output of transparent water droplets on PDMS/Au/n-Si does not increase after it was illuminated, which indicates that the output increasing of the S-TENG is caused by shadow-effect.

11. The tidal energy and the wave energy are not the same thing. Please do not confuse.

Response: Thank you for your comments. We agree with you the tidal energy and the wave energy are not the same thing. In this work, the energy ball was designed to harvest wave energy from ocean. We revised the title and related descriptions in the revision.

12. The references cited are a bit arbitrary. Please try to cite some representative references.

Response: Thank you for your suggestions. We have added more representative references as Ref 4, 5, 8, 14, 20, 24, 28.

13. Please provide the photo of the fabricated device.

Response: We have added the photo of the fabricated device which is listed as below. The relevant analysis was also added in the revision.

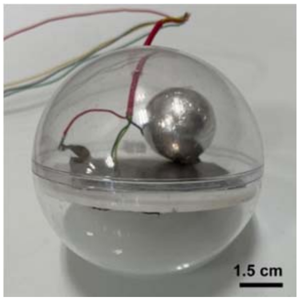

Figure S1 Photograph of the as-fabricated energy ball.

14. In the figures for illustrating the mechanism, the charge conservation should be satisfied.

Response: We have revised the figures for illustrating the mechanism and make the charge conservation in revision. The figures are listed below.

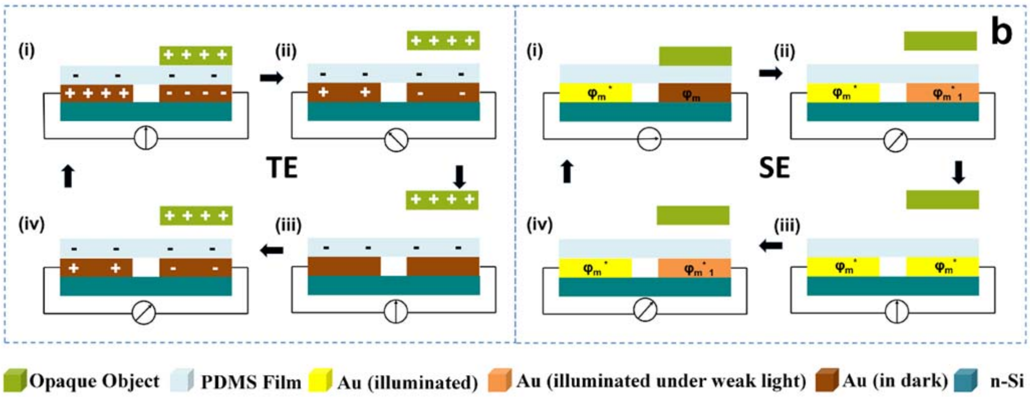

Figure 3 Step-by-step illustration showing the (a) tribo-effect (TE) and (b) shadow-effect (SE) working principle of the S-TENG under dark and illumination conditions.

15. The calculation of power density 25.3 MW km^{-2} is unacceptable. The estimation should be based on the experimental output of the device and the occupied area of the device.

Response: Yes, we agree with you that the estimation should be based on the experimental output of the device and the occupied area of the device. We have studied the output of the device systematically and the results are listed as below (Fig. 6b). The peak power of one energy ball is 10.8 mW. Four energy balls occupy an area of about 625 cm^2 in a simulated ocean environment (Inset in Figure 6f). In an ideal case, the peak power density of S-TENG based energy balls is 0.69 MW km^{-2}, which is calculated by multiplying the peak power of one energy ball by amount of energy balls per kilometer. This calculating method is the same as previous paper (*Nano Energy, 2019, 58, 499–507)*, which is cited as Ref. S4 in revised supporting information.

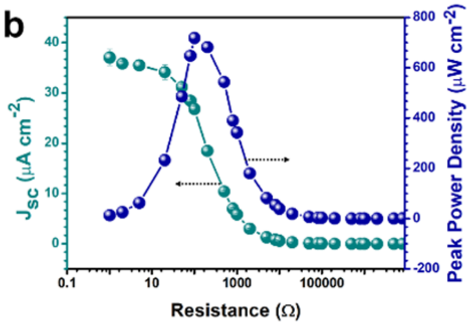

Figure 6 (b) J_{sc} and power density of S-TENG in energy ball with various loads.

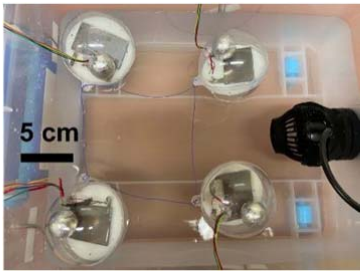

Figure 6 (f) Inset is 4 energy balls are connected in series.

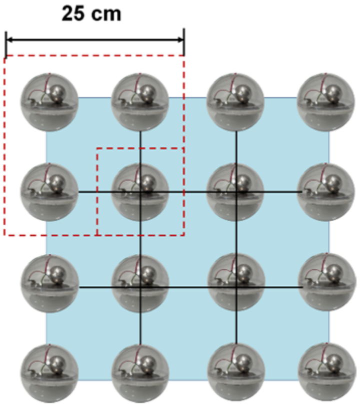

Figure S13 S-TENG based energy balls arrays for calculating the occupied area of an energy ball.

16. Some sentences are not correct, such as "When two objects with different charges are brought together".

Response: We have modified the sentences as "When two objects in different positions of triboelectric series are brought together, triboelectric charges build up on the two surfaces in physical contact".

17. "With light illumination, owing to the synergistic coupling between triboelectric-effect and shadow-effect, more electrons are generated and injected into the surface of the metal thereby enhancing the current density". The sentence seems not supported by the data and discussions.

Response: We have revised the analysis of working mechanism and delete the as mentioned sentence in revision.

18. In nature environment, the shadow of the sunlight moves as time advances, how can the device precisely positioned to enhance the output in a practical situation?

Response: Thank you for your suggestions. To simulate a nature environment, in which the shadows of the sunlight moves as time advances, a moving light source to different relative positions was used to change the incidence angle (α). The α was

changed from 0°, 45°, 90°, 135°, to 180°. When α=90°, the output reaches highest. The reason is when the α is 0°, 45°, 135°, or 180°, the shadows area is larger than that of α=90°. In addition, work-function non-uniformity on the Au/n-Si system when α is not 90° also influences the output of energy ball. The figures are listed below. The relevant analysis was also added in the revision.

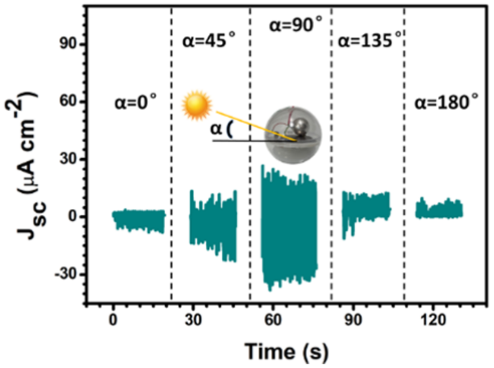

Fig. S11 Output performance of energy ball at various incidence angles.

Comments from Reviewer 2:

In this paper, a self-charging power system based on triboelectric-photovoltaics by integrating a shadow-tribo-effect nanogenerator (S-TENG) and a fiber-supercapacitor is presented, which is a method to harvest and store the tidal and solar energy. And the S-TENG is fabricated as an energy ball, generating a 25.3 MW km^{-2} typical output. However, the paper should make some modifications and further improvement, and its acceptance could be considered after major revision.

1. Please describe the substantive difference between the shadow-effect energy generator (SEG) and a shadow-effect enhanced triboelectric nanogenerator (S-TENG). It seems that a self-charging power system is only perfection and improvement of previous work and there is no new mechanism found in this work.

Response: Thank you for your comments. Since invented in 2012, TENG has gained much attention for converting ambient mechanical energy into electricity efficiently and has been demonstrated as a potential solution to sustainable and reliable blue energy harvester. In order to realize the blue energy dream, there are still many works need to be done. A typical TENG alternately generates power by two materials with different charge affinities periodic contacting. Many reported blue energy harvesters are composed of a TENG and a moving friction object. The shadows of friction objects will be cast on the surface of the materials that is underneath when they are illuminated. Shadow casting are ubiquitous but not much use has been found in TENG. When the TENG integrating with solar cell to form a hybrid nanogenerator, shadow from friction object is even deleterious to output performance of the photovoltaic. To this end, we creatively utilized the shadow effect to realize a hybrid S-TENG, which can overcome this issue. Although the shadow effect has been demonstrated to harvest light energy in our previous paper (Energy Environ. Sci., 2020, 13, 2404), the integration with TENG shows great potential in blue energy harvesting, suggesting that this work is an important progress in blue energy harvesting and application of SEG. Similarly, even TENG has been reported for a long time, its application in blue energy harvesting demonstrated by Prof. Zhonglin Wang is still a great progress towards energy harvesting and application of TENG. In

addition, a key challenge of TENG is its relatively low output current. In this manuscript, we introduce a simple, low-cost S-TENG which can harvest energy from both shadows casting and period contact with friction object. There are 3 substantive differences between shadow-effect device reported in our previous paper (SEG) and this S-TENG. Firstly, the S-TENG is a hybrid device which can harvest not only light energy but also mechanical energy while the SEG is a photovoltaic device can only harvest light energy. Secondly, the working mechanism of S-TENG is different from SEG. The S-TENG hybrids tribo-effect and shadow-effect in one device. As these two effects share the same electrodes, and they are connected in parallel. (Figure 3c) Thirdly, when charging the F-SCs, the higher voltage from tribo-effect of S-TENG compensate the limitation of shadow-effect by harvesting wave energy. The rectified V_{oc} of TE from S-TENG is about 4.1 V, which is much higher than the V_{oc} of shadow-effect (0.2 V). (Figure 6c) This makes the V_{oc} of F-SCs can be charged much larger than 0.2 V. In addition, comparing with only harvesting wave energy, it can be shortened by 636 s to charge the F-SCs to the same voltage (0.3 V) by the S-TENG. (Figure 6d) This result implies that the S-TENG can harvest both light energy and mechanical energy and charge the F-SCs with a shorter time by shadow-effect enhancement. Last but not least, the cost of SEG is only 10% of that of commercial solar cell, which will be a great benefit for realization of blue energy harvesting.

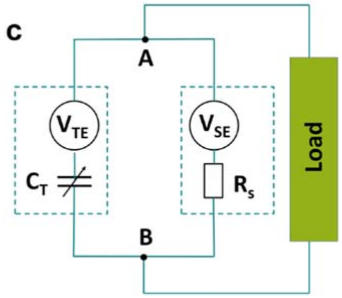

Figure 3 (c) Equivalent circuit model of S-TENG hybrid tribo-effect and shadow-effect.

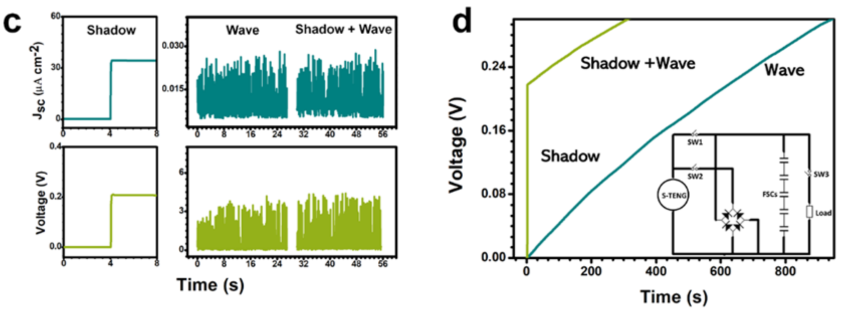

Figure 6 (c) The measured J_{sc} and V_{op} of S-TENG in energy ball before and after being rectified. (d) Energy ball charging curve under single illumination (Shadow), illumination mixed wave (Shadow + Wave) and single wave (Wave). Insert is electric circuit of the S-TENG based self-charging power system and load.

2. The named "a shadow-effect enhanced triboelectric nanogenerator (S-TENG)" is not appropriate, which is a combination of shadow-effect energy generator (SEG) and single-electrode mode triboelectric nanogenerator. In addition, the SEG based on the ubiquitous optical effect that is a photovoltaic device is the main output source of S-TENG. In terms of the comparison between different technologies for energy harvesting from ocean which all based on the TENG, I have some confusion about its rationality and please give a reasonable explanation.

Response: Thank you for your comments. Our work developed a reliable shadow-tribo-effect nanogenerator (S-TENG), which is not a simple paste of single electrode TENG on bulky or expensive solar cell like previous reports. In previous reports, most of researchers simply paste a TENG on commercial solar panels (*Nano Energy*, 66, 2019, 104188; *Nano Energy*, 2019, 57, 424-431). They used transparent friction objects (such as raindrop, wind) that harness friction to forbid shadows of friction objects influent output of solar panels. In this work, the S-TENG hybrids shadow-effect and tribo-effect in parallel by sharing the electrodes, which can scavenge both light energy that arises on its surface and mechanical energy from friction object's motion. A peak power density of 718 μW cm^{-2} is achieved by wave stimulation and light illuminating on the S-TENG, much higher than power density of the only tribo-effect (0.14 μW cm^{-2}).

Comparing with other blue energy TENG technologies, the S-TENG doesn't need

complex design intricate engineering or expensive raw materials. The fabrication of the S-TENG can be done at extremely low cost and does not involve any harmful chemicals nor intricate synthesis protocols. Comparing with photovoltaic device with complex structures, the structure of S-TENG is as simple as TENG. In addition, S-TENG obtains the energy brought by the shadow of the friction object under the light, not from solar energy directly. Therefore, we compare it with TENGs to show that S-TENG has the same simple structure as TENG but greatly improve the output.

3. "A small steel ball which is used as the opaque friction object on the S-TENG." is shown in the paper. Please explain why a steel ball is applied instead of using other materials, such as copper, which is a better tribo-positive material.

Response: Thank you for your suggestion. Steel is a better tribo-positive material than copper (ACS Nano, 2013, 7, 9533–9557). In addition, we find aluminum (Al), which is far away from PDMS in triboelectric series is a better tribo-positive material than steel. We have studied the triboelectric output of S-TENG with a steel ball and an Al ball, respectively. We observed an obvious output change of S-TENG with these two different materials. Therefore, the Al ball is chosen to be the opaque friction object on the S-TENG.

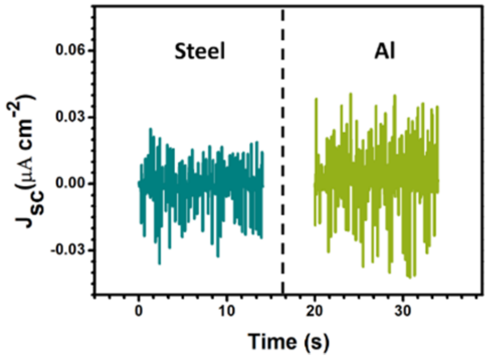

Figure S9 Triboelectric output of S-TENG with a steel ball or Al ball on it.

4. Authors depict "The working mechanism of the output 2 mode of S-TENG can be illustrated as a water-drop TENG" in this paper. The water-drop TENG is seen in characterizations of the charge generation and transfer that occur at solid-liquid or liquid–liquid interfaces. However, the S-TENG uses the characteristics of the charge generation and transfer that occur at solid-solid interface. I don't think the author's

analogy is accurate, please make corresponding amendments.

Response: Yes, the charge generation and transfer of a water-drop TENG occurs at solid-liquid or liquid-liquid interfaces. For proving PDMS covers on Au/n-Si could generate power by a tribo-effect, we used PDMS/Au/n-Si as a single electrode TENG and transparent droplet to be the friction object to generate power. As your suggestion, we have modified the "water-drop TENG" as the "single-electrode TENG" in revision.

5. Figure 3c show "Influence of the fingers up and down frequency on the J_{sc} under dark and illumination conditions". Please explain how authors ensure that the force exerted by fingers on the S-TENG is stable, which has an impact on the output performance of TENG. I recommend that authors conduct a series of experiments utilizing rotors.

Response: Thank you for your suggestion. We have conducted a series of experiments by utilizing a force gauge testing system (Mecmesin, MultiTest 2.5-i) whose movement can be well-controlled. The force gauge testing system is mainly composed of a fixed platform and a moving stage. Distance between moving stage and fixed platform, force magnitude and moving speed of the moving stage can be well controlled. The moving stage moves towards the S-TENG with pre-set speed until force reaches 0.1 N. Then the moving stage moves apart from the S-TENG to the original position and begins next cycle. The details of experiment have been added in the revision. The relevant analysis was added in the revision as follows:

"As shown in Figure 3d and Supplementary Movie S1, the J_{sc} measurement was carried out by moving stage of a force gauge towards and away from the S-TENG to control the experiment condition. A lamp was used as the light source. Under dark condition, a typical tribo-induced AC signal with peak J_{sc} about 0.01 μA cm^{-2} was generated. Once illuminated, the S-TENG generated a DC signal with peak J_{sc} about 34 μA cm^{-2}. A light intensity of about 90 mW cm^{-2} was used for illumination."

"The outputs of S-TENG in various contact frequencies ranging from 0.02 Hz to 0.1 Hz under dark and illumination conditions are shown in Fig. 4b, respectively. The frequencies were determined by programming the force gauge. The J_{sc} of the S-TENG

increases obviously with the increase of frequency under dark condition. The current density of the S-TENG can be written as:

$$J_{sc} = d\Delta\sigma_{sc} / dt = (d\Delta\sigma_{sc} / dx) \times v \quad (3)$$

Where x is the displacement, v is the variation velocity which is a measure of frequency. Under dark condition, $\Delta\sigma_{sc}/dx$ in equation (3) can be considered as a constant, thus the J_{sc} will increase with the increasing velocity. Under illumination condition, there's no obvious change on J_{sc} as the frequency changing. As shown in Figure S7, the V_{oc} keeps constant when the S-TENG was half-shadowed by moving stage up and down with various frequency."

The figures (Figure 3d, Figure 4b, Figure S7) are listed below.

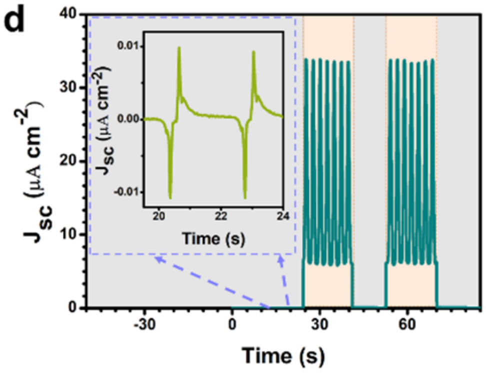

Figure 3 (d) J_{sc} of the S-TENG in dark and illumination by moving stage of force gauge up and down. Insert is the details of the output performance from 19.5 s to 24 s.

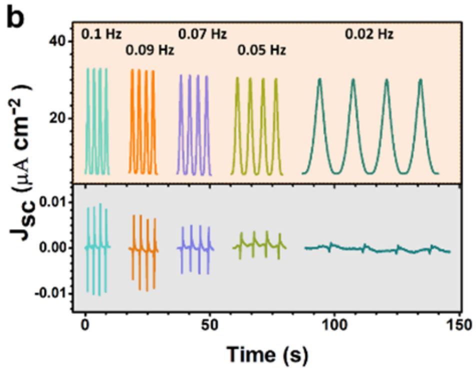

Figure 4 (b) Influence of the moving stage up and down frequency on the output under dark and illumination conditions.

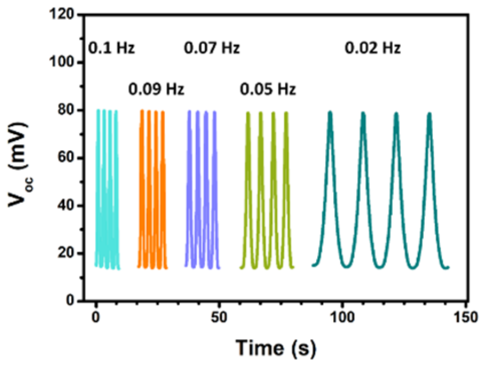

Figure S7 Influence of the moving stage up and down frequency on V_{oc} under illumination conditions.

6. The author studied the power density at different frequencies. I would like to ask whether the light intensity will affect the output performance of S-TENG.

Response: Thank you for your comments. We studied the power density at different frequencies with moving stage of force gauge under dark and illumination conditions in revision. The J_{sc} of the S-TENG increases obviously with the frequency increases under dark condition while no obvious change on J_{sc} and V_{oc} as the frequency changing under illumination condition. This proves in illumination case, the main mechanism of S-TENG is shadow-effect. In addition, we have investigated the output performance of the S-TENG under diverse light intensity. As the light intensity is increased, the current density of S-TENG will be improved. This is because under illumination condition, the n-Si is the main source of photocarrier generation of shadow-effect. With a higher light intensity, more photocarriers in n-Si were generated. The resulting performances are listed as below. The relevant analysis was added in the revision.

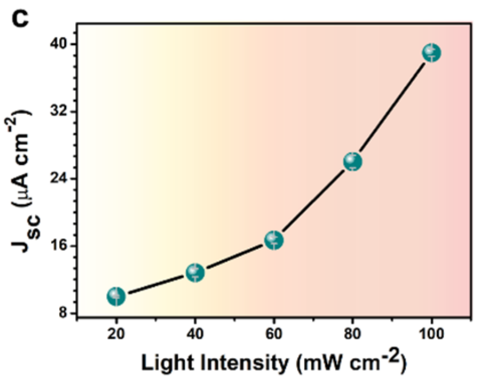

Figure 4 (c) Dependence of light intensity on output of S-TENG.

7. Diodes (ST4148) are the small high-speed switching diodes, not Zener diodes, which are widely used for unidirectional conduction and isolation in circuits with high signal frequency. However, TENGs are suitable for low frequency environment and generate low frequency signals. Please explain why diodes (ST4148) are used.

Response: Diodes (ST4148) are widely used to be high-speed switching diodes, which give break down voltage as 75 V. As the output voltage of S-TENG is smaller than 75 V, we used two ST4148 as Zener diodes to divide and rectify the output voltage of S-TENG. However, the half-wave rectification with two diodes blocks one half of the input waveform, which waste another half of input waveform. In revision, we used a full-wave rectifier (DF10S) to replace the two diodes. The updated electric circuit of the S-TENG based self-charging power system and load was added in revision as Figure 6d insert. The relevant descriptions were also revised in manuscript.

8. The self-charging power system for tidal and solar energy harvesting has a high output performance and show potential applications in circuit management for large area energy harvesting from ocean. I suppose that it would be better that the authors add some application experiments in the manuscript.

Response: We have added application experiments about using self-charging power system based on energy balls to manage circuit to split seawater. Electrolysis of seawater is utilized as an environment friendly approach for production of hydrogen (H_2). An external power supply for driving the oxidation or reduction reactions of H_2O molecules is mandatory for electrolysis. By paralleling groups of energy balls in series, the generated and stored electricity is used for sea water splitting to produce H_2. (Figure 6e) During the day or night, the energy balls could supply the energy required for water splitting continuously. A hydrogen fuel cell system collects and store generated H_2 gas. (Fig. S12). To demonstrate, a group of 4 energy balls in series was connected to split the seawater (insert in Figure 6f). A carbon fabric and a platinum sheet with the same size of 4 cm^2 were used as electrodes to perform the electrolysis reaction. The F-SCs in series are charged to 1.3 V using both shadow and waves, and

then discharged to split the seawater (Figure 6f). This shows the potential application of energy balls in circuit management for large area energy harvesting from ocean. The resulting performances are listed as below. The relevant analysis was added in the revision.

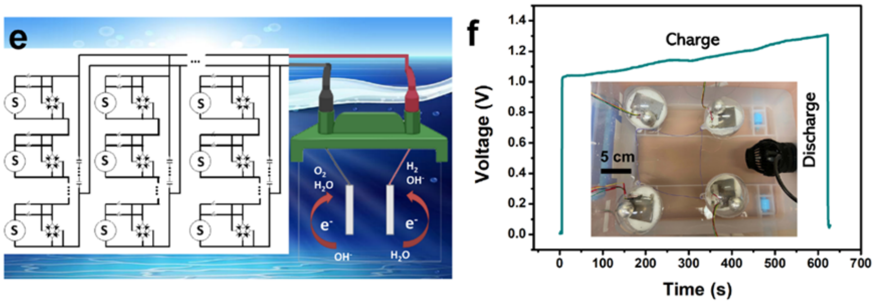

Figure 6 (e) Circuit management of S-TENG based self-charging power system applies in electrocatalysis of seawater. (f) 4 energy balls are connected in series to charge 5 F-SCs in series and then discharge the F-SCs by seawater splitting. Insert is 4 energy balls are connected in series.

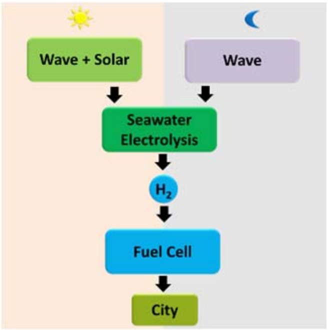

Figure S12 Process scheme of fuel cell production based on seawater electrolysis.

9. The S-TENG has a 25.3 MW km^{-2} typical output under a typical ocean under sunshine condition. I recommend that authors conduct the stability and durability measurement of the S-TENG to prove whether the output performance of S-TENG will degrade as the working time increases.

Response: Thank you for your suggestions. we examined the stability of the S-TENG as it is in the energy ball by continuously over 1000 s (Figure S10). The output of S-TENG under continiously waving and illuminating stimulation slightly changed because of ups and downs of waves. The S-TENG exhibited good stability and durability, which makes it reliable for potential application.

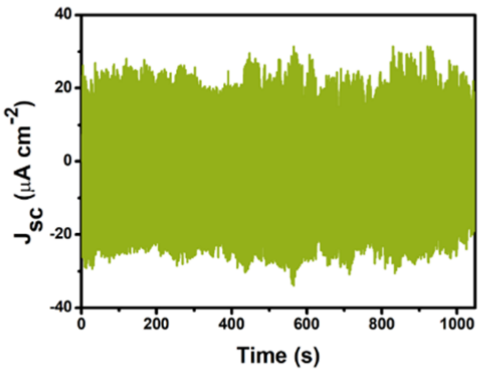

Figure S10 The stability test of the S-TENG, the measured results of J_{sc} of the S-TENG continuously operating for more than 1000 s.

10. Authors calculated the typical energy output of the energy ball (Ptoutput) by the input power under typical ocean conditions (Ptinput) and the output performance of energy ball in this work. It would be better that energy conversion efficiency is calculating, which could fully embody the advantages of the S-TENG.

Response: Thanks for your suggestions. We have added the calculation of energy conversion efficiency in revision. The energy conversion efficiency (η) of S-TENG in an energy ball can be calculated by:

$$\eta = \frac{P_{peak}}{P_{light} + P_{wave}} \times 100\% \qquad \text{(S2)}$$

Where P_{peak} is maximu peak power of S-TENG in one energy ball, P_{light} is calculated to be 1500 mW. P_{wave} is 21.4 mW, which calculated by dividing the power of wave machine (12 W) by amount of water (15 L) and multiply volume of energy ball. As shown in Fig. 6b, the P_{peak} of an energy ball is 10.8 mW. The η of an energy ball is calculated to be 0.7%.

11. There are several format and spelling errors that need to be corrected.

1) Page 7, 130th line: "…surface (φsurface)…" should be modified as "…surface (φsurface)…".

2) Variables in the article should be italicized.

3) Page 2 in the Supporting Information: …caculated (calculated) in this work…And so on.

Response: 1) Thank you for your suggestion. We have modified the format error of

"…surface ($\varphi_{surface}$)…".

2) As suggested, we italicized all of the variables in the article.

3) The spelling errors have been checked and corrected.

Comments from Reviewer 3:

The authors skillfully combined the shadow-tribo-effect,conventional TENG, and a fiber-supercapacitor into a hybrid energy harvesting system. Such a hybrid system which demonstrated a large power output indeed shows great pontial in the efficient collection and storage of the tidal and solar energy simultaneously. Such a good idea should play a positive role in promoting the development of the intergreted energy conversion system.

In addition, the experiment was carried out in a very detailed manner and the manuscript was well organized. So, i recommend that the manuscript can be accepted without any modification.

Reviewer #1 (Remarks to the Author):

The authors have made considerable efforts to improve the manuscript and many problems in the original manuscript have been solved. However, there are still problems and unclear points in the manuscript considering the high standard of Nature Communications. After careful evaluation of the responses and revised manuscript, here are detailed comments for the revised manuscript:

(1) The demonstrated structure of the device has been changed in the two editions. Whether the SEG can work both with or without a gap, why? Which figures are tested with the gap and which are not? The author should clearly state the electrode structure of the device used in the experiment for the data to avoid confusion. Does the ball device have the gap? It can be observed from the photo that there is no gap in the ball device.

(2) "Under illumination condition, there is no obvious change on Jsc as the frequency changes." The above description is quite different from the original manuscript, which claims that the output of the SEG is related to the frequency (Fig. 3c in the original manuscript). What caused the differences in the data?

(3) The working mechanism of the SEG should be clearly stated. It seems that the motion is not needed for the optical energy harvesting, and the mechanism shown in Figure 3b may make a misunderstanding that the output is triggered by motion. Many data may also make such misunderstanding with pulsed output.

(4) For the working mechanism of the SEG, only work function difference is not enough to explain the continuous current. One example is when you connect two metals that have different work functions, you will not get continuous current. There should be a loop for the conduction current. The n-Si should also play a role in forming a loop, which corresponds to part of the internal resistance in the circuit model.

(5) The structure of the ball device seems not a very efficient design. The SEG do not need a moving shadow to output, and it can even provide a continuous current instead a pulsed one without moving part. The moving of the object seems actually lowering the total output. A more efficient design is placing the SEG at an upper layer above the ball and cover half of the SEG directly. There are also problems in internal impedance mismatching for the SEG and TENG when connect them directly together in parallel (sharing the same electrodes). The SEG have a low internal impedance, while the TENG of freestanding mode has a very high impedance. Meanwhile, in some situations, the currents of the two devices seem opposite. In this regards, the two devices are not well coupled.

(6) The TENG in the ball device is based on a free-standing mode. However, the mechanism shown in Figure 3 is like a single electrode mode. The organization of the manuscript should be more consistent. Meanwhile, the adopted device structure for each data should be clear in the manuscript.

(7) The Schottky contact should be measured and verified.

(8) The power output, charge output and open-circuit voltage of the TENG should be characterized.

(9) The capacitance of the capacitor in Figure 6 should be provided.

(10) Please provide more information on the resistance measurement in Figure S3, such as the width of the film and the space between the two wiring points. Is the measurement based on pure Au film or using the structure of Au/n-Si.

(11) Some typos exist: "wavelenth" in Figure 2c, "j/j15", "by amount of energy balls per kilometer" should use area unit, "which induces the charges on both electrodes reversing" the charges are not reversing from the figure, what is "Vop".

(12) "Under dark condition, Δσsc/dx in equation (3) can be considered as a constant, thus the Jsc will increase with the increasing velocity." The detailed discussion is not quite reasonable and necessary.

(13) "In TE case, oppositely charged surfaces with a changing gap distance can be viewed as a capacitor with varying capacitance (CT)" The detailed discussion is not quite reasonable and necessary. Here, actually the capacitor in the model does not correspond to the surfaces with the changing gap.

(14) The rectification circuit in Figure 6d is not right. When SW2 is closed, it won't change AC to DC due to the wrong connection of the rectifier.

(15) What is the upper object in testing the data in Figure 2-4?

(16) The data shown in Figure 6c are hard to understand. Why the current output with shadow is so low?

Reviewer #2 (Remarks to the Author):

The authors have precisely addressed the comments. The manuscript should be accepted for publication.

Comments from Reviewer 1:

The authors have made considerable efforts to improve the manuscript and many problems in the original manuscript have been solved. However, there are still problems and unclear points in the manuscript considering the high standard of Nature Communications. After careful evaluation of the responses and revised manuscript, here are detailed comments for the revised manuscript:

1. The demonstrated structure of the device has been changed in the two editions. Whether the SEG can work both with or without a gap, why? Which figures are tested with the gap and which are not? The author should clearly state the electrode structure of the device used in the experiment for the data to avoid confusion. Does the ball device have the gap? It can be observed from the photo that there is no gap in the ball device.

Response: Thank you for your comments. We tested the outputs of SEG before (Figure 2b) and after (Figure 2e) 1 cm Au in middle of Si surface peeled off in the half-shadowed condition. The SEG can work both with or without a gap. This is because the gap doesn't influence the potential generating. The potential between illuminated part and in shadow part of Au is attributed to a work function contrast when illuminating one-half of the SEG with or without a gap. This potential will drive free electrons flow from the illuminated part to the shadowed part when the SEG is connected with an external circuit. The working mechanism remains the same for SEG with or without a gap. However, the introduction of a gap can prevent current leakage that may present in SEG without a gap, thereby increases the output of SEG.

We have clearly stated the electrode structure of the devices used in the experiments and added the S-TENG fabricated with a gap Au film in the experimental section to avoid confusion.

Yes, the ball devices have gap. The photos of ball devices with gap have been updated in revision. They are all listed as below.

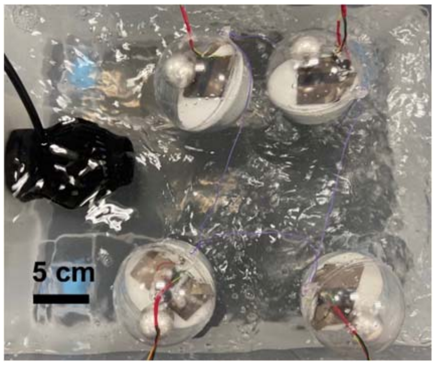

Figure 6f Inset is the four energy balls working together.

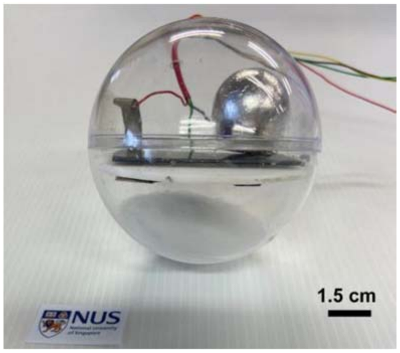

Supplementary Figure 1 | Photograph of the as-fabricated energy ball.

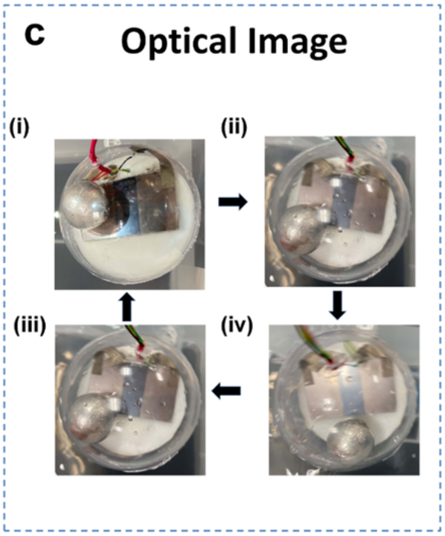

Supplementary Figure 11 | c Optical images of energy ball under four related stages shown in a and b.

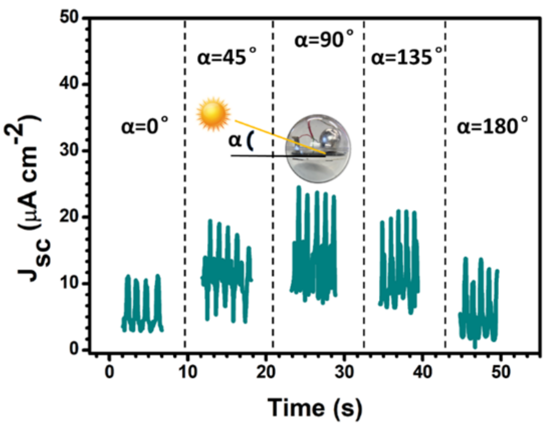

Supplementary Figure 14 | Output performance of energy ball at various incidence angles.

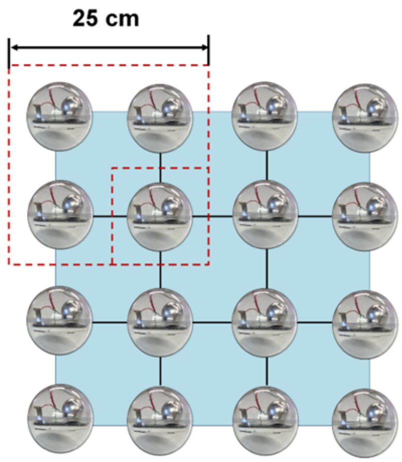

Supplementary Figure 16 | S-TENG based energy balls arrays for calculating the occupied area of an energy ball.

2.“Under illumination condition, there is no obvious change on J_{sc} as the frequency changes.” The above description is quite different from the original manuscript, which claims that the output of the SEG is related to the frequency (Fig. 3c in the original manuscript). What caused the differences in the data?

Response: In original edition, fingers are used as the moving object to characterize the output dependence. The force, contact area, and contact-separate distance exerted by fingers on S-TENG cannot be well controlled, which affects the output performance of S-TENG. In the revision, we used a force gauge testing system to replace fingers for output characterization. The force, contact area, and contact-separate distance can be fixed while the only variant is frequency. Therefore,

there are some data difference.

3. The working mechanism of the SEG should be clearly stated. It seems that the motion is not needed for the optical energy harvesting, and the mechanism shown in Figure 3b may make a misunderstanding that the output is triggered by motion. Many data may also make such misunderstanding with pulsed output.

Response: Thank you for your suggestion. We agree that motion is not the necessary condition for optical energy harvesting by SEG, so we added some explanation on this in revision. On the other hand, some opaque objects (a force gauge or ball) are used for characterization or demonstration which will cast shadows on the S-TENG under illumination. The motion of these opaque objects results in the change of shadows on the device, generating some pulse output. Therefore, to be consistent with the device design and experimental condition, we present a moving object to illustrate the working mechanism of SEG. To avoid misunderstanding, we added more discussion on the working mechanism of SEG with and without a moving object. Besides, we also deleted Fig. 4d and Supplementary Movie 2, undated Figure 6a, Supplementary Figure 13 and Supplementary Figure 14 in revision. The updated figures are listed as below.

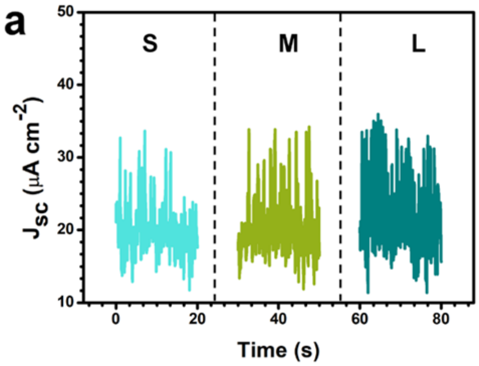

Figure 6a Intensity of wave influence on output of S-TENG in energy ball.

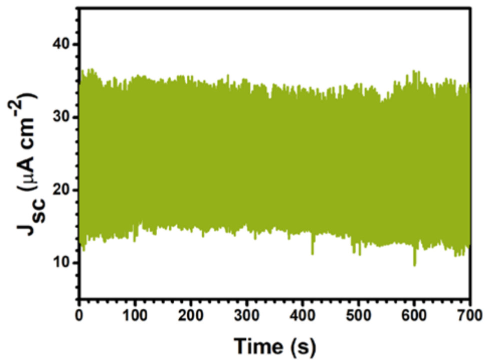

Supplementary Figure 13 | The stability test of the S-TENG, the measured results of J_{sc} of the S-TENG continuously operating for more than 700 s.

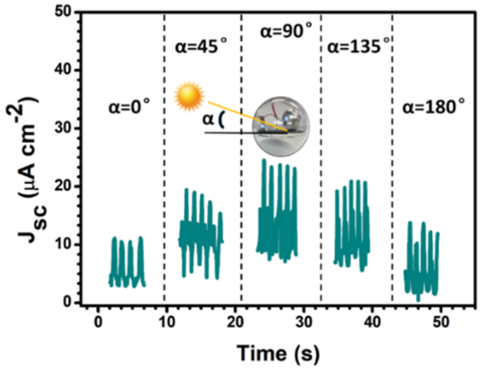

Supplementary Figure 14 | Output performance of energy ball at various incidence angles.

4. For the working mechanism of the SEG, only work function difference is not enough to explain the continuous current. One example is when you connect two metals that have different work functions, you will not get continuous current. There should be a loop for the conduction current. The n-Si should also play a role in forming a loop, which corresponds to part of the internal resistance in the circuit model.

Response: Thank you for your comments. We agree that the n-Si play a role in forming a loop. We added this statement in the revision. For the SEG, the electrons in n-type silicon below the Au film is continuously excited into the conduction band because of the continuous illumination. These electrons will then jump into the gold film and the work function contrast will drive the current to flow from the bright side to the dark side, generating a continuous current. We also added more discussion on this in revision.

5. The structure of the ball device seems not a very efficient design. The SEG do not need a moving shadow to output, and it can even provide a continuous current instead a pulsed one without moving part. The moving of the object seems actually lowering the total output. A more efficient design is placing the SEG at an upper layer above the ball and cover half of the SEG directly. There are also problems in internal impedance mismatching for the SEG and TENG when connect them directly together in parallel (sharing the same electrodes). The SEG have a low internal impedance, while the TENG of freestanding mode has a very high impedance. Meanwhile, in some situations, the currents of the two devices seem opposite. In this regards, the two devices are not well coupled.

Response: Thank you for these comments. In our design, the friction object of the tribo-effect also acts as an opaque object of the shadow-effect. This design can help solve the problem of performance deterioration of photovoltaic unit caused by shadow in hybrid energy harvester towards blue energy harvesting. As the suggestion of placing the SEG at an upper layer above the ball, it is an effective strategy for improving performance of SEG, which will provide beneficial guidance on our future optimization. As the different internal impedance between SEG and TENG, this is not necessarily an issue in our case. We integrate the S-TENG with fiber-supercapacitors, which can well store the energy from both SEG and TENG. The open-circuit voltage generated by S-TENG in an energy ball depends on shadow-effect in normal case (0.2 V as shown in Figure 6c). The fiber-supercapacitors is only charged to 0.2 V by this case. Then, the S-TENG continues to charge the F-SCs to 0.3 V by tribo-effect when S-TENG is connected to the fiber-supercapacitor with the rectifier. This is because there is a voltage drop (0.7 V for Si diode) in the rectifier, only allowing higher voltage to pass. The output of shadow-effect will be blocked by the rectifier due to its lower voltage than this barrier. As shown in Figure 6d in the revision, after the fiber-supercapacitor being charged to 0.2 V by shadow-effect, switching on the Switch 1, 2 and switching off the Switch 3 can further charge the fiber-supercapacitor by tribo-effect of S-TENG as its voltage is much higher than the barrier. In this case, the opposite currents of the shadow-effect and tribo-effect will not influence the

self-charging power system that can be charged to a higher voltage and obtain wave energy.

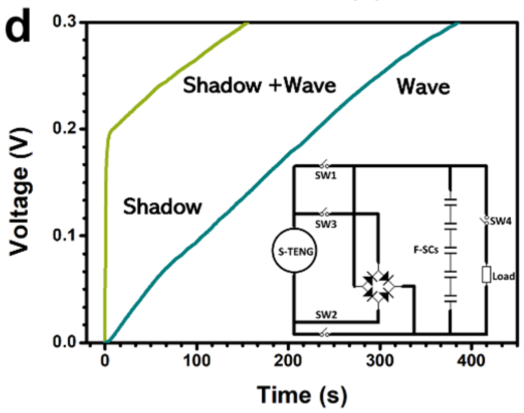

Figure 6d Energy ball charging curve under single illumination (Shadow), illumination mixed wave (Shadow + Wave) and single wave (Wave). Inset is electric circuit of the S-TENG based self-charging power system and load.

6. The TENG in the ball device is based on a free-standing mode. However, the mechanism shown in Figure 3 is like a single electrode mode. The organization of the manuscript should be more consistent. Meanwhile, the adopted device structure for each data should be clear in the manuscript.

Response: The TENG in the ball device is based on a single-electrode mode. For a better understanding, we have added the working principle of the S-TENG in energy ball and take photos of the four related stages as Supplementary Figure 11 in revision. The relevant analysis and discussion were added in the revision as follows:

"The working mechanism of S-TENG in the energy ball is mainly based on TE under a dark condition (Supplementary Figure 11). The detailed discussion is presented in Supplementary Note 11. The image of the fabricated energy ball floating on water is also shown in Supplementary Figure 11."

"**Supplementary Note 11. Working mechanism of S-TENG in the energy ball**

The equivalent circuit of the S-TENG in energy ball with an ammeter is illustrated in Supplementary Figure 11a. As being depicted in Fig. 3a, the working mechanism of S-TENG in the energy ball with an Al ball on it is mainly based on TE under a dark condition. The only difference between using moving stage and Al ball as the opaque

object is that the moving direction of the opaque object changes from vertical to horizontal direction (Supplementary Figure 11b). The optical image of the energy ball under four related stages in Supplementary Figure 11a and b is also shown in Supplementary Figure 11c."

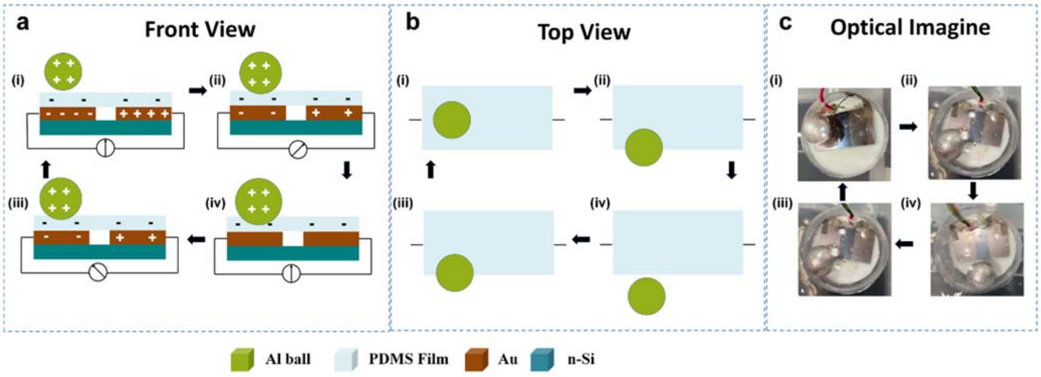

Supplementary Figure 11 | a Front view and **b** Top view of the working principle of the S-TENG in the energy ball. **c** Optical images of the energy ball under four related stages shown in a and b.

7. The Schottky contact should be measured and verified.

Response: To measure and verify the Schottky contact, 15 nm Au was sputtered on half of the n-Si (Supplementary Figure 2a). The I-V characteristics of the plain n-Si and half-Au on n-Si system were both measured under light illumination and dark condition. As shown in Supplementary Figure 2b, under forward bias and light condition, the Schottky contact can be clearly observed.

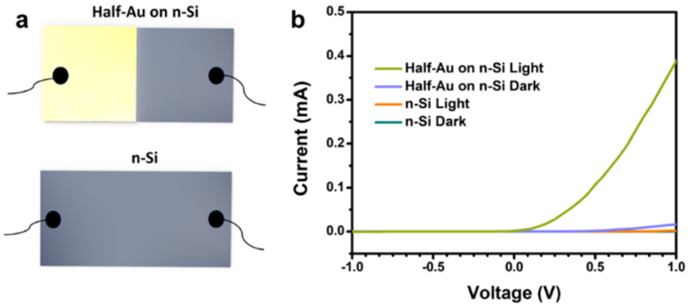

Supplementary Figure 2 | a Schematics of fabricated half-Au on n-Si and n-Si. **b** the measured current-voltage (I-V) curve of half-Au on n-Si and n-Si.

8. The power output, charge output and open-circuit voltage of the TENG should be characterized.

Response: As the tribo-effect and shadow-effect of S-TENG are connected in parallel,

the short-circuit current density of tribo-effect of S-TENG can be tested in a dark condition while the V_{oc} cannot be directly measured. This is because the parallel connection of the shadow-effect of S-TENG has a much lower internal resistance. For testing and estimating the open-circuit voltage and output of the tribo-effect of S-TENG, we connected the positive electrode of Keithley 6514 with one electrode of S-TENG and the negative electrode of Keithley 6514 was grounded. The V_{oc} and charge generated by the S-TENG in a single-electrode TENG connection is 80 V and 34 nC, respectively. The output power density of the S-TENG reached a maximum value of 0.28 μW cm^{-2} at a load resistance of 8 GΩ. We have added all of these results in the revision. The relevant analysis and discussion were added as follows:

"As shown in Supplementary Figure 8, the V_{oc} and charge generated by S-TENG in a single-electrode TENG connection is 80 V and 34 nC, respectively. The output power density of S-TENG reached a maximum value of 0.28 μW cm^{-2} at a load resistance of 8 GΩ. The related discussion is presented in Supplementary Note 8."

"**Supplementary Note 8. The V_{oc}, charge output, power density of the S-TENG in a single-electrode TENG connection**

As the TE and SE of S-TENG are connected in parallel, the J_{sc} of TE can be tested in a dark condition while the V_{oc} cannot be directly measured. This is because the parallel connection of the SE has a much lower internal resistance than TE. For testing the V_{oc} of TE, the positive electrode of Keithley 6514 was connected with one electrode of S-TENG and the negative electrode Keithley 6514 was grounded. The measurements were all carried out by moving stage of a force gauge towards and away from the S-TENG in a single-electrode TENG connection for accurate and repeatable motion control. As shown in Supplementary Figure 8a, the V_{oc} and charge is 80 V and 34 nC, respectively. Supplementary Figure 8b demonstrates the dependence of output peak current density and peak power density on load resistance ranging from 5 Ω to 10.5 GΩ. The maximum power is about 0.28 μW cm^{-2} when the resistance is 8 GΩ."

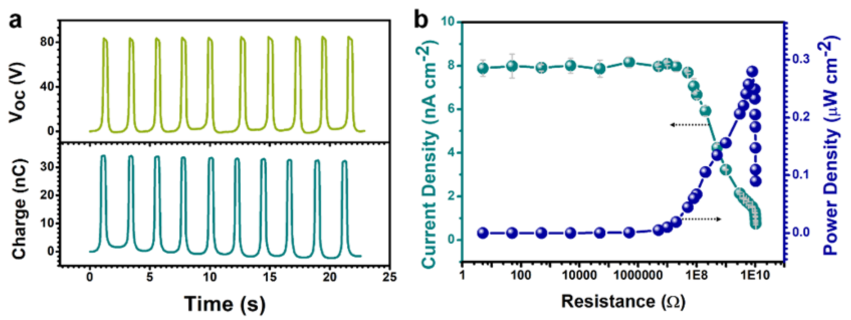

Supplementary Figure 8 | a The V_{oc} and charge output of the S-TENG in a single-electrode TENG connection. **b** Output power density of the S-TENG in a single-electrode TENG connection with different load resistances.

9. The capacitance of the capacitor in Figure 6 should be provided.

Response: The capacitance of the 5 fiber-supercapacitors in series used in Figure 6 was added in the revision as Figure 5h, 5i and Supplementary Figure 10. The relevant analysis and discussion were added as follows:

"Five F-SCs are connected in series to provides a tunable operating voltage for storing converted energy from S-TENG. Fig. 5h shows the CV curves of 5 F-SCs connected in series under different scan rate ranging from 100 mV s^{-1} to 2 V s^{-1}. The voltage could reach 5 V for the 5 F-SCs. The rectangular CV shape remains very well even at a high scan rate of 2 V s^{-1}, indicating its excellent rate capability. The GCDs of the 5 F-SCs connected in series are also shown in Supplementary Figure 10 and detailed discussion is in Supplementary Note 10. Fig. 5i represents the plots of obtained capacitances (C) of the 5 F-SCs against the discharge currents of the charge-discharge profiles. The C of the 5 F-SC connected in series under different current can be calculated from the GCD curves according to the following equation:

$$C = \frac{I\Delta t}{\Delta V} \tag{4}$$

Where I is the discharge current, Δt is the discharge time, ΔV is the potential window during the discharge process. The 5 F-SCs delivered the C of 0.416 mF at a discharge current of 20 μA while 1.096 mF at a discharge current of 200 nA."

"**Supplementary Note 10. GCDs of 5 F-SCs connected in series under different charging-discharging current**

Supplementary Figure 10a and 10b shows the GCD curves of the 5 F-SCs connected in series under current ranging from 1 μA to 20 μA and 200 nA to 800 nA. The voltages of these GCD curves vary from 0 to 5 V. It can be seen that the 5 F-SCs connected in series could be used to efficiently store electric energy even with very small charging current."

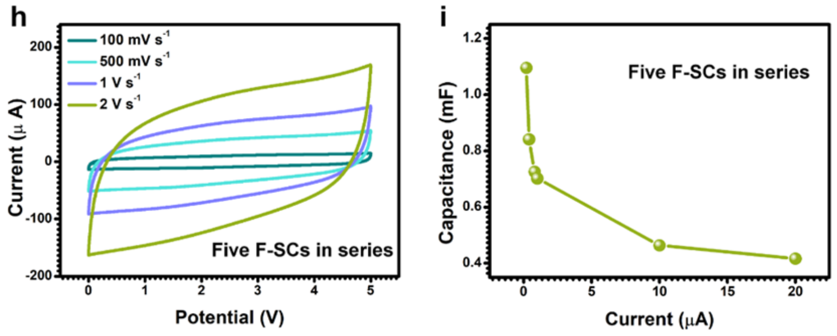

Figure 5h CVs of the 5 F-SCs connected in series at the different scan rates. **i** The summary plot of capacitance of 5 F-SCs connected in series versus different discharging current.

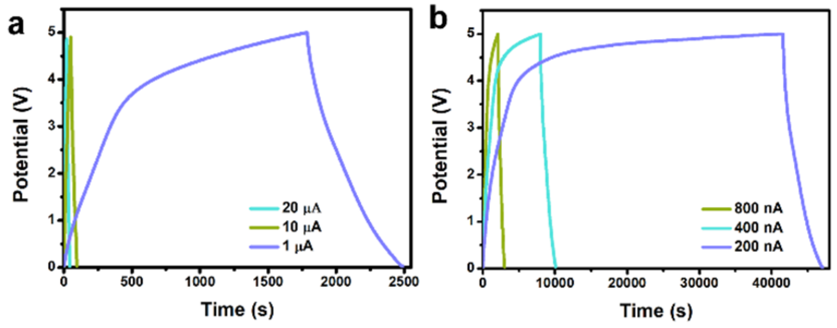

Supplementary Figure 10 | GCDs of 5 F-SCs connected in series at different current.

10. Please provide more information on the resistance measurement in Figure S3, such as the width of the film and the space between the two wiring points. Is the measurement based on pure Au film or using the structure of Au/n-Si.

Response: A Extech EX503 (Industrial Digital Multimeter, 1000V, 10A, 4000 Count) was used to measure the resistance of Au film on n-Si. The width of the film is 3 cm and the space between the two wiring points is about 4 cm. These details of resistance measurement are also added in revision.

11. Some typos exist: "wavelenth" in Figure 2c, "j/j15", "by amount of energy balls per kilometer" should use area unit, "which induces the charges on both electrodes reversing" the charges are not reversing from the figure, what is "Vop".

Response: Thank you for pointing these out. We have modified the errors in the revision. The revised Figure 2c is listed as below.

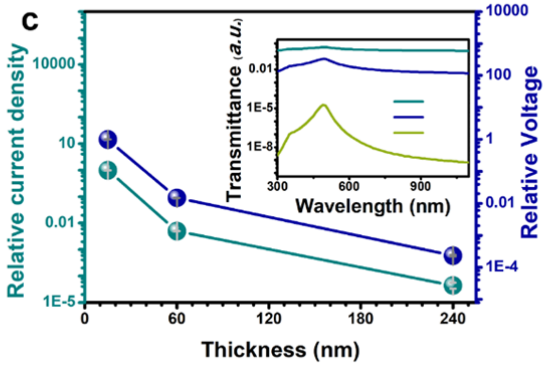

Figure 2c The Relative current density (J_{15}/J_{15}, J_{60}/J_{15}, J_{240}/J_{15}) and relative voltage (V_{15}/V_{15}, V_{60}/V_{15}, V_{240}/V_{15}) versus the thickness of Au film of the Au/n-Si system. Inset is transmittance of the Au film with different thicknesses.

12. "Under dark condition, Δσsc/dx in equation (3) can be considered as a constant, thus the Jsc will increase with the increasing velocity." The detailed discussion is not quite reasonable and necessary.

Response: Thank you for your suggestion. The as mentioned sentence has been deleted in the revision.

13. "In TE case, oppositely charged surfaces with a changing gap distance can be viewed as a capacitor with varying capacitance (CT)" The detailed discussion is not quite reasonable and necessary. Here, actually the capacitor in the model does not correspond to the surfaces with the changing gap.

Response: We have modified the sentence as "In TE case, the TENG can be viewed as a capacitor (C_T) using the capacitor model." in revision.

14. The rectification circuit in Figure 6d is not right. When SW2 is closed, it won't change AC to DC due to the wrong connection of the rectifier.

Response: We have modified the error in revision. The revised rectification circuit in Figure 6d is listed as below.

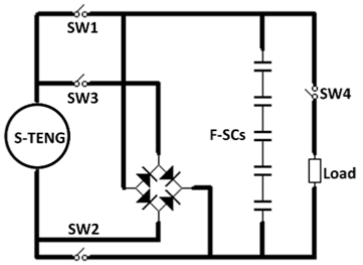

Figure 6d Inset Electric circuit of the S-TENG based self-charging power system and load.

15. What is the upper object in testing the data in Figure 2-4?

Response: In Figure 2a, nothing covered on the Au/n-Si system. In Figure 2b, 2c, 2d, 2e, a black paper was used as the upper object to cast shadow on Au/n-Si. In Figure 2f and 4a, the upper object is a transparent glass with and without black paper. In Figure 3d, 4b and 4c, 4d, the upper object is the moving stage of the force gauge. The relevant details of experiment have been added in the revision.

16. The data shown in Figure 6c are hard to understand. Why the current output with shadow is so low?

Response: The current density of shadow-effect shown in Figure 6c is about 34 μA cm^{-2} which is lower than Figure 2e shown (39 μA cm^{-2}). There are two possible factors (or experimental limitations) that could cause this current decrease:

(1) To simulate the environment of the ocean surface, a lamp was used to illuminate the S-TENG in an energy ball. The light intensity of lamp used in Figure 6c is lower than the sun simulator used in Figure 2e.

(2) The round PET shell may cause some light intensity loss when the light transmitting through it to the S-TENG.

In the shadow + wave case, a rectifier is connected with the S-TENG. There is a voltage drop about 0.7 V of the diode in the rectifier. So, the shadow-effect is only 0.2 V which was blocked. Therefore, the current output is low because this current is from TE not from SE.

We have added different colors in Figure 6c to distinguish light condition and dark condition. The revised Figure 6c is listed as below.

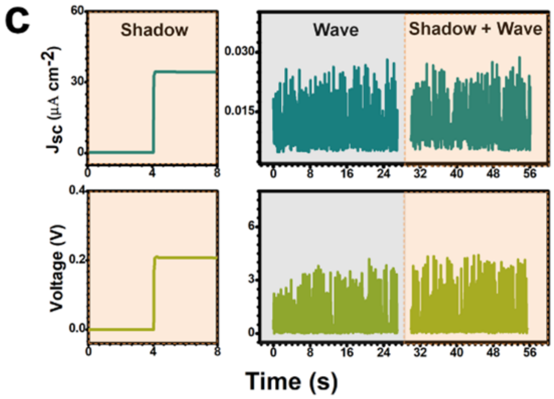

Figure 6**c** The measured J_{sc} and voltage of the S-TENG in the energy ball before and after being rectified.

Comments from Reviewer 2:

The authors have precisely addressed the comments. The manuscript should be accepted for publication.

Reviewer #1 (Remarks to the Author):

The authors have addressed most of the technical concerns. Although I reserve my views about the rationality of the device design, I will not object the publication of the manuscript. However, before the acceptance for publication, the following comments should be properly addressed. The authors should frankly and clearly discuss the limitations of the design and the device in the manuscript to avoid misleading.

(1) The impedance mismatching is obvious from the data, and Fig. 6b and Fig S8b can provide a rough compare. The impedance matching means the external load can acquire the largest power from the device under the same condition. When connect two devices with quite different internal impedances in parallel, it should be impossible to maximize the powering efficiency for both the two devices. Without a impedance matching, the device can still work (as the authors shown in Figure 6), but not the most efficient way for both the TE and SE parts. The author should frankly discuss it in the main manuscript to avoid misleading.

(2) As the authors name the device as 'S-TENG', the output of pure TE part is quite important and should be explicitly discussed with figures shown in the main manuscript (not in the SI). Meanwhile, the connection change in the present edition is not acceptable (Figure S8), it does not reflect the real power capability of the TE part without light (You will not change the connection in dark condition for output in real applications). The open circuit state means the device is open circuited from outside, which is not influenced by the internal impedance. So the open circuit voltage and other signals can still be measured without changing the connection.

(3) The material of the opaque object should be clearly described which will influence the TE. The contact area should also be described.

(4) In Supplementary Note 17, Supplementary Table 1, the author compared the device with some pure TENG devices. The difference in working mechanism (SE and TE) should be clearly noted and discussed to avoid misleading.

(5) In Figure 6c, it is still quite unclear. What is the step in the left panels, and what is the data in the right panels? Why the output with and without shadow are nearly the same in the right panels? Why the voltage in the right panel is so high and current in the right panel is so low compared to the left panels. Please check the data carefully.

(6) The charging rate without shadow in Figure 6d seems much higher than expected considering the charge output of the TE part. Please check the data and experiment.

(7) About the possible opposite current direction of TE and SE, please clearly discuss the limitations when they work simultaneously to avoid misleading.

Comments from Reviewer 1:

The authors have addressed most of the technical concerns. Although I reserve my views about the rationality of the device design, I will not object the publication of the manuscript. However, before the acceptance for publication, the following comments should be properly addressed. The authors should frankly and clearly discuss the limitations of the design and the device in the manuscript to avoid misleading.

1. The impedance mismatching is obvious from the data, and Fig. 6b and Fig S8b can provide a rough compare. The impedance matching means the external load can acquire the largest power from the device under the same condition. When connect two devices with quite different internal impedances in parallel, it should be impossible to maximize the powering efficiency for both the two devices. Without a impedance matching, the device can still work (as the authors shown in Figure 6), but not the most efficient way for both the TE and SE parts. The author should frankly discuss it in the main manuscript to avoid misleading.

Response: Thank you for your comments. We agree there's an impedance mismatching between tribo-effect (TE) and shadow-effect (SE). We added the discussion about the limitation of this in the revision to avoid misleading. However, we focus on the performance of the self-charging power system could be improved by using a hybrid nanogenerator. A strategy that charging the fiber-supercapacitors (F-SCs) with SE and TE separately is introduced in this work. First step: we used the SE of S-TENG charged F-SCs in a short time under illumination condition. Second step : After the F-SCs were charged to the highest voltage of SE, a rectifier that was connected with shadow-tribo-effect nanogenerator (S-TENG) can block the SE. Then the F-SCs could be charged to a higher voltage by TE. Thereby, the limitation of the impedance mismatching of S-TENG is avoided in the self-charging power system.

The added discussion is as follows:

"Comparing the results shown in Fig. 4f, the impedance of SE is smaller than TE. When connecting SE and TE with quite different internal impedances in parallel, the powering efficiency cannot be improved directly. To this end, five F-SCs in series are connected with S-TENG to form a self-charging power system."

“Thereby, the limitation of the impedance mismatching of S-TENG is avoided in the self-charging power system.”

2. As the authors name the device as ‘S-TENG’, the output of pure TE part is quite important and should be explicitly discussed with figures shown in the main manuscript (not in the SI). Meanwhile, the connection change in the present edition is not acceptable (Figure S8), it does not reflect the real power capability of the TE part without light (You will not change the connection in dark condition for output in real applications). The open circuit state means the device is open circuited from outside, which is not influenced by the internal impedance. So the open circuit voltage and other signals can still be measured without changing the connection.

Response: Thank you for your comments. We checked the experiment and verified that the open-circuit voltage of the TE cannot be directly measured when the connection was not changed. In an ideal case, the open-circuit state means the device is open circuited from outside. However, the open-circuit voltage was experimentally tested by using a large resistance to simulate the open circuit state. According to Thévenin's theorem and Kirchhoff’s rules, the internal resistance will affect the test results in the closed loop of testing. Therefore, the measured open-circuit voltage is mainly from SE with a smaller internal resistance even in the dark condition when the SE and TE were connected in parallel.

In addition, S-TENG, which is a hybrid nanogenerator, is not achieved by simple pasting of TENG and SEG together. The SE part cannot be removed to test TE. So we used a connection change method to test the output of a single-electrode triboelectric generator under the same condition. The result is expected to roughly estimate the output of TE which is based on a single-electrode mode. As your suggestions, the TE output estimate is explicitly discussed with figures shown in the main manuscript. The explanation of the limitations is also added in the revision as follows:

“As the TE and SE of S-TENG are connected in parallel, the J_{sc} of TE can be tested in a dark condition while the V_{oc} cannot be directly measured. This is because the parallel connection of the SE has a much lower internal resistance than TE. In addition, the SE part cannot be removed to test TE. So a connection change method is used to test the output of a single-electrode TENG under the same condition. The result is expected to roughly estimate the output of TE which is based on a single-electrode mode. The positive electrode of Keithley 6514 was connected with one electrode of S-TENG and the negative electrode Keithley 6514 was grounded. The measurements were all carried

out by moving stage of a force gauge towards and away from the S-TENG in a single-electrode TENG connection for accurate and repeatable motion control. As shown in Fig. 4e, the V_{oc} and charge is 80 V and 34 nC, respectively. Fig. 4f demonstrates the dependence of output peak current density and peak power density on load resistance ranging from 5 Ω to 10.5 GΩ. The maximum power is about 0.28 μW cm^{-2} when the resistance is 8 GΩ."

3. The material of the opaque object should be clearly described which will influence the TE. The contact area should also be described.

Response: Thank you for your suggestions. We added the details of the materials of the opaque object and contact area in the revision as follows:

"Polyurethane foam is pasted on the surface of moving stage to contact with the surface of S-TENG. The contact area is about 7.5 cm^2."

4. In Supplementary Note 17, Supplementary Table 1, the author compared the device with some pure TENG devices. The difference in working mechanism (SE and TE) should be clearly noted and discussed to avoid misleading.

Response: Thank you for your suggestions. We added the S-TENG performed the largest current output or peak power than other pure TENG devices due to the SE in revision.

The relevant discussion was revised in the revision as follows:

"The characteristics of S-TENG based energy ball compared with other pure TENG based technologies for energy harvesting from ocean in Supplementary Table 1 and detailed discussion is presented in Supplementary Note 17."

"Comparing with the other technologies, the S-TENG in this work have the largest current output or peak power per one unit due to the shadow-effect, respectively."

5. In Figure 6c, it is still quite unclear. What is the step in the left panels, and what is the data in the right panels? Why the output with and without shadow are nearly the same in the right panels? Why the voltage in the right panel is so high and current in the right panel is so low compared to the left panels. Please check the data carefully.

Response: The current density and voltage shown in left panels of Figure 6c is generated only by SE of S-TENG, which related to the step of closing Switch 1, 2 in circuit diagram (inset Figure 6d). In this step, the voltage of F-SCs increased sharply from 0 to 0.2 V in 4 s (Figure 6d). The right panels data is collected by opening Switch 1, 2 and closing Switch 3. The S-TENG is connected with a rectifier in this step.

The output of S-TENG in the right panels are all tested after connecting S-TENG with

a rectifier. There is a voltage drop (0.7 V for Si diode) in the rectifier, only allowing the voltage higher than it to pass. In this case, the output of SE will be blocked by the rectifier due to its lower voltage (0.2 V) than this barrier. Thereby, the output with and without shadow are nearly the same.

Comparing to the left panels, which is without a rectifier, the voltage in the right panel is higher and current is lower. This is because the output of right panel mainly depends on TE, the output of SE is blocked by the rectifier.

The related analysis and discussion in the revision are rephrased as follows:

"The output of the S-TENG connected with F-SCs in the self-charging power system is shown in Fig. 6c while the circuit diagram of the whole system is shown inset in Fig. 6d. The J_{sc} and voltage shown in left panels of Fig. 6c were generated by only SE of S-TNEG under the light illumination condition, which related to the step of closing Switch (SW) 1 and SW2. In this stage, the voltage of F-SCs increased sharply from 0 to 0.2 V in 4 s by only casting the shadow of Al ball. When the SW1 and SW2 were opened and SW3 was closed simultaneously, a rectifier was connected with S-TENG to transfer current from AC to DC. The S-TENG can provide a much higher output voltage to compensate the limitation of SE after rectified when it is stimulated by waves. The rectified output before and after illuminating the energy ball were also tested. As shown in the right panel of Fig. 6c, the rectified voltage of S-TENG is about 4.1 V, which is the same under dark or illumination conditions. This is because there is a voltage drop (0.7 V for Si diode) in the rectifier, only allowing higher voltage to pass. The output of SE will be blocked by the rectifier due to its lower voltage (0.2 V) than this barrier."

6. The charging rate without shadow in Figure 6d seems much higher than expected considering the charge output of the TE part. Please check the data and experiment.

Response: The charging current without shadow shown in Figure 6c is about 200 nA. We think the charging rate without shadow is reasonable after comparing it with the galvanostatic charging rate of 5 F-SCs connected in series. As shown in Figure 6d, the charging time from 0 V to 0.3 V without shadow is 386 s, which is longer than the galvanostatic charging time of 5 F-SCs connected in series at 200 nA (100 s). This because the pulse current of the S-TENG causes lower charging efficiency than a continuous current. We added the details of the galvanostatic charging curve of 5 F-SCs connected in series at 200 nA, 400 nA, and 800 nA from 0 V to 0.3 V in revision, which is listed as below.

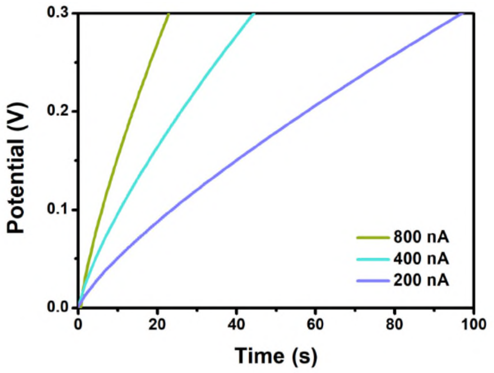

Supplementary Figure 9 | c Details of the galvanostatic charging curve of 5 F-SCs connected in series at 200 nA, 400 nA, and 800 nA from 0 V to 0.3 V.

7. About the possible opposite current direction of TE and SE, please clearly discuss the limitations when they work simultaneously to avoid misleading.

Response: Thank you for your comments. We added the discussion about these two effects work simultaneously in revision. The relevant discussion was added in the revision as follows:

"An opposite current direction of TE and SE could be generated when they work simultaneously under the light condition. However, the current generated from SE is much larger than TE, so the S-TENG which coupled TE and SE is in the same current direction of SE."
